# Quality assessment and interference detection in targeted mass spectrometry data using machine learning

**DOI:** 10.1186/s12014-018-9209-x

**Published:** 2018-10-06

**Authors:** Shadi Toghi Eshghi, Paul Auger, W. Rodney Mathews

**Affiliations:** OMNI-Biomarker Development, Genentech Inc., South San Francisco, CA 94080 USA

**Keywords:** Targeted proteomics, Quantitative, Mass spectrometry, Bioinformatics, Automated analysis, Interference detection, Quality control, Machine learning

## Abstract

Advances in the field of targeted proteomics and mass spectrometry have significantly improved assay sensitivity and multiplexing capacity. The high-throughput nature of targeted proteomics experiments has increased the rate of data production, which requires development of novel analytical tools to keep up with data processing demand. Currently, development and validation of targeted mass spectrometry assays require manual inspection of chromatographic peaks from large datasets to ensure quality, a process that is time consuming, prone to inter- and intra-operator variability and limits the efficiency of data interpretation from targeted proteomics analyses. To address this challenge, we have developed TargetedMSQC, an R package that facilitates quality control and verification of chromatographic peaks from targeted proteomics datasets. This tool calculates metrics to quantify several quality aspects of a chromatographic peak, e.g. symmetry, jaggedness and modality, co-elution and shape similarity of monitored transitions in a peak group, as well as the consistency of transitions’ ratios between endogenous analytes and isotopically labeled internal standards and consistency of retention time across multiple runs. The algorithm takes advantage of supervised machine learning to identify peaks with interference or poor chromatography based on a set of peaks that have been annotated by an expert analyst. Using TargetedMSQC to analyze targeted proteomics data reduces the time spent on manual inspection of peaks and improves both speed and accuracy of interference detection. Additionally, by allowing the analysts to customize the tool for application on different datasets, TargetedMSQC gives the users the flexibility to define the acceptable quality for specific datasets. Furthermore, automated and quantitative assessment of peak quality offers a more objective and systematic framework for high throughput analysis of targeted mass spectrometry assay datasets and is a step towards more robust and faster assay implementation.

**Electronic supplementary material:**

The online version of this article (10.1186/s12014-018-9209-x) contains supplementary material, which is available to authorized users.

## Background

Targeted proteomics using mass spectrometry (MS) is a powerful technology for quantitation of candidate biomarkers for clinical research and development [1, 2]. Targeted MS methods such as multiple reaction monitoring (MRM) [1, 3–5] and parallel reaction monitoring (PRM) [6, 7] enable multiplexing of tens or hundreds of target proteins to report absolute quantitative levels of putative biomarkers using stable-isotope labeled standards (SILs) [8, 9], eliminating the need for costly and lengthy antibody development [1]. Targeted MS has found appeal in all phases of biomarker development from discovery to validation [10–13]. In the biomarker discovery phase, it is essential to consider the pathology of the disease or the mechanism of action of the therapeutic under investigation to create a list of candidates for a targeted panel. Unlike what is customary in shotgun proteomics, limiting the candidate biomarkers to proteins that are biologically relevant at early stages of biomarker discovery increases confidence in the utility of biomarkers that are shown to be of value in a targeted MS workflow. This selectivity in targets also reduces the chance of false positive markers due to multiple hypotheses testing, a common caveat in shotgun proteomics [14]. Moreover, the increased specificity achieved by the targeted approach combined with its inherent sensitivity provides a promising tool for biomarker validation. All of these advantages have increased the utility of mass spectrometry-based assays in clinical research beyond the more well-established applications of pharmacokinetics [15, 16] and toxicology [17].

**Fig. 1:**
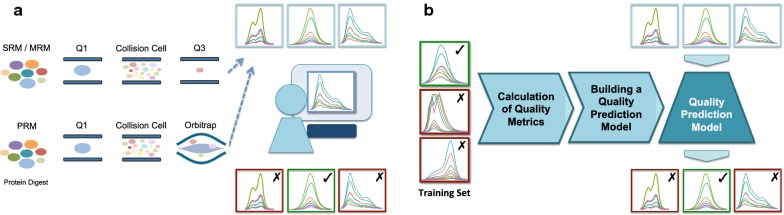
Targeted MS workflow with manual and automated peak quality assessment. **a** Targeted MS workflows such as selected reaction monitoring (SRM), multiple reaction monitoring (MRM) and parallel reaction monitoring (PRM) generate chromatographic peak groups, representing relative abundance of transitions (peptide and fragment ion pairs). Several factors such as poor chromatography, interference and matrix effects can compromise the quality of these peaks and subsequently the accuracy of the reported quantitative results. Therefore, the peaks undergo manual inspection by a trained analyst to identify such anomalies, a process that is oftentimes time-consuming and subjective. **b** In the developed automated QC process, a dataset of pre-annotated peaks is used to build a model to predict the quality of the chromatographic peaks generated from SRM, MRM and PRM workflows

With the incorporation of mass spectrometry-based targeted proteomics experiments into larger scale studies and as the throughput of instruments improves, the need for objective, reproducible and scalable solutions for data analysis has grown in parallel. Analysis of targeted proteomics data often starts with manual inspection of individual peaks to ensure acceptable data quality [12, 18]. This quality assessment step, performed independently from system suitability monitoring which tracks the performance of the instrument [19], focuses on uncovering interference or matrix effects for individual peptides and transitions (Fig. 1a). Several factors are taken into account for assessment of peak quality including consistency of retention time for each target peptide, acceptable chromatography as reflected by peak shape, consistency of transition ratios across samples as well as reasonably robust quantitation as demonstrated by the variability of peak areas and peak area ratios [12, 20–22]. In cases of interference or suboptimal peak picking, the analyst may adjust the boundaries of the peaks to remove interference and improve the specificity of the measurements. If the adjustment of the boundaries is insufficient to effectively remove interference, the analyst may remove the peptide from list of reported quantities. Manual assessment of peaks is time-consuming, requires extensive training of the analysts, and is subject to inter and intra-operator variability. Furthermore, decentralized quality assessment and data analysis could further complicate the transferability and reproducibility of targeted MS assays. Therefore, establishing an analytical framework for quality assessment of chromatographic peaks in a standardized manner may mitigate issues with large-scale mass spectrometry-based studies in the longer term.

The quality assessment process for targeted MS assays can be broken down into several components and consists of quality control (QC) at instrument, run, peptide and transition levels. Some of the currently available software tools have implemented measures to simplify this process. Several commercial and open source software and computational tools have been made available to the community for development of targeted MS assays and analysis of the results. Vendor agnostic software tools such as Skyline [18] and vendor-specific software tools such as MultiQuant provide a toolset, interactive user interference and a suite of visualizations for the operator or analyst to create methods, integrate peak boundaries, and perform quantitative analysis of the results. Longitudinal system suitability monitoring tools such as MSstatsQC [23] and AutoQC [24], which integrate with Skyline through Panorama, enable monitoring of the instrument performance over time and thereby facilitate earlier intervention for troubleshooting at the instrument level. Statistical tools such as MSstats [25] uses overall variations in the intensity of analytes across multiple runs for quality assessment at individual run level to identify issues with injection or sample quality. Additionally, Skyline takes advantage of multivariate statistical tools such as mProphet [21] to optimize peak picking and integration and minimize the required manual peak adjustment. mProphet is a comprehensive automated platform for verification of targeted MS data; however it requires acquisition of decoy transitions, which is not always practical in a high-throughput workflow. Tools such as AuDIT [26] aim to identify interference in transitions and therefore simplify quality assessment of targeted proteomics data, although due to small number of transitions, this method has limited statistical power. Combined, these tools have drastically simplified the development of targeted MS assays and subsequent analysis of results. However, the burden of selecting peptides and transitions for a targeted panel and quality assessment of the peaks mainly fall on the shoulder of the analyst.

Herein, we present TargetedMSQC, a computational tool to facilitate high-throughput data analysis for targeted mass spectrometry. This tool has been developed with the main objective of automating quality assessment of chromatographic peaks to identify poor chromatography or interference (Fig. 1b). TargetedMSQC takes advantage of well-established peak quality measures such as retention time consistency and full-width at half-max (FWHM) as well as newly introduced metrics such as peak jaggedness, peak modality and isotope ratio consistency. TargetedMSQC utilizes these variables and multivariate statistical methods for the identification and demarcation of peaks with interference or suboptimal chromatography. Using TargetedMSQC, the user first provides a small subset of the dataset as a training set, where chromatographic peaks are manually inspected and the ones with poor quality are flagged. TargetedMSQC uses machine learning to build a predictive model that links the peak quality measures with the quality status reported in the training dataset. Finally, the tool applies this model on the remaining peaks to predict and report the peak quality for the whole dataset. Here, the application of TargetedMSQC is demonstrated on a targeted proteomic MRM assay developed to study longitudinal dynamics of cerebrospinal fluid (CSF) biomarkers of Alzheimer’s diseases (AD) [27]. By enabling automated flagging of low quality peaks, TargetedMSQC eliminates or lessens the burden of manual inspection of all peaks for boundary correction or removal from the list of reported quantities, improving the throughput of the analytical pipeline. It also provides a standardized framework to objectively and quantitatively evaluate quality across transitions, peptides and samples providing a means for mass spectrometry and contract laboratories to monitor the performance of their proteomics assays. This capability could be particularly valuable in large scale, multi-center or cohort studies where standardization of data quality control could not only expedite the analysis, and simplify optimization of resources, but also improve the analytical reproducibility, traceability and continuity across multiple laboratories and studies.

## Methods

### Materials and reagents

CSF samples were purchased from PrecisionMed, Inc. (San Diego, CA). All donors provided informed consent for use of these studies with institutional review board approval for human collection protocols. Detailed description of sample collection protocols are published previously [27]. All reagents were purchased from Sigma (St. Louis, MO), unless stated otherwise. Stable-isotope-labeled AQUA™ peptides were purchased from Cell Signaling Technologies, (Danvers, MA) for method development and to generate calibration curves.

### Sample preparation and mass spectrometry analysis

#### CSF biomarkers of AD

This study was conducted by Wildsmith et al. [27] and the dataset was kindly provided by the authors for the evaluation of TargetedMSQC. Sample preparation and targeted MS analysis of CSF tryptic digests are described in detail in the original publication [27]. Briefly, samples were concentrated to 30 μL using a 3 kDa Amicon centrifugal concentrator (Millipore, Billerica, MA), denatured in 40% trifluoroethanol (TFE) prepared in 100 mM triethylammonium bicarbonate (TEABC) for 1 h at 37 °C. Following denaturation, samples were reduced with 5 mM dithiothreitol (DTT) for 30 min at room temperature (RT) and then alkylated with 20 mM iodoacetamide (IAM) for 30 min at RT in the dark. This reaction was quenched with an additional 5 mM DTT for 15 min at RT. Samples were diluted to 10% TFE with 100 mM TEABC followed by digestion with trypsin at a ratio of 1:25 for 18 h at 37 °C. The digestion was stopped by addition of formic acid. The final volume of all digests was measured. An aliquot of the total digest (48 μL) was spiked with 2 μL of a mixture of stable-isotope-labeled AQUA™ peptides prior to LC-MS/MS analysis. Heavy AQUA™ peptides to be spiked into samples were diluted and pooled before use to concentrations that were within tenfold of endogenous protein levels and ranged from 2 to 100 fmol/μL depending on analyte. For method development and generation of calibration curves, light AQUA™ peptides were spiked into the solution of tryptically digested BSA and heavy AQUA™ peptides at varying concentrations.

For LC-MS/MS analysis of the CSF tryptic digests, 2 μL of each sample was loaded onto a nanoAcquity UPLC (Waters, Milford, MA) at 5 μL/min in 99.5% buffer A (0.1% formic acid)/0.5% buffer B (acetonitrile/0.1% formic acid) and then desalted on a Symmetry^®^ C18 trap column (180 μm × 20 mm, 5 μm) (Waters) for 3 min prior to separation on a BEH130 C18 (100 μm × 100 mm, 1.7 μm) (Waters) at a flow rate of 1 μL/min over 60 min. The gradient formation went from 2% buffer B to 30% buffer B in 40 min followed by 30–98% buffer B in 10 min. A 98% solution of buffer B was used to wash the column for 5 min prior to reequilibration at 2% buffer B for 5 min. Peptides were detected by a QTRAP^®^ 5500 (AB SCIEX, Framingham, MA) equipped with an Advance Captivespray™ source (Michrom Bioresources, Inc. Auburn, CA). Scheduled MRM methods were prepared using Skyline v1.3 and imported into Analyst 1.5.2 (ABSCIEX). The QTRAP^®^ 5500 was operated in positive ion mode using the scheduled MRM.

### Engineering QC features for peak quality assessment

Thirty-two features were calculated or engineered to quantify various attributes pertaining to peak quality, including peak area and transition ratio, full-width at half-max (FWHM), symmetry, peak similarity, modality, co-elution and retention time. A complete list of these features and their definitions is provided in Additional file 1: Table S1. The features were calculated using custom R scripts implemented as part of the TargetedMSQC package. The scripts accept chromatograms and peak boundaries, exported from Skyline, as input and generate a comma separated value file (features.csv) with the calculated features. Depending on the nature of the QC feature, it may be calculated at one or more levels. For example, jaggedness is reported at transition, isotope and peak group levels, while max intensity is only reported at transition level. Features at transition level are reported for peptide, fragment ions and isotope label trios. Features at peak group level are reported for individual peptides. Features at transition pair level are reported for peptide and fragment ion pairs. Features at isotope level are reported for peptide and isotope label pairs. It should be noted that the model predicts the peak quality for each endogenous and spiked-in standard transition as a pair. Therefore, the QC features that correspond to a specific label type, i.e. features at transition and isotope level, should be calculated and provided to the model for both endogenous and spiked-in standards, which brings the total number of features to 52.

### Creating the training datasets

The training dataset is a set of peaks with manually annotated quality status, which is used as a guide to build a predictive peak quality model. A subset of the runs in each dataset is randomly selected for manual annotation and the monitored transitions for quantification of all of the peptides on the MRM panel are annotated in this subset of runs to create a training dataset. This ensures that all of the peptides and transitions in the panel are reasonably represented in the training set. The required number of runs for training is determined empirically. Cross validation and resampling are used to estimate the model performance and its confidence interval for an increasing number of runs in the training set until our desired model performance is achieved. Learning curves are plotted to help determine the size of the training dataset to either achieve minimum acceptable model performance or observe a plateau in the curve. In this study, each training set contains a panel of 36 peptides and 144 unique peptide transitions, measured in multiple samples. The runs were imported into Skyline daily and the chromatograms were exported as a.tsv file. The peak boundaries were saved as a.csv file through the export and report option in Skyline. Inputs to TargetedMSQC are exported from Skyline; therefore this tool is independent of MS vendor file formats. The training set was created by merging chromatograms and peak boundaries using TargetedMSQC. Additionally, transitions with missing isotope pairs and peaks with missing boundaries were removed. The transition peaks, characterized by run, peptide and fragment ion, were manually inspected by an analyst and annotated by ‘ok’ or ‘flag’ labels. The following qualities were used during manual inspection to flag low quality peaks: bimodal peaks, peaks suffering from poor chromatography as indicated by sharp and jagged edges, severe tailing, or high background, peaks exhibiting signs of interference e.g. by introducing inconsistence transition ratios between multiple runs or endogenous and standard isotopes, and finally peaks with low standard isotope signal intensities. The annotated dataset was reviewed by two mass spectrometry analysts. The agreement between analysts ranged from 80 to 95% depending on overall quality of the data and complexity of the QC process. Before training the model, the analysts reviewed the data to reach agreement on the controversial peaks.

### Building the predictive quality assessment model

To build the quality prediction model for each study, the QC features were first calculated for the peaks in the training set. To optimize the predictive peak quality model, several machine learning methods were evaluated. These models include: support vector machines (SVM) with linear and polynomial kernels, regularized logistic regression, regularized random forest (RRF), and K-nearest neighbor (KNN). The caret package in R [28] was used to train the models and evaluate their performance. Twenty percent of the training dataset was randomly selected as validation set and left out from the training process to estimate the performance of the models on unseen data. The features were mean centered and scaled by diving by the standard deviation before being used for training. Repeated tenfold cross validation (3 repeats) was applied to the remainder of the training set to minimize over-fitting. Parameter tuning was employed to optimize the model. The model offering the highest accuracy was used for subsequent analysis.

### Peak quality assessment of targeted MS datasets using the predictive model

Once the model with the highest performance was selected for each dataset, it was applied to the complete dataset. QC features were calculated for every peak in the dataset and the model was applied to these features using the built-in functionalities in TargetedMSQC to flag transition peaks with poor quality. The results of this assessment were reported in data tables as well as a.pdf report that summarizes the output at sample and peptide levels. Additional visualizations were generated by TargetedMSQC and included in the report to help identify transitions that show interference or poor chromatography across multiple samples.

### Developing the TargetedMSQC R package

This analysis has taken advantage of several scripts to clean up the Skyline chromatograms and peak boundaries, calculate the engineered QC features, create templates for the training dataset, merging the training dataset with QC features and train and apply the peak quality model. All these scripts and examples on how to use them are included in the TargetedMSQC tool, an open source R package. This package is freely available on GitHub (https://github.com/shadieshghi/TargetedMSQC).

## Results

### Engineered QC features quantify the quality of chromatographic peaks

Poor chromatography and interference compromise the quality of a peak. The factors most affected by this compromise are peak shape, symmetry, jaggedness, FWHM, retention time shift, and the consistency of the ratios of peptide transitions. TargetedMSQC calculates quality features that have been engineered to capture changes in peak quality. A total of thirty-two features have been developed to describe the peak quality. These features can be categorized into nine main groups of jaggedness, peak similarity, symmetry, FWHM, modality, shift, intensity, area ratio and retention time. Additional file 1: Table S1 lists these QC features and their definitions in detail.

**Fig. 2:**
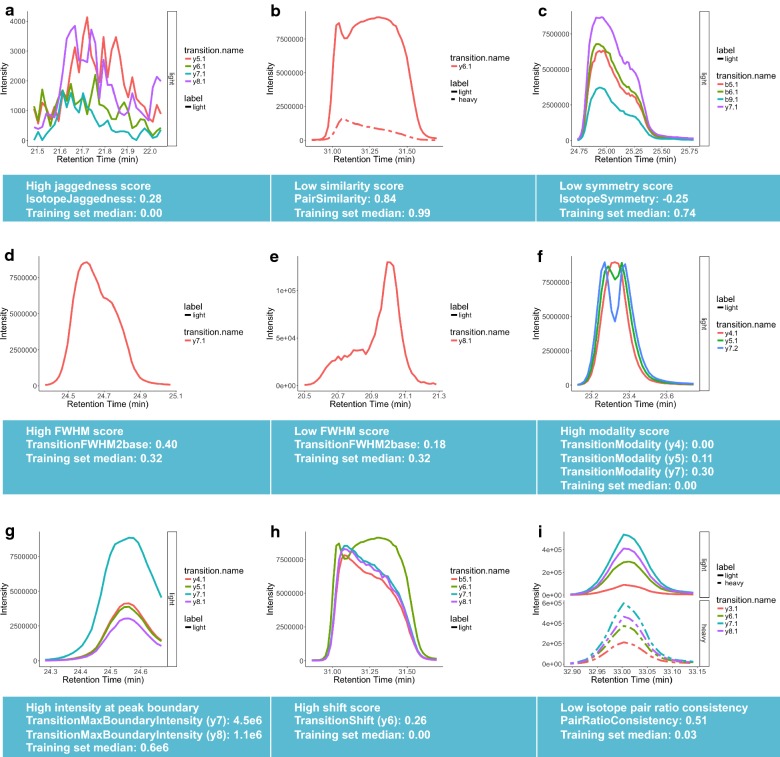
Engineered QC features capture anomalies in peak quality. Representative peaks and features are depicted from the following QC metric groups: Jaggedness (**a**), similarity (**b**), symmetry (**c**), FWHM (**d**, **e**), modality (**f**), intensity (**g**), shift (**h**) and area ratio (**i**). The represented examples provide cases of abnormal values for each feature. The medians of feature values over the training dataset are provided as a reference point for a peak that is of acceptable quality. Definitions of the metrics in this figure are described in Additional file 1: Table S1

Figure 2 depicts several representative examples of chromatographic peaks and their corresponding QC features. For instance, jaggedness is defined as the fraction of time points across a peak where the signal changes direction, excluding the peak apex and therefore, returns a value of 0.0 for a smooth peak, whereas jaggedness scores closer to 1.0 indicate a noisy peak (Fig. 2a). Similarity score between two peaks is determined by the Pearson correlation coefficient between the peak intensities, which yields a value of 1.0 for highly similar peaks that mirror the shapes of one another and lower values for pairs with differences in peak shapes (Fig. 2b). Symmetry score for a peak quantifies how symmetric a peak is along its time midpoint and yields values close to 1.0 for perfectly symmetric and lower scores for asymmetric peaks (Fig. 2c). FWHM and FWHM to peak base width ratio are well-known quality metrics for chromatographic peaks that can deviate from a normal range due to interference resulting in peak shoulders or poor chromatography (Fig. 2d, e). Modality score is defined as the largest dip in the peak, normalized by peak height. For example, the three transitions represented in Fig. 2f show varying levels of bimodal behavior with modality scores ranging from 0.0 for the smooth y4 transition to 0.3 for the highly bimodal y7 transition. Peaks with high intensities at the peak boundary may be subject to interference and therefore the peak intensity at the boundary was introduced as a QC feature in this schema (Fig. 2g). Additionally, all the fragment ions that belong to the same peptide must co-elute. Therefore, observing a shift in the elution of one transition flags the peak for manual reanalysis. This shift in elution is quantified by shift score as shown in Fig. 2h. Finally, an important attribute of peak groups in a mass spectrometry experiment is consistency of relative ratios of transitions not only between endogenous and standard isotopes, but also across samples. Pair ratio consistency is one of the metrics that quantifies this feature and is able to identify transitions where this ratio deviates from expected values as shown for y3 transition in Fig. 2i.

An ensemble of these QC features enables a quantitative way to evaluate peak quality. For instance, a peak that may suffer from nothing more than tailing will result in low symmetry scores while showing close to perfect scores for all the other features, whereas interference in a peak may manifest in poor similarity, modality, shift and isotope ratio consistency scores. TargetedMSQC uses supervised learning based on a set of manually annotated peaks with similar features to train a model that is capable of predicting peak qualities in new samples, which is particularly useful for performing QC on large datasets.

### Peak quality assessment of CSF biomarkers in artificial matrix

To demonstrate the feasibility of developing a predictive peak quality model, our approach was first applied to a dataset of AQUA™ peptides of CSF candidate biomarkers spiked into bovine serum albumin (BSA) as an artificial CSF matrix. This experiment was used for optimization of the CSF biomarker panel. Samples were analyzed by an MRM panel of 144 unique peptide transitions to quantify 36 peptides using stable-isotope labeled internal standards.

#### Training dataset

Four runs were used to generate the training set containing a total of 288 peak groups (144 endogenous and 144 spiked-in internal standards) and 576 transition pairs. A minimum accuracy of 90% was agreed upon as acceptable performance in this study. The learning curve for this dataset (Additional file 1: Figure S1) shows that at least 400 transition pairs are required to achieve this performance. Of the 576 peaks in the training set, 199 were flagged by an analyst for quality issues pertaining to interference, poor chromatography or low signal. Characteristics such as bimodality, jagged edges, severe tailing, variability in transition ratios and low spiked-in standard signal were used by the analyst to identify and flag low quality peaks. The remaining 377 peaks were considered of acceptable quality.

The training dataset consists of a list of peaks identified by run, peptide sequence, precursor charge, fragment ion, product charge and quality status, which has been determined manually by an analyst. Peak quality metrics were calculated for the training set; violin plots for the distribution of each metric are shown in Additional file 1: Figure S2. The training set was used as a guide to build a predictive model that can identify low quality peaks based on an ensemble of QC features such as peak symmetry, similarity, modality, jaggedness, co-elution and FWHM. Distribution of features for this dataset (Additional file 1: Figure S2) provides a quick glance at the overall quality of peaks in this set as judged by the analyst. As shown in Additional file 1: Figure S2, majority of the features exhibit a pattern in relationship to the quality status determined through manual inspection. For example, flagged peaks tend to have higher scores for features such as modality, shift, isotope pair ratio consistency, coefficient of variation and intensity at the peak boundary and lower scores for features such as symmetry, similarity, and correlation between peak area ratios of endogenous and standard isotopes. However, there is a not a single feature that can effectively discriminate between high and low quality peaks, further emphasizing the importance of utilizing multiple features and multivariate statistical methods to identify peaks with poor quality. The training dataset in this study contains peaks on a diverse quality spectrum. Therefore, by including a diverse set of peaks in the training dataset the capability of the predictive model to identify a wider range of quality issues is enhanced.

#### Predictive peak quality model development

After calculating the QC features for the peaks in the training dataset, several supervised learning methods were examined to construct a peak quality predictive model: Regularized logistic regression, regularized random forest (RRF), K-nearest neighbor (KNN) and support vector machine (SVM) with linear and polynomial kernels. When applicable, regularization was used to simplify the model and reduce the risk of overfitting. The models were built based on 80% of the data randomly sampled from the training set and their performances were further verified by testing the models on the remaining 20% of the data, named validation subset. The performances of the models were estimated by comparing the predicted peak qualities for each model with the observed qualities as determined by the analyst.

The performances of these models are summarized in Additional file 1: Table S2 and Figure S3. Among the five investigated methods, regularized random forest achieved the highest performance with an accuracy of 94.5% to distinguish flagged from high quality peaks in the training subset, and sensitivity and specificity rates of 97.4% and 98.7% on the validation subset, respectively. The negligible difference between model classification accuracy on the training and validation subsets suggests minimal overfitting. The performance of the other four models was reasonable as well, ranging from accuracies of 89.8% for linear SVM to 93.4% for the KNN models; however most of them showed low sensitivity for detection of poor quality peaks in the validation subset. In general, considering that training of an RRF model is computationally expensive, it takes longer to build an RRF model compared to the other methods employed in this study. For example, training the RRF model, including cross validation and parameter tuning, took ~ 40 min on a single processor. In comparison, training of the KNN model with the same cross validation method and similarly sized parameter tuning set was six times faster. Therefore, alternative methods may still be worthwhile to explore for different applications or datasets. Additional file 1: Figure S4 shows the relative importance of the QC features in determining the quality outcome for a peak in the RRF predictive model. As shown in this figure, compared to jaggedness, shift and modality metrics, features that quantify peak intensity, isotope ratio consistency, peak similarity and FWHM seem to play a more crucial part in determining the outcome of the predictive peak quality model. One explanation could be that this particular dataset has very few jagged or bimodal peaks and therefore, metrics such as jaggedness and modality do not play a defining role in distinguishing high and low quality peaks.

The high sensitivity and specificity of the model for distinguishing low quality peaks suggest that the developed QC features are capable of capturing the peak quality in a similar way to manual inspection of the peaks. These features can subsequently be leveraged by machine learning to make predictions about peak quality in unseen data, as long as the training set provides a fair representation of the range of peaks in the dataset under examination by the model. This example demonstrates great agreement between the manual inspection calls and the model outcome. In this example, the rare instances of disagreement between the model and the analyst are marginal cases (Additional file 1: Figure S5) where the peak quality lies in the gray zone and does not have a tangible effect on the quantitative outcome of the experiment.

### Peak quality assessment of longitudinal CSF biomarkers of AD dataset

To further evaluate the practicality and performance of the proposed quality assessment framework, TargetedMSQC was applied to a dataset from a longitudinal study of candidate progression biomarkers of Alzheimer’s disease in procured CSF samples of AD patients. Samples were analyzed by the same MRM panel in the previous example, quantifying 36 peptides using stable-isotope labeled internal standards.

#### Training dataset

The original dataset included 70 runs of a panel of 36 peptides in procured CSF samples from patients with Alzheimer’s disease. Eight runs were selected at random to be annotated for the training dataset. Poor quality peaks including, jagged peaks, peaks with high background or interference were flagged in the training dataset. This resulted in a set of 1128 transition pairs, with 615 ‘ok’ and 513 ‘flagged’ peaks. The learning curve for this dataset (Additional file 1: Figure S6) shows that at ~ 900 transition pairs the curve plateaus. The engineered features were calculated for each transition pair in the training set as shown in Additional file 1: Figure S7. The patterns for the distribution of features were as expected. Flagged peaks had higher jaggedness, modality, shift, peak area CV% and lower symmetry and similarity scores. Additionally, they showed higher normalized intensities at the peak boundaries, as well as poor consistency in FWHM across samples and isotopes and poor consistency in pair isotope ratios across samples.

#### Predictive peak quality model development

The training set was used for development of five models using different statistical approaches, including RRF, regularized logistic regression, SVM with linear and polynomial kernels and KNN methods. Similar to the previous example, the training set was split into training (80%) and validation (20%) subsets. The model was built using the training subset, while the validation subset was used for unbiased evaluation of the performance of the model. Using the validation subset for performance evaluation helps diagnose issues with overfitting and therefore is of great importance in choosing the final model.

Performance of the five developed models was compared in the training as well as the validation subset (Additional file 1: Table S3 and Figure S8). The RRF model yielded the highest classification accuracy, achieving an accuracy of 89.1% and 91.1% in the training and validation subsets, respectively. The accuracy of the other tested models ranged from 80.1% in the linear SVM model to 87.0% in the KNN model. However, the sensitivity of the RRF model for flagging peaks with low quality significantly outperformed that of the other four models and therefore RRF was selected as the final predictive model for peak quality assessment in this dataset. Furthermore, receiver operator characteristics (ROC) analysis of the outcome of the RRF model on the validation subset returned an area under the curve (AUC) of 0.975, which also demonstrates the high performance of the predictive model (Additional file 1: Figure S9). Depending on the difference between the distribution of individual QC features in low and high quality peak groups, each of these metrics plays a role in determining the output of the model. In this dataset, QC features that measure attributes such as peak intensity, modality, correlation and consistency of isotope peak area ratios, peak area CV%, FWHM, and symmetry appear to have a more prominent impact on the output of the model (Additional file 1: Figure S10). Alternatively, jaggedness and shift scores do not seem to play a crucial part in determining the peak quality in the RRF model.

It should be noted that applying the model developed in the artificial matrix to assess the quality of the longitudinal biomarker data and vice versa shows poor performance. This can be attributed to the complexity of the biological matrix in the experiment. The highly complex human CSF matrix containing endogenous biomarkers poses a greater challenge for the LC-MS/MS method when compared to the artificial CSF matrix, which consists of bovine serum albumin with spiked-in standard isotope-labeled peptides. The matrix effect, biological variability of samples and unpredictable interference from the endogenous analytes in CSF compromises the chromatography in the longitudinal biomarker study. This ambiguity complicates the definition of acceptable peaks in the dataset and creates a gray zone, where the assessment of the peak quality may be more subjective. In contrast, using BSA as an artificial CSF matrix creates a controlled sample, where the difference between high and low quality peaks is clearer. This clarity further simplifies annotation of the training set and subsequently optimization of the performance of the model. Moreover, the concentrations of spiked-in AQUA™ peptides in the artificial CSF samples were greater than the limit of quantitation for the vast majority of the peptides, which generates peaks with higher intensities and fewer jagged or irregular peaks. Considering that the peaks that are close to the limit of quantitation are more anomalous in shape and quality, it is expected that a dataset of high intensity peaks, as seen in the experiment with artificial matrix, leads to a more simplified quality assessment process both through manual and automated workflows.

Additional file 1: Figure S11 highlights the transitions that were misclassified by the model in the longitudinal biomarker study in red. Of the 1128 transition pairs in the training set, 7 were misclassified as ‘ok’ (S9 A–F) and 13 were misclassified as ‘flagged’ (S9 G–R) by the model. A majority of misclassified peaks fall into the gray area between a high and low quality peak. Ambiguity surrounds the annotations of flagged data by both the model and through manual inspection. To confirm this hypothesis, we can evaluate the class probabilities for the misclassified peaks. Class probability for each transition pair is the likelihood of that transition falling into a certain class as estimated by the RRF model. For example, a ‘flag’ class probability of 0.5 or higher for a transition, results in flagging of that transition by the model as a low quality peak. Transitions with class probabilities closer to 0.5 are considered marginal cases. The ‘flag’ class probability of the transitions correctly flagged by the model is 0.91 ± 0.10, whereas the probability for low quality misclassified transitions is 0.65 ± 0.13, indicating a higher level of uncertainty about the peaks that were misclassified. Similarly, the ‘ok’ class probability of the transitions that correctly passed QC by the model is 0.91 ± 0.09, while this probability for the low quality transitions that were misclassified as high quality is 0.70 ± 0.15. Many of the misclassified transitions are low intensity peaks that are either below or very close to the limit of quantitation. At such low concentrations, the quality of the peak could be compromised not due to poor chromatography or interference, but only because of the low intensity of the peak and limitations of the detector in resolving the peak. Therefore, defining more rigorous quality assessment criteria at these low levels may decrease the uncertainty of the model and therefore improve its performance.

#### Peak quality assessment in large targeted MS datasets using the predictive model

The optimized RRF model was applied to the CSF biomarker longitudinal study dataset. The QC features were calculated for each transition pair for 36 peptides quantified in 70 runs and used as the input to the model. TargetedMSQC generated a report based on the output of the model summarizing the QC results for the complete dataset and at sample and peptide levels. Additionally, for each peptide with an isotope pair, model output was visualized for individual transitions and samples. Here, two of these peptides are discussed in more details as examples of how this tool can assist the analyst in method development and data analysis. The full TargetedMSQC report for the CSF biomarker longitudinal study is provided in the Additional file 2.

**Fig. 3:**
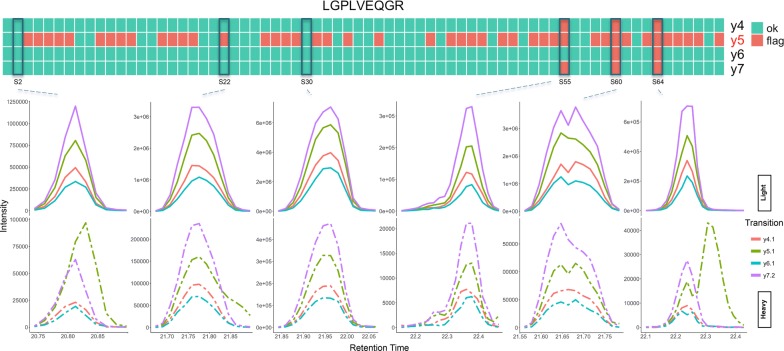
QC results summary for peptide LGPLVEQGR. The heatmap shows the model output for each transition pair and each sample. With the exception of transition y5, the peptide passes QC in almost all the samples and therefore can be accurately quantified. Transition y5 seems to be systematically flagged across many of the samples. Manual inspection of y5 reveals that this transition is affected by interference in most of the samples. The peak groups for 6 of the 70 runs in this study are depicted to compare the model output with manual evaluation of these peaks

Figure 3 shows the QC results summary for peptide LGPLVEQGR. Four transitions, y4, y5, y6 and y7, were monitored for quantification of this peptide. The heatmap in Fig. 3 shows the output of the model for individual transitions across 70 samples included in the study. The peak groups for 6 of these runs are depicted in the figure as representatives of the dataset. Sample S30 shows an example of a high quality peak, which has correctly passed QC by the model. As shown in Fig. 3, three of the transitions, y4 (red), y6 (blue) and y7 (purple) passed QC by the model in almost all of the samples. On the other hand, y5 (green) is flagged in the majority of the samples, including samples S22, S55, S60 and S64. LGPLVEQGR shows an example of a peptide that could be accurately quantified across multiple samples by removing the transition with interference (y5) from the quantitative analysis. Upon manual inspection of y5 across these samples, we observed that this transition had interference in many of the flagged samples similar to what is seen in S22 and S64. The interference resulted in high background at the peak boundary (S22: normalized intensity at boundary of 0.17 compared with 0.02 as the median of the dataset) and low similarity between the endogenous and standard pairs (S64: pair similarity score of 0.06 compared with 0.98 as the median of the dataset) and high modality score (S64: transition modality score of 0.34 compared with 0.00 as the median of the dataset). Sample S55 shows an example of a peak with poor chromatography and shoulders, where at least three of the transitions have been flagged due to a shoulder, particularly in the standard peptide. Sample S60 shows an example of a peak with bimodal transitions that has also been correctly flagged by the model. All the transition pairs in this group suffer from jaggedness, poor modality and low similarity scores, all of which are likely the main driving force behind the output for this peak. Although the model performs well in a majority of the samples, there are examples where the output is not as expected. Sample S2 shows a peak group where the y5 transition has interference and needs to be flagged; however the model failed to flag this transition as a low quality transition. It should be noted that the model has predicted a value of 0.492 as the ‘flag’ class probability for this transition, which indicates that the QC features calculated for this transition place it on the verge of being flagged. A solution to improving the sensitivity of the model for detection of marginal low quality peaks such as y5 in sample S2 is to lower the default threshold of 0.5 as the cut-off class probability for flagged peaks in the RRF model. At the expense of increasing the rate of incorrectly flagging high quality peaks, lowering the threshold could help flag more peaks and transitions in the marginal gray area between the two classes.

**Fig. 4:**
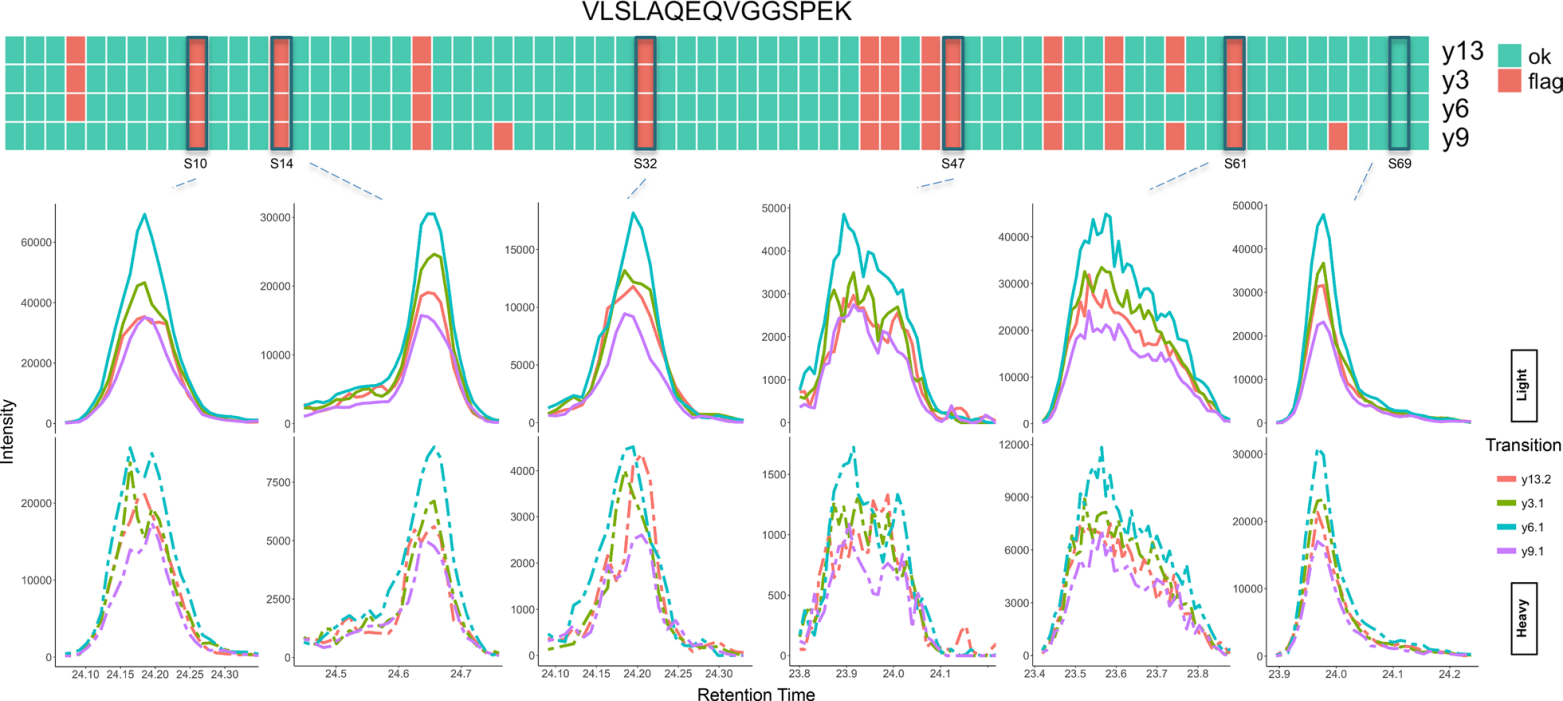
QC results summary for peptide VLSLAQEQVGGSPEK. The heatmap shows the model output for each transition and each sample. In this example, several samples suffer from poor quality across all the transitions and therefore the quantitative results in these samples may be compromised. The peak groups for 6 of the 70 runs in this study are depicted to compare the model output with manual evaluation of these peaks

Figure 4 shows the QC results summary for peptide VLSLAQEQVGGSPEK. Unlike the LGPLVEQGR example, none of the transitions have been systematically flagged across majority of the samples. However, all four monitored transitions seem to be flagged in a number of samples. One could conclude from this data that in these samples the transitions cannot be used to reliably quantify the target peptides. Many of the flagged peaks seem to have failed quality assessment due to low intensity, jagged and tailing peaks. This is particularly evident for the heavy standard isotopes. This is represented in samples S10 and S32. Both have slightly jagged, bimodal and low intensity standard signals. Samples S47 and S61 are highly jagged, slightly tailing and have very low standard signals. Sample S14 is jagged and tailing and therefore flagged for poor quality. Finally, sample S69 shows an example of a peak that has correctly passed QC. It should be noted that although this peak is tailing, the peak quality is otherwise acceptable and therefore similar peaks in the training set were marked as acceptable.

## Discussion and conclusion

In this study, a framework and toolset for quality assessment of large proteomics targeted MS datasets has been presented. This framework entails building and applying a predictive QC model tailored to the proteomics panel as well as the matrix used in the study to flag low quality peaks and transitions. The predictive model is optimized based on a subset of pre-annotated peaks from the dataset, which provides guidelines on what represents an acceptable peak. A step-by-step guide for the QC process using TargetedMSQC in a laboratory setting has been provided as a vignette in the R package. This framework can benefit the study in several ways. First, using the same model for peak quality assessment of the whole dataset ensures that quality standards are not altered during the course of the study. Given the inter- and intra-operator variability of manual peak assessment, the prolonged duration and multi-center nature of many proteomics studies, this model provides a unified way to standardize data QC and perform more robust analysis. Second, an automated QC approach reduces the time spent by the analyst on manual inspection of every peak. This algorithm allows the analyst to focus on peaks that have been flagged by the model. For instance, applying the predictive model for QC of our dataset of 70 samples, 36 peptides, and 144 transitions, the process took less than 30 min, whereas manual inspection would have lasted days. It should be noted that an analyst should still review and adjust the boundaries on peaks that have been flagged by TargetedMSQC. However, this manual step is greatly reduced increasing the overall efficiency of the QC process. The TargetedMSQC framework is designed to be generic and not limited by instrument or platform and the QC process and standards can be optimized for each individual dataset. Providing such an agnostic framework allows for use in the quality assessment of large datasets and for the optimization of method development, by flagging transitions or peptides that might be poor performing or have low reproducibility. Finally, as demonstrated in this study, the rate of low quality peaks that were missed by the model is low, which speaks to the sensitivity of the developed models for identification of interference, poor chromatography and other peak quality issues. The problem at hand, which is automated flagging of peaks with poor quality, has a higher tolerance for false positives than false negatives. In other words, mistakenly flagging high quality peaks imposes a lower cost than mistakenly passing a low quality peak. Therefore, it is advisable to prioritize the sensitivity of the model over its specificity during model optimization and selection. In the representative examples, the peaks that were incorrectly marked as ‘ok’ were usually marginal cases where the imperfection in the peak quality did not significantly impact the outcome of the quantitative results. Moreover, if the data contains significantly fewer ‘flagged’ peaks, accuracy may be a misleading performance metric for selecting the best model. For training a model based on imbalanced data, it is recommended to use ‘ROC’ as performance metric. TargetedMSQC allows the users to choose between accuracy and ROC for model selection.

The proposed framework provides several benefits for streamlining the QC process in targeted MS workflows. It should be noted that this framework does have some limitation. One of the most time-consuming steps of building a quality assessment model using TargetedMSQC is in fact building a training dataset to guide model development. Currently, annotation of the training set is conducted manually and takes a few hours to complete. Although this may be time-consuming for a small experiment, for a large datasets that require days of analysis time, a few hours spent on creating a training set can be considered acceptable. Additionally, integrating this framework into software tools such as Skyline, could simplify generation of the training set by enabling the user to simply click a peak to flag it in the training set. Annotation of the training set can be accelerated by automating some of the annotation steps. Peaks with high background can be identified by higher intensities at the boundary. This value is included as one of the QC features calculated by TargetedMSQC and automated flagging of all peaks with high background as defined by this metric could simplify the annotation step. Another limitation of the TargetedMSQC approach is that, similar to any supervised learning method, the quality of the model depends on the quality of the training set. In other words, the model still inherits the subjectivity or bias of the individual who annotates the training set. The effect of such bias is usually more pronounced when the training dataset is small. To overcome these biases, training sets should always be reviewed by multiple analysts. This would be particularly beneficial for multi-center studies. Additionally, increasing the size of the training set could further mediate the impact of bias in the annotation step.

It should be noted that annotation and classification of transitions that do not clearly belong to either of the high and low quality classes is a challenging task. This is due to the fact that the quality of each peak is a continuous variable, not a binary one. The RRF model assigns ‘flag’ and ‘ok’ class probabilities between 0 and 1 to each transition. For visibly high or low quality peaks, the flag class probability is close to 0 and 1, respectively. However for marginal cases, the class probability falls closer to the midpoint of 0.5. By default, the model uses a cut-off threshold of 0.5 to assign a class to each transition according to its class probabilities. As shown in the example in Fig. 3, this may result in misclassification of marginal cases that fall very close to the cut-off point. For this reason, it would be beneficial for the users to be able to manually adjust the cut-off threshold. To help the users decide on an acceptable threshold, ROC analysis can be performed to estimate the sensitivity and specificity of the model to identify low quality peaks in the training set at each cut-off value (Additional file 1: Figure S9). This helps the user choose a cut-off that tunes the rates of false positives and false negatives according to their tolerance for each group. It should be noted that since the ROC analysis does not cross-validate the estimated sensitivity and specificity, these values should be taken into account with caution. To estimate confidence intervals for sensitivity and specificity, users may choose ROC as performance metric for training the model. Another way to address the challenges of classifying marginal peaks is including more examples of such cases in the training set, which may enable the model to better resolve low and high quality peaks. This could be achieved by first applying the model built on the initial training set to the dataset, identifying marginal cases based on their class probabilities, and prompting the analyst to further annotate these cases. The extended training set then may be used to further adjust the model. Additionally, since the process of annotating the training set is manual and therefore prone to human error, evaluating the output of the initial model may enable identification and correction of peaks that have been incorrectly annotated and therefore improve the quality of the training set and subsequently, the performance of the model. It should be noted that the goal of this step is to improve the quality and diversity of the training set by informing the user of the model output. This should not be mistaken as a step to enforce the manual annotations to match with the model output, as this may result in overfitting.

Given the increasing interest in targeted MS analysis for identification, validation and quantitation of clinical biomarkers, improving and providing tools for reliable analysis of targeted MS data is both a challenge and an opportunity. Methods to standardize and streamline this process are highly needed. TargetedMSQC provides a QC framework that can be customized for specific panels, instruments and sample types in an automated, time-efficient, and reproducible manner and therefore is a step towards more robust targeted MS data analysis.

## Additional files

**Additional file 1.** Supplementary materials for development of predictive QC models using TargetedMSQC.

**Additional file 2.** TargetedMSQC report for the CSF biomarker longitudinal study.
