# Supplementary material for: Quality assessment and interference detection in targeted mass spectrometry data using machine learning
Source: Clin Proteomics. 2018 Oct 6;15:33. doi: 10.1186/s12014-018-9209-x (PMC6173846; doi:10.1186/s12014-018-9209-x)
Supplement: Supplementary file 1 — Additional file 1. Supplementary materials for development of predictive QC models using TargetedMSQC. [file 12014_2018_9209_MOESM1_ESM.pdf]

Supplementary Material for

Quality Assessment and Interference Detection in Targeted Mass Spectrometry Data using Machine Learning

Shadi Toghi Eshghi, Paul Auger, W. Rodney Mathews
OMNI-Biomarker Development, Genentech Inc., South San Francisco, California 94080, United States

Table S1. Description of QC features in TargetedMSQC. The engineered QC metrics can be classified in nine general categories indicated in the 'QC Group' column. Depending on what attribute of the peak quality they represent, various QC features are calculated at one or more levels as shown in the 'Level' column. For example, jaggedness is reported at transition, isotope and peak group levels, and max intensity is reported only for each transition. The 'Description' column provides a definition for each of the QC features.

QC Group	QC Metric Name	Level	Description
Area Ratio	Area2SumRatioCV	Transition	CV of the transition AUC to sum of transition AUCs across all samples for each transition.
Area Ratio	PeakGroupRatioCorr	Peak group	Correlation coefficient of transition AUC to sum of transition AUCs between endogenous and standard isotopes for each peak group for each sample.
Area Ratio	PairRatioConsistency	Transition pair	Consistency of the transition AUC to sum of transition AUCs between endogenous and standard isotopes for each transition pair for each sample
Area Ratio	MeanIsotopeRatioConsistency	Transition	Consistency of the transition AUC to sum of transition AUCs of the same isotope label across all samples for each transition for each sample.
Jaggedness	TransitionJaggedness	Transition	Jaggedness score for each transition for each sample. Jaggedness score is defined as the fraction of time points across a peak where the signal changes direction, excluding the peak apex.
Jaggedness	IsotopeJaggedness	Isotope	Mean of jaggedness scores of peaks with similar isotope label in a peak group for each sample.
Jaggedness	PeakGroupJaggedness.m	Peak group	Mean of jaggedness scores of all peaks in a peak group for each sample.
Symmetry	TransitionSymmetry	Transition	Symmetry score for each transition for each sample. Symmetry score is defined as the Pearson correlation coefficient between left and right half of the peak intensities.
Symmetry	IsotopeSymmetry	Isotope	Mean of symmetry scores of peaks with similar isotope label in a peak group for each sample.
Symmetry	PeakGroupSymmetry.m	Peak group	Mean of symmetry scores of all peaks in a peak group for each

			sample.
Similarity	PairSimilarity	Transition pair	Similarity score for each transition pair for each sample. Pair similarity score is defined as the Pearson correlation coefficient between peak intensities of two peaks.
Similarity	IsotopeSimilarity	Isotope	Mean of similarity scores of peak pairs with similar isotope label in a peak group for each sample.
Similarity	PeakGroupSimilarity.m	Peak group	Mean of similarity scores of all peak pairs in a peak group for each sample.
Modality	TransitionModality	Transition	Modality score for each transition for each sample. Modality score is defined as the largest unexpected dip in the peak, normalized by peak height.
Modality	IsotopeModality	Isotope	Mean of modality scores of peaks with similar isotope label in a peak group for each sample.
Modality	PeakGroupModality.m	Peak group	Mean of modality scores of all peaks in a peak group for each sample.
Shift	TransitionShift	Transition	Elution shift score for each transition for each sample. Elution shift score is defined as the difference between time at max intensity of each peak and the median of time at max intensities of all the peaks in the peak group normalized by the peak base width.
Shift	PairShift	Transition pair	Elution pair shift score for each transition pair for each sample. Shift score between two peaks is defined as the difference between the time at max intensities of the two peaks normalized by the peak base width.
Shift	IsotopeShift	Isotope	Mean of transition shift scores of peaks with similar isotope label in a peak group for each sample.
Shift	PeakGroupShift	Peak group	Mean of transition shift scores of all peaks in a peak group for each sample.
FWHM	TransitionFWHM	Transition	FWHM for each transition for each sample. (FWHM: full-width at half-max)
FWHM	TransitionFWHM2base	Transition	FWHM to peak base width ratio for each transition for each sample.

FWHM	IsotopeFWHM	Isotope	Mean of FWHM of peaks with similar isotope label in a peak group for each sample.
FWHM	IsotopeFWHM2base	Isotope	Mean of FWHM to peak base width ratios of peaks with similar isotope label in a peak group for each sample.
FWHM	PeakGroupFWHM.m	Peak group	Mean of FWHM of all peaks in a peak group for each sample.
FWHM	PeakGroupFWHM2base.m	Peak group	Mean of FWHM to peak base width ratios of all peaks in a peak group for each sample.
FWHM	PairFWHMConsistency	Transition pair	Consistency of the FWHM between endogenous and standard isotopes for each transition pair for each sample:
FWHM	MeanIsotopeFWHMConsistency	Transition	Consistency of the FWHM of the same isotope label across all samples for each transition for each sample.
Retention Time	MeanIsotopeRTConsistency	Transition	Consistency of the peak retention time of the same isotope label across all samples for each transition for each sample.
Intensity	TransitionMaxIntensity	Transition	Maximum peak intensity of each transition for each sample.
Intensity	TransitionMaxBoundaryIntensity	Transition	Maximum peak intensity at the peak boundary of each transition for each sample.
Intensity	TransitionMaxBoundaryIntensityNormalized	Transition	Maximum peak intensity at the peak boundary normalized by maximum peak intensity of each transition for each sample.

Figure S1. Learning curve for the CSF biomarker in artificial matrix dataset. Cross validation and resampling was used to estimate the performance of the RRF model as a function of the training set size. This curve is used to determine the minimum required size for the training set to achieve acceptable performance. Here, to achieve classification accuracy of 90%, the training set should contain at least 400 transition pairs.

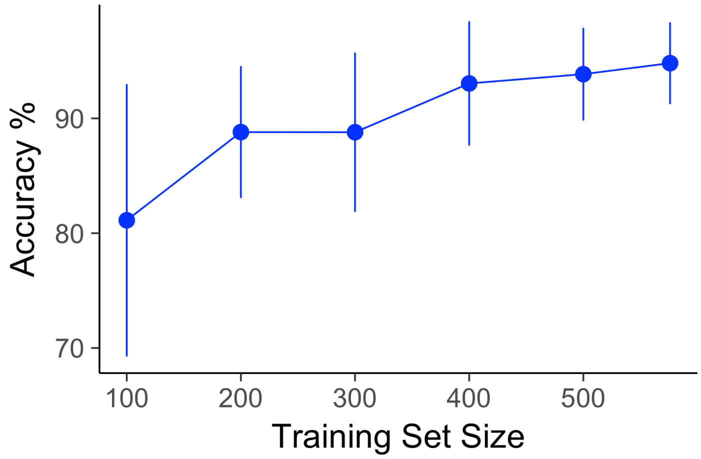

Figure S2. Distribution of QC features in the training dataset for the CSF biomarker in artificial matrix dataset. The violin plots illustrate the distribution of peak quality metrics in a training dataset of 576 transition pairs in artificial CSF matrix. Red and green dots indicate transitions that were marked as 'flag' and 'ok' by manual inspection, respectively.

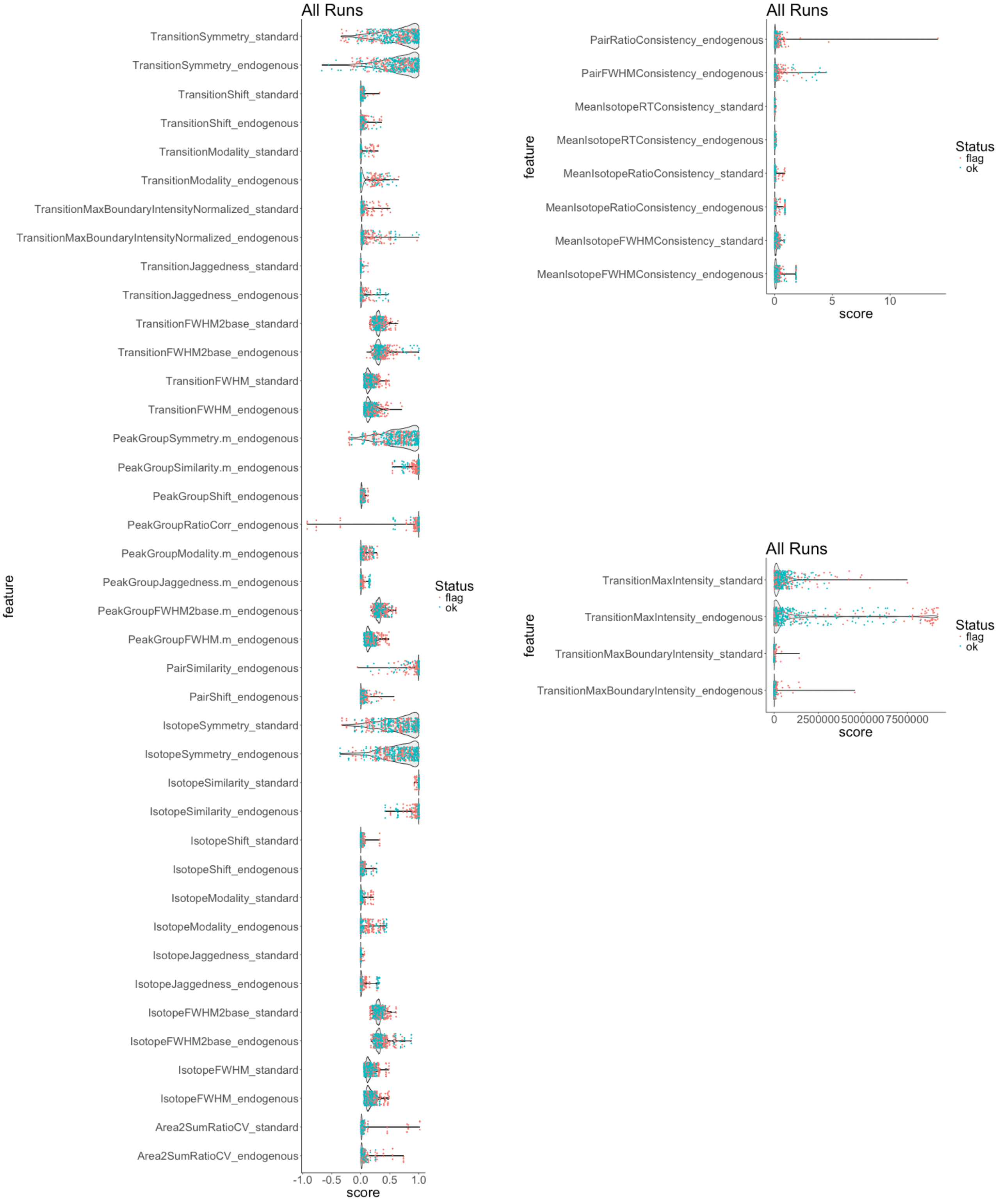

Table S2. Summary of performance of peak quality models for the CSF biomarker in artificial matrix dataset. Five supervised machine learning methods were tested for building a predictive peak quality model. The accuracy of the cross-validated model on the training subset (random selection of 80% of the training set) and the corresponding standard deviation is shown in the 'Accuracy' and 'Accuracy Standard Deviation' columns. The models were further tested on the validation subset (the remaining 20% of the training set) that was excluded from the training process. The results of this evaluation are shown in 'Accuracy', 'Sensitivity' and 'Specificity on Validation Subset' columns, respectively. The performance of the regularized random forest model surpassed that of the others.

Model	Model Description	Accuracy % (cross validation)	Accuracy Standard Deviation % (cross validation)	Accuracy % on Validation Subset	Sensitivity % on Validation Subset	Specificity % on Validation Subset
RRF	Regularized Random Forest	94.5	3.6	98.2	97.4	98.7
KNN	K-Nearest Neighbor	93.4	3.3	93.0	82.1	98.7
SVM.Polynomial	Support Vector Machine with Polynomial Kernel	93.2	4.4	95.6	92.3	97.3
Logistic.Regression	Regularized Logistic Regression	90.4	4.3	90.4	79.5	96.0
SVM.Linear	Support Vector Machine with Linear Kernel	89.8	3.8	90.4	82.1	94.7

Figure S3. Comparing the accuracy of peak quality models for the CSF biomarker in artificial matrix study. Accuracy of each model in predicting peak quality was estimated and compared through collecting resampling results. Error bars indicate 95% confidence intervals of the accuracy. The performance of the RRF model surpasses that of the others.

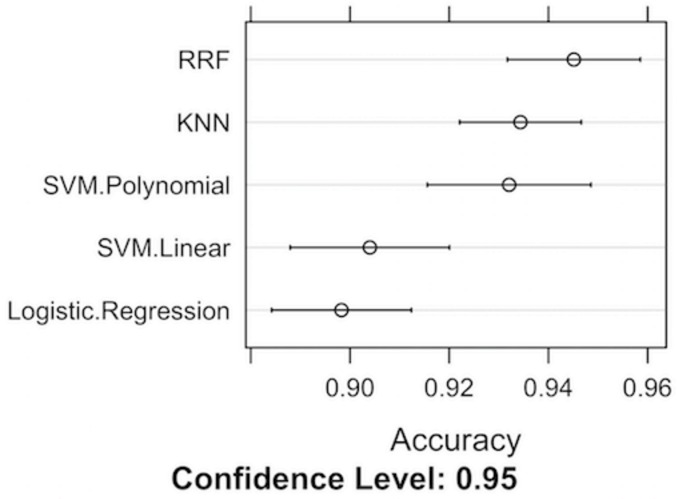

Figure S4. Relative importance of QC features in determining the output of the predictive model for the CSF biomarker in artificial matrix dataset. Every defined feature represents certain aspects of peak quality; however, some features play a more defining role in determining the output of the model. In this example, features that are related to FWHM, intensity, peak similarity and correlation and consistency of isotope pair ratios are relatively more important in the outcome of this model. In contrast, features that are related to jaggedness and shift seem to have less influence on the output.

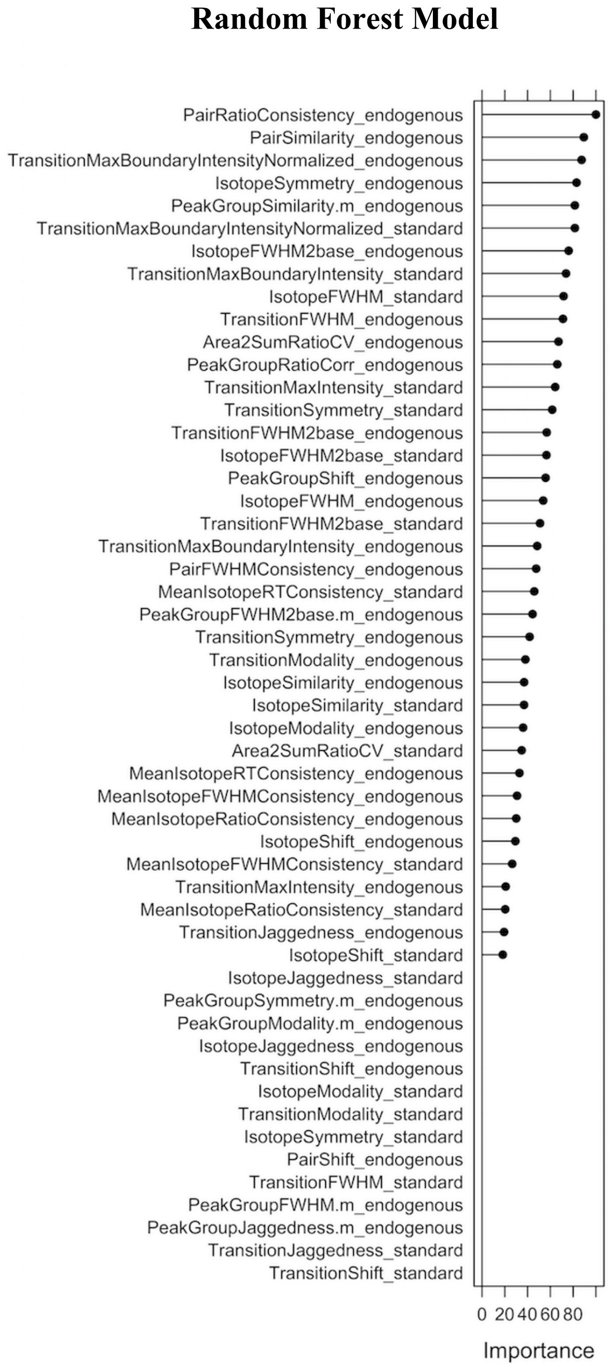

Figure S5. Examples of misclassification by the predictive peak quality model for the CSF biomarker in artificial matrix dataset. The outcome of the predictive model was in disagreement with the manual annotation of the training dataset in only two cases shown here. Transitions y8 (A), which was flagged in the training set due to high background at the peak boundary, was marked as 'ok' by the model, resulting in a false negative outcome. In contrast, transition y3 (B) that had passed QC through manual inspection was flagged by the model, resulting in a false positive. It should be noted that both of these examples are marginal cases that do not considerably impact the quantitative results and the outcome of the experiment.

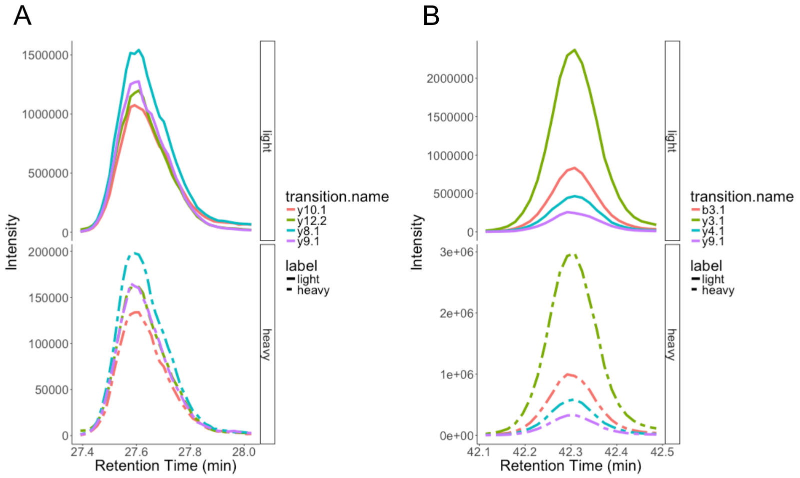

y8 (manual: flag, model: ok)	b3 (manual: ok , model: ok)
y9 (manual: ok , model: ok)	y3 (manual: ok , model: flag)
y10 (manual: flag, model: flag)	y4 (manual: ok , model: ok)
y12 (manual: ok , model: ok)	y9 (manual: ok , model: ok)

Figure S6. Learning curve for the longitudinal CSF biomarkers of AD dataset. Cross validation and resampling was used to estimate the performance of the RRF model as a function of the training set size. This curve is used to determine the minimum required size for the training set to achieve acceptable performance. Here, the curve plateaus at ~ 900 transition pairs in the training set. Therefore, increasing the training set size beyond that may not provide much more benefit.

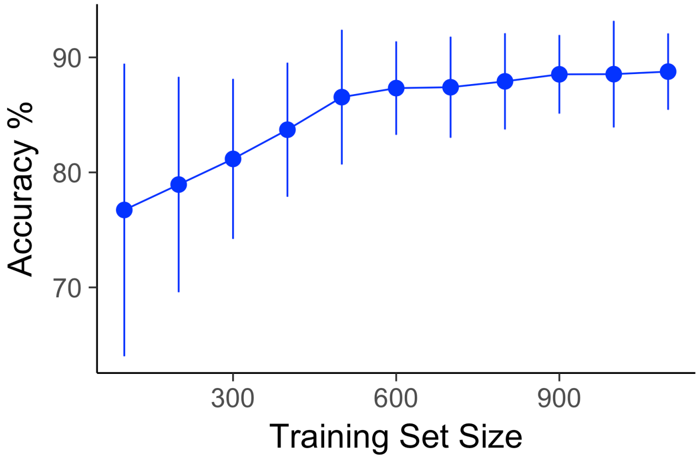

Figure S7. Distribution of QC features in the training dataset for the longitudinal CSF biomarkers of AD dataset. The violin plots illustrate the distribution of peak quality metrics in a training dataset of 1128 transition pairs measured in CSF matrix. Red and green dots indicate transitions that were marked as 'flag' and 'ok' by manual inspection, respectively.

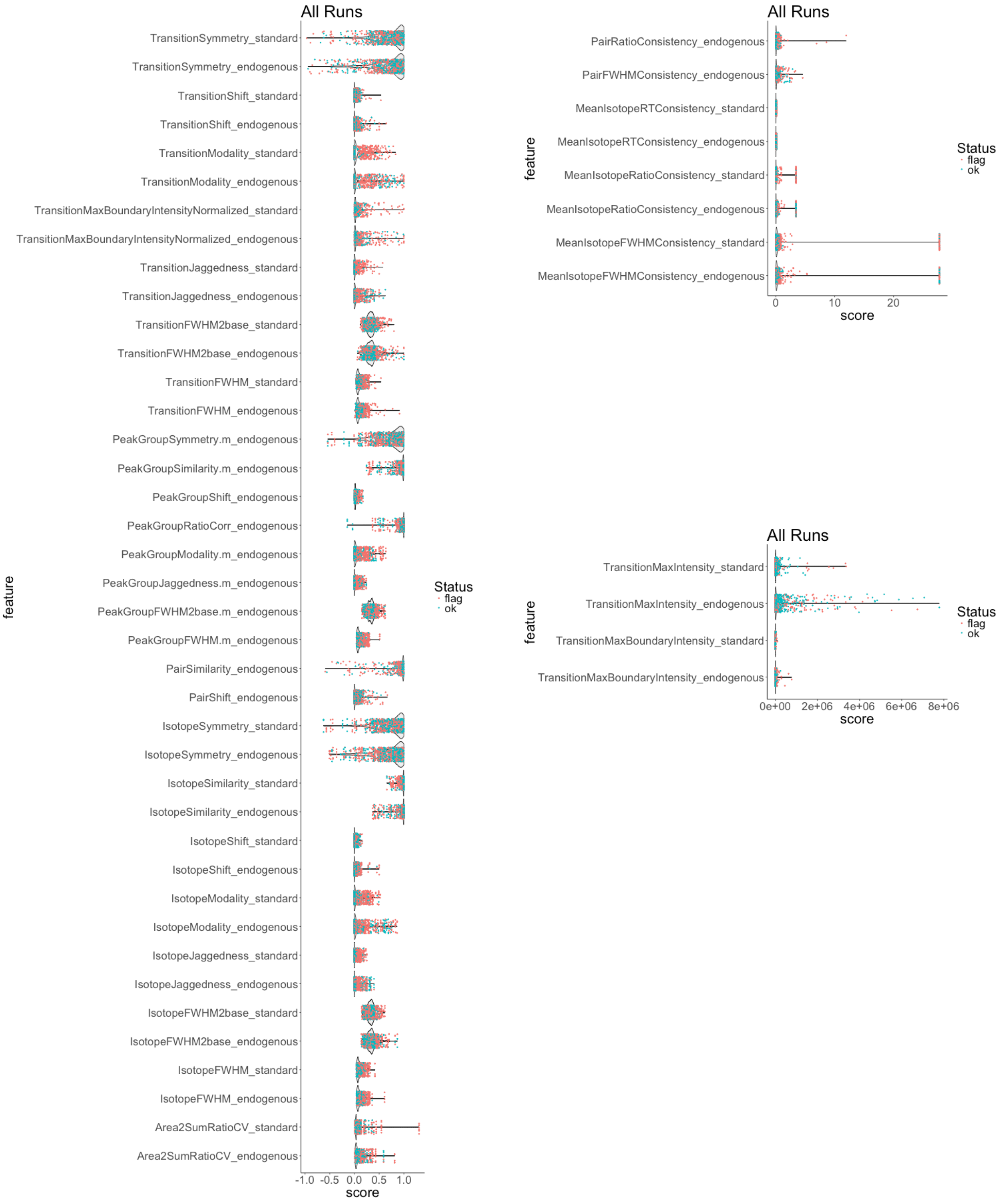

Table S3. Summary of performance of peak quality models for the longitudinal CSF biomarkers of AD dataset. Five supervised machine learning methods were tested for building a predictive peak quality model. The accuracy of the cross-validated model on the training subset and the corresponding standard deviation is shown in the 'Accuracy' and 'Accuracy Standard Deviation' columns. The models were further tested on the validation subset that was excluded from the training process. The results of this evaluation are shown in 'Accuracy', 'Sensitivity' and 'Specificity on Validation Subset columns, respectively. The performance of the regularized random forest model surpassed that of the others.

Model	Model Description	Accuracy % (cross validation)	Accuracy Standard Deviation % (cross validation)	Accuracy % on Validation Subset	Sensitivity % on Validation Subset	Specificity % on Validation Subset
RRF	Regularized Random Forest	89.1	3.5	91.1	93.1	89.4
KNN	K-Nearest Neighbor	87.0	3.1	87.6	84.3	90.2
SVM.Polynomial	Support Vector Machine with Polynomial Kernel	84.6	3.8	86.7	78.4	93.5
Logistic.Regression	Regularized Logistic Regression	80.3	3.7	81.8	76.5	86.2
SVM.Linear	Support Vector Machine with Linear Kernel	80.1	3.5	78.7	73.5	82.9

Figure S8. Comparison of accuracy of peak quality models for the longitudinal CSF biomarkers of AD dataset. Accuracy of each model in predicting peak quality was estimated and compared through collecting resampling results. Error bars indicate 95% confidence intervals of the accuracy. The performance of the RRF model surpasses that of the others.

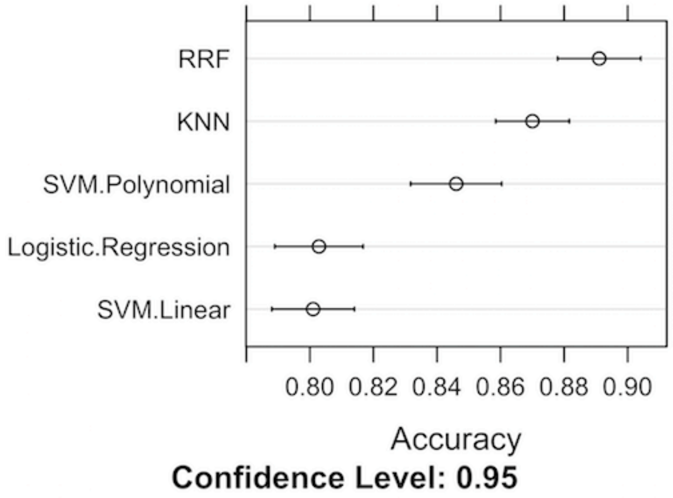

ROC analysis for the longitudinal CSF biomarkers of AD dataset

One question to address is whether the engineered QC features in this study provide an advantage over using the raw chromatograms as inputs to the model. To answer this question, we compared the outputs of two RRF models built using identical training datasets, but different sets of features. The first model took the engineered QC features as input, whereas the second model used the dimensionality reduced raw chromatograms as features to classify peaks into high and low quality groups. The features for the second model were calculated by applying principal component analysis to resampled peak intensities of the raw chromatograms in the training set. The first 5 principal components of the endogenous and standard isotopes, which captured about 99.7% of the data variability, were used as input features for building the RRF model. The performance of these two models was compared using ROC analysis. The model based on engineered QC features achieved an AUC of 0.975; a considerable improvement over the AUC of 0.876 achieved by the model based on principal components of raw chromatograms. This analysis shows that using the engineered QC features, instead of merely relying on raw chromatograms, substantially improves the ability of the model to differentiate low quality peaks.

Figure S9. ROC analysis for the longitudinal CSF biomarkers of AD dataset. ROC analysis was performed for the final RRF model developed for predicting peak qualities of candidate biomarkers in CSF matrix. The ROC curve was generated from the model output on the validation subset, returning an AUC of 0.975. Comparing the AUCs of the ROC curves between the models developed based on the engineered QC metrics (AUC of 0.975) and the principal components of peak intensities (AUC of 0.876) shows that using the engineered QC metrics in this study for distinguishing high and low quality peaks provides considerable advantage over relying on peak intensities alone.

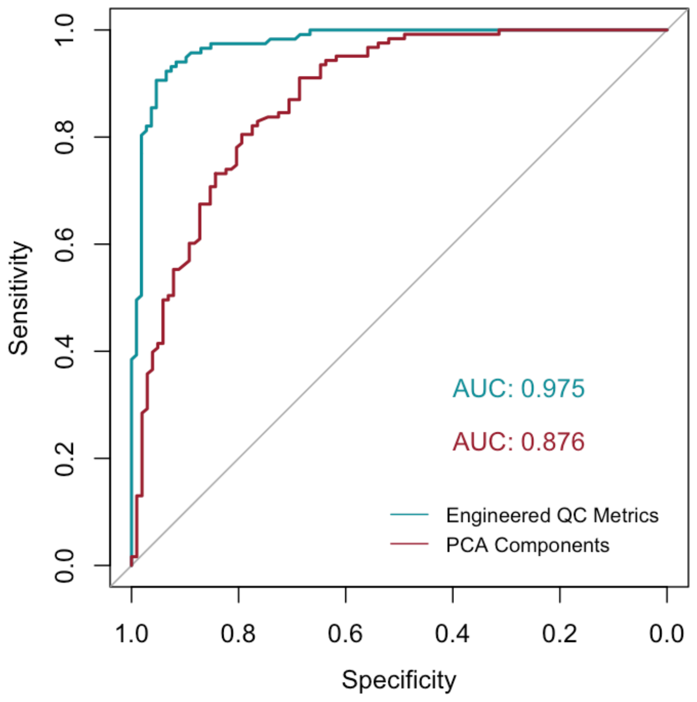

Figure S10. Relative importance of QC features in determining the output of the predictive model for the longitudinal CSF biomarkers of AD dataset. For this dataset, features that quantify peak intensity, modality, correlation of transition ratios, peak area CV and FWHM play a relatively more important role in determining the output of the model. In contrast, features that are related to jaggedness and shift seem to be of less importance.

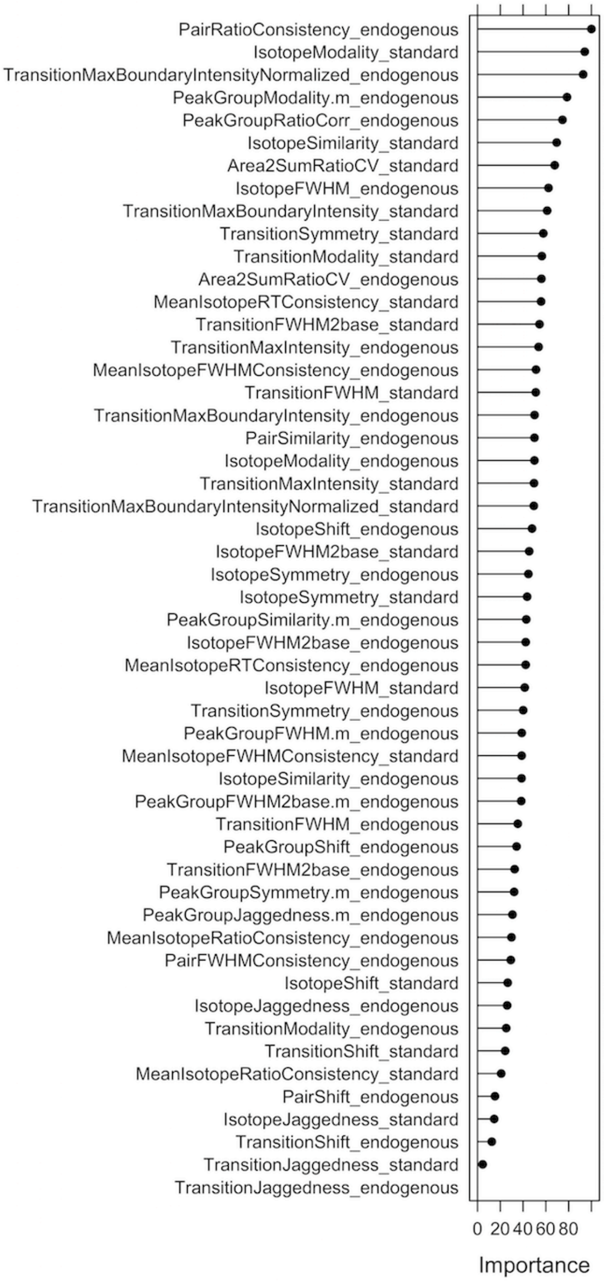

Figure S11. Examples of misclassification by the predictive peak quality model for the longitudinal CSF biomarkers of AD dataset. The outcome of the predictive model was in disagreement with the manual annotation of the training dataset for the transitions highlighted in red. Many of the misclassified transitions are below or close to the limit of quantitation and therefore difficult to annotate even through manual inspection.

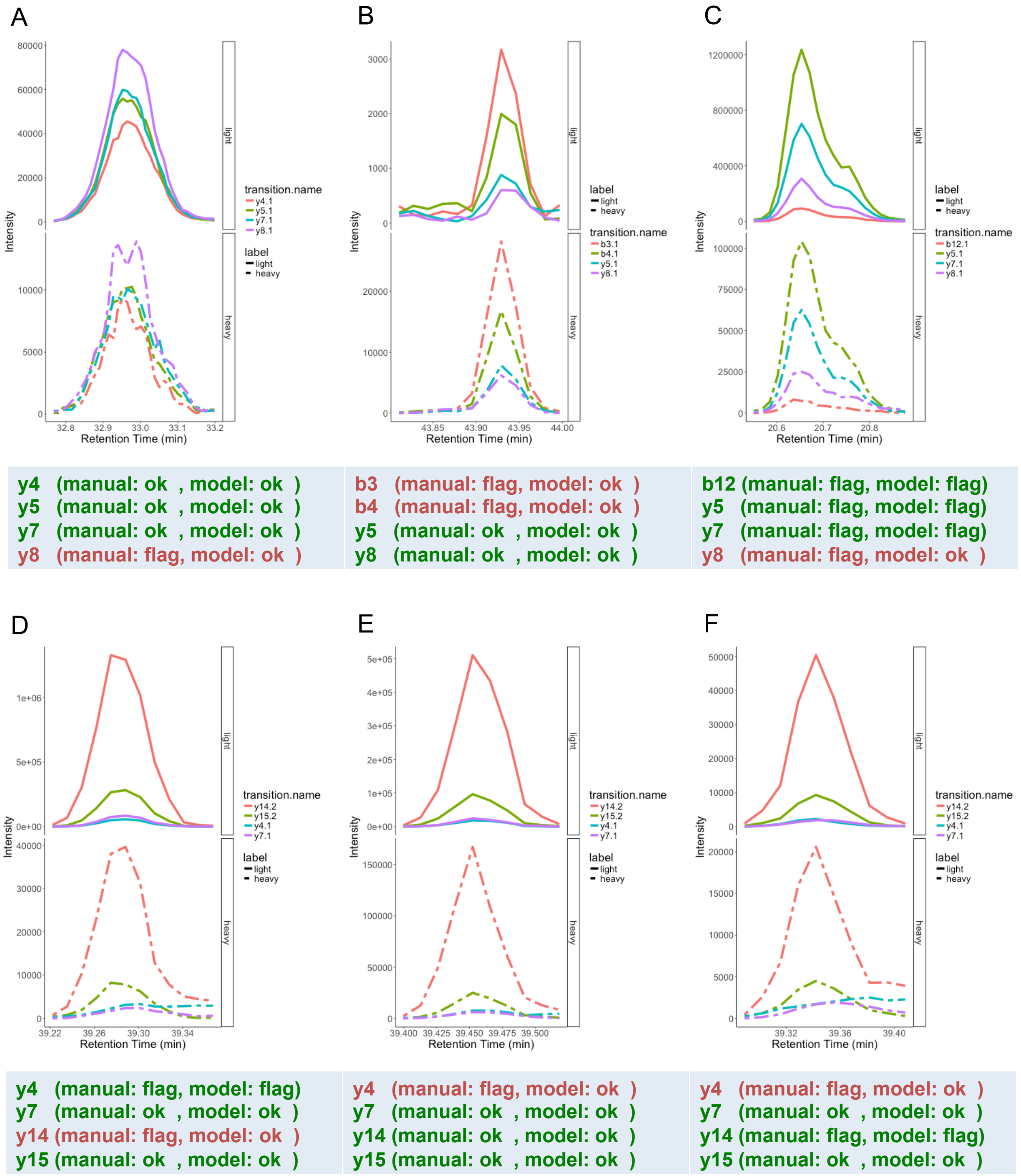

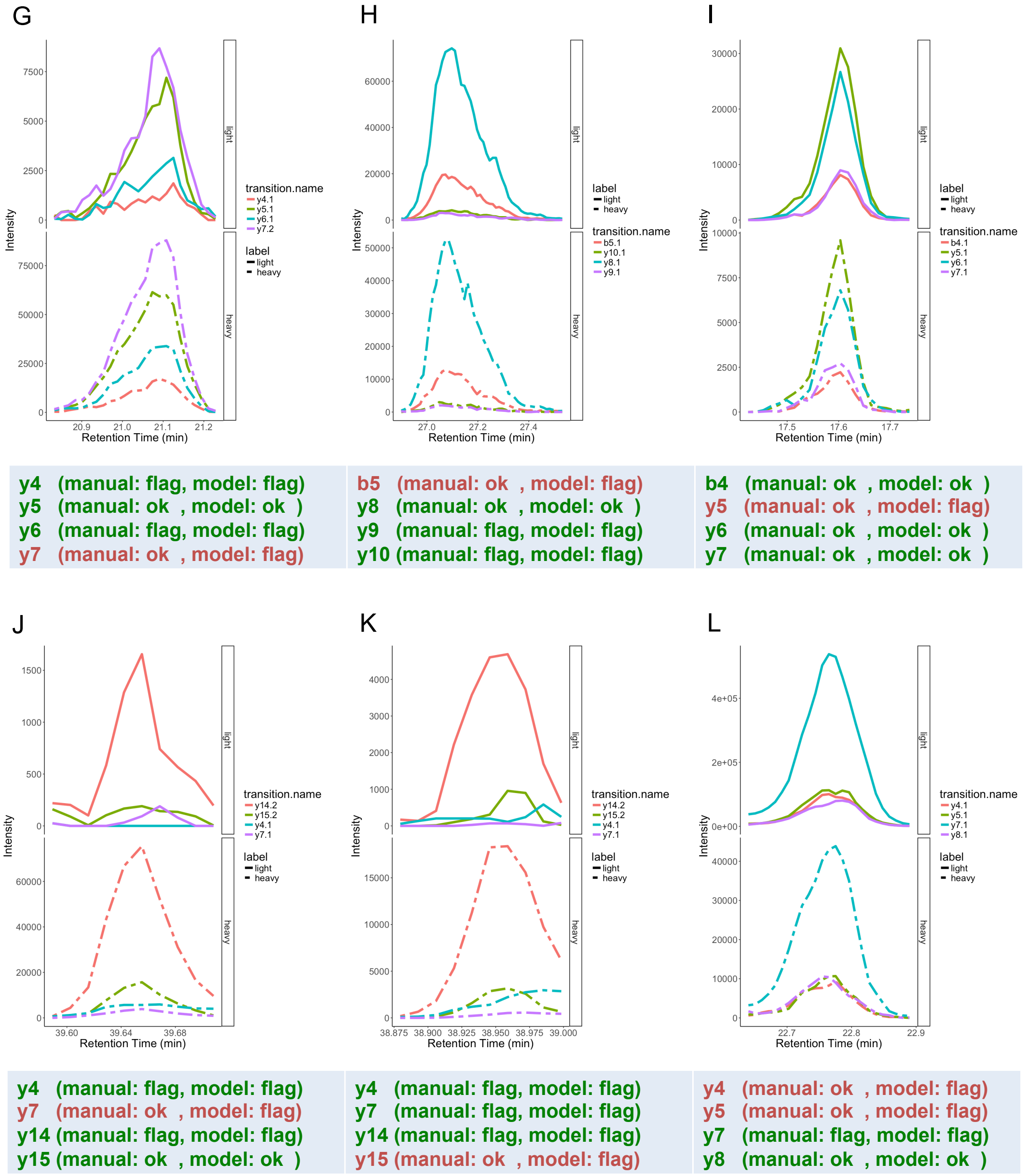
G
H
I
Intensity
Retention Time (min)
light
heavy
transition.name
y4.1
y5.1
y6.1
y7.2
label
light
heavy
b5.1
y10.1
y8.1
y9.1
b4.1
y5.1
y6.1
y7.1
y4 (manual: flag, model: flag)
y5 (manual: ok , model: ok)
y6 (manual: flag, model: flag)
y7 (manual: ok , model: flag)
b5 (manual: ok , model: flag)
y8 (manual: ok , model: ok)
y9 (manual: flag, model: flag)
y10 (manual: flag, model: flag)
b4 (manual: ok , model: ok)
y5 (manual: ok , model: flag)
y6 (manual: ok , model: ok)
y7 (manual: ok , model: ok)
J
K
L
y14.2
y15.2
y4.1
y7.1
y4.1
y5.1
y7.1
y8.1
y4 (manual: flag, model: flag)
y7 (manual: ok , model: flag)
y14 (manual: flag, model: flag)
y15 (manual: ok , model: ok)
y4 (manual: flag, model: flag)
y7 (manual: flag, model: flag)
y14 (manual: flag, model: flag)
y15 (manual: ok , model: flag)
y4 (manual: ok , model: flag)
y5 (manual: ok , model: flag)
y7 (manual: flag, model: flag)
y8 (manual: ok , model: ok)

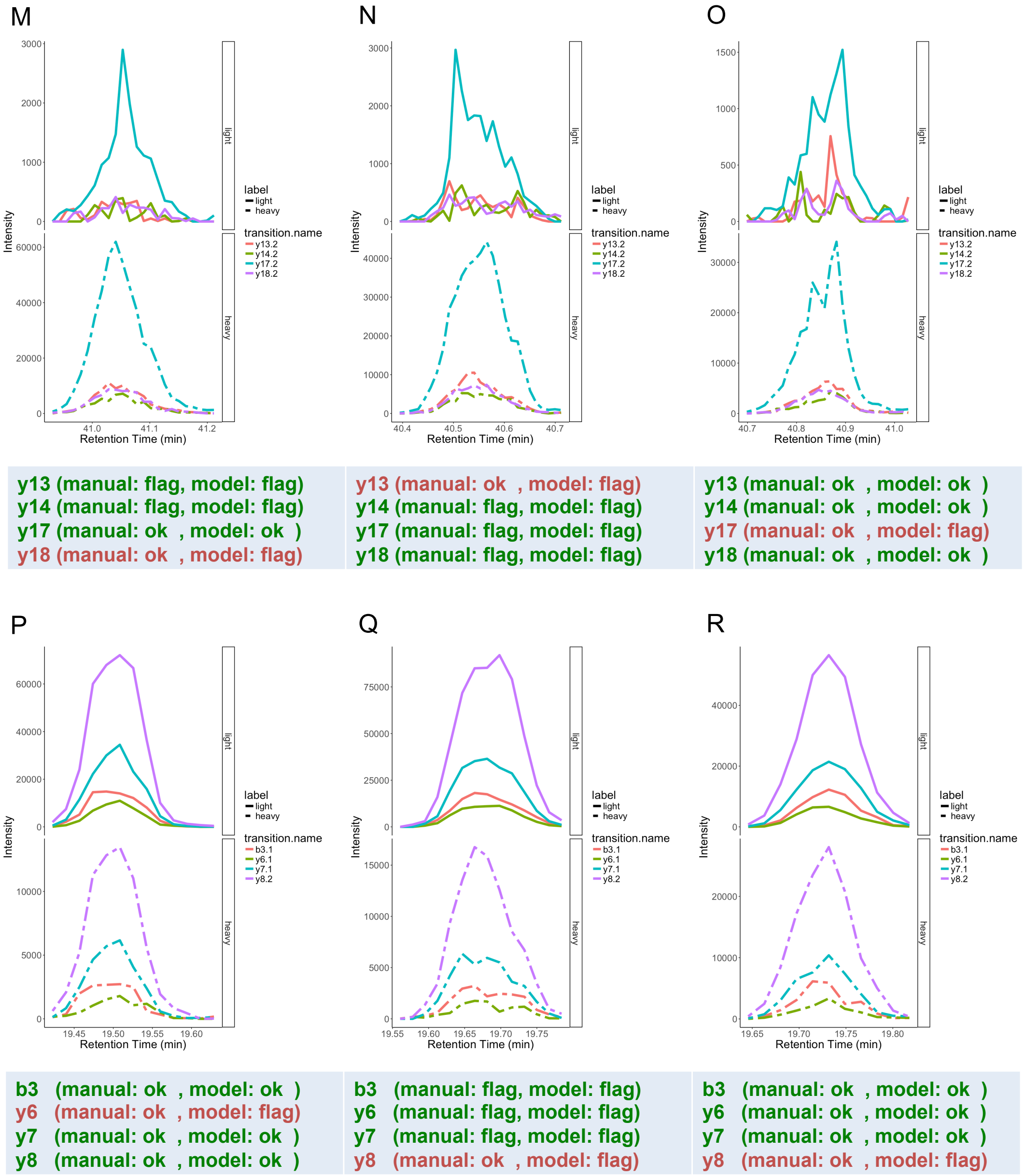

M
N
O
Intensity
Retention Time (min)
light
heavy
label
light
heavy
transition.name
y13.2
y14.2
y17.2
y18.2
y13 (manual: flag, model: flag)
y14 (manual: flag, model: flag)
y17 (manual: ok , model: ok)
y18 (manual: ok , model: flag)
y13 (manual: ok , model: flag)
y14 (manual: flag, model: flag)
y17 (manual: flag, model: flag)
y18 (manual: flag, model: flag)
y13 (manual: ok , model: ok)
y14 (manual: ok , model: ok)
y17 (manual: ok , model: flag)
y18 (manual: ok , model: ok)
P
Q
R
transition.name
b3.1
y6.1
y7.1
y8.2
b3 (manual: ok , model: ok)
y6 (manual: ok , model: flag)
y7 (manual: ok , model: ok)
y8 (manual: ok , model: ok)
b3 (manual: flag, model: flag)
y6 (manual: flag, model: flag)
y7 (manual: flag, model: flag)
y8 (manual: ok , model: flag)
b3 (manual: ok , model: ok)
y6 (manual: ok , model: ok)
y7 (manual: ok , model: ok)
y8 (manual: ok , model: flag)
