# Supplementary material for: Quality assessment and interference detection in targeted mass spectrometry data using machine learning
Source: Clin Proteomics. 2018 Oct 6;15:33. doi: 10.1186/s12014-018-9209-x (PMC6173846; doi:10.1186/s12014-018-9209-x)
Supplement: Supplementary file 2 — Additional file 2. TargetedMSQC report for the CSF biomarker longitudinal study. [file 12014_2018_9209_MOESM2_ESM.pdf]

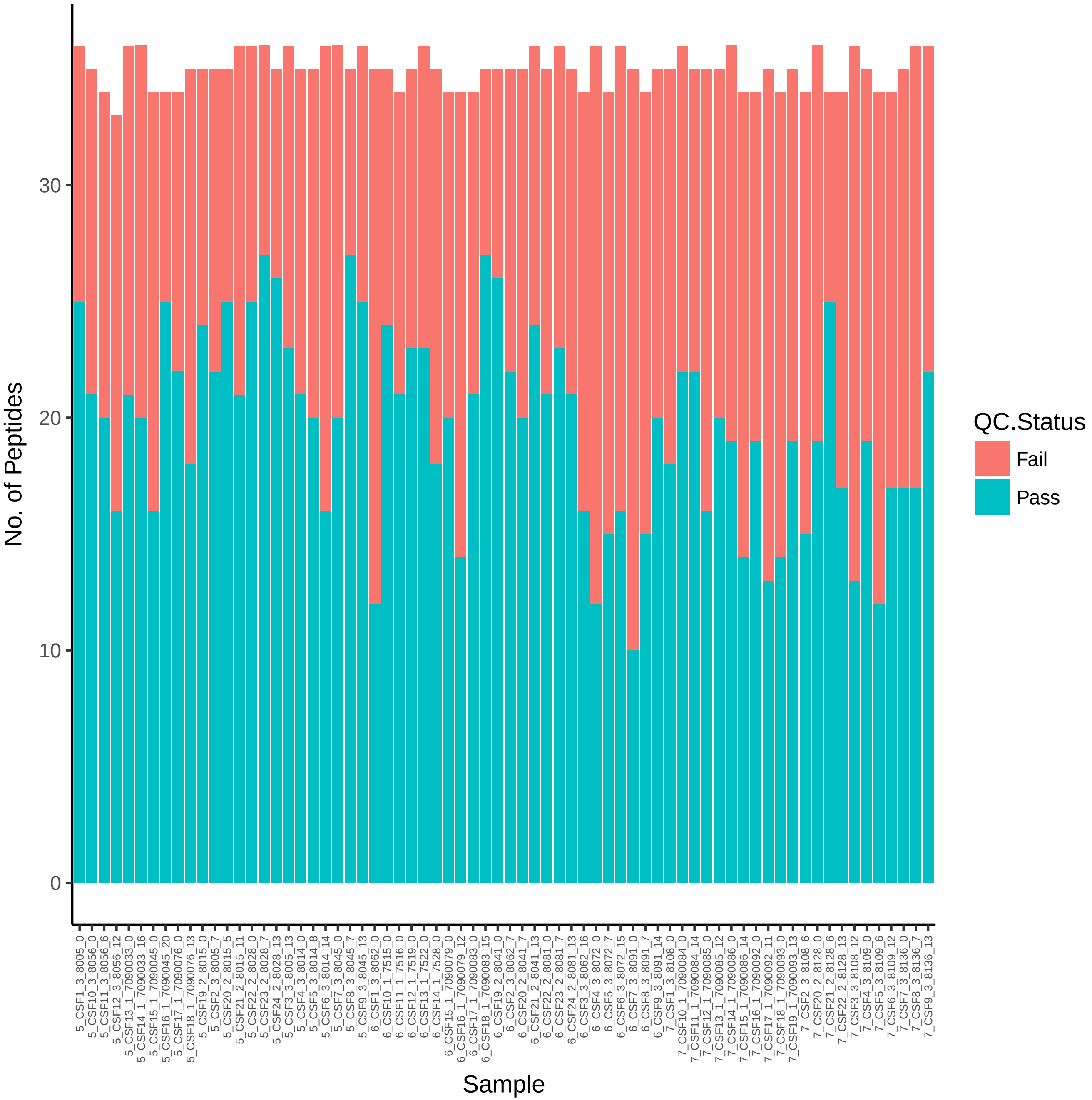

No. of Peptides
0
10
20
30
Sample
QC.Status
Fail
Pass
5_CSF1_3_8005_0
5_CSF10_3_8056_0
5_CSF11_3_8056_6
5_CSF12_3_8056_12
5_CSF13_1_7090033_0
5_CSF14_1_7090033_16
5_CSF15_1_7090045_0
5_CSF16_1_7090045_20
5_CSF17_1_7090076_0
5_CSF18_1_7090076_13
5_CSF19_2_8015_0
5_CSF2_3_8005_7
5_CSF20_2_8015_5
5_CSF21_2_8015_11
5_CSF22_2_8028_0
5_CSF23_2_8028_7
5_CSF24_2_8028_13
5_CSF3_3_8005_13
5_CSF4_3_8014_0
5_CSF5_3_8014_8
5_CSF6_3_8014_14
5_CSF7_3_8045_0
5_CSF8_3_8045_7
5_CSF9_3_8045_13
6_CSF1_3_8062_0
6_CSF10_1_7515_0
6_CSF11_1_7516_0
6_CSF12_1_7519_0
6_CSF13_1_7522_0
6_CSF14_1_7528_0
6_CSF15_1_7090079_0
6_CSF16_1_7090079_12
6_CSF17_1_7090083_0
6_CSF18_1_7090083_15
6_CSF19_2_8041_0
6_CSF2_3_8062_7
6_CSF20_2_8041_7
6_CSF21_2_8041_13
6_CSF22_2_8081_0
6_CSF23_2_8081_7
6_CSF24_2_8081_13
6_CSF3_3_8062_16
6_CSF4_3_8072_0
6_CSF5_3_8072_7
6_CSF6_3_8072_15
6_CSF7_3_8091_0
6_CSF8_3_8091_7
6_CSF9_3_8091_14
7_CSF1_3_8108_0
7_CSF10_1_7090084_0
7_CSF11_1_7090084_14
7_CSF12_1_7090085_0
7_CSF13_1_7090085_12
7_CSF14_1_7090086_0
7_CSF15_1_7090086_14
7_CSF16_1_7090092_0
7_CSF17_1_7090092_11
7_CSF18_1_7090093_0
7_CSF19_1_7090093_13
7_CSF2_3_8108_6
7_CSF20_2_8128_0
7_CSF21_2_8128_6
7_CSF22_2_8128_13
7_CSF3_3_8108_12
7_CSF4_3_8109_0
7_CSF5_3_8109_6
7_CSF6_3_8109_12
7_CSF7_3_8136_0
7_CSF8_3_8136_7
7_CSF9_3_8136_13

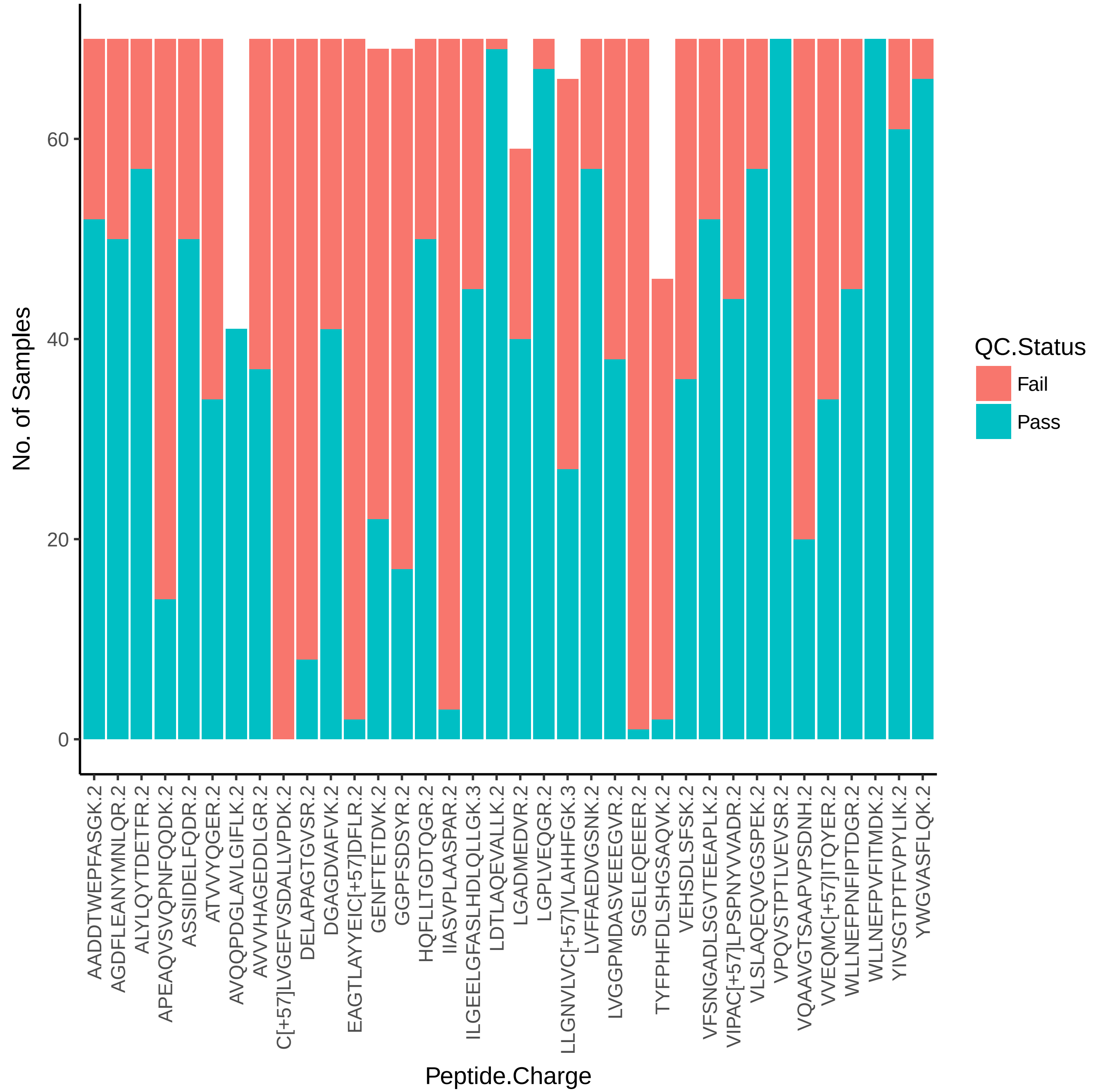
No. of Samples
0
20
40
60
QC.Status
Fail
Pass
Peptide.Charge
AADDTWEPFASGK.2
AGDFLEANYMNLQR.2
ALYLQYTDETFR.2
APEAQVSVQPNFQQDK.2
ASSIIDELFQDR.2
ATVVYQGER.2
AVQQPDGLAVLGIFLK.2
AVVVHAGEDDLGR.2
C[+57]LVGEFVSDALLVPDK.2
DELAPAGTGVSR.2
DGAGDVAFVK.2
EAGTLAYYEIC[+57]DFLR.2
GENFTETDVK.2
GGPFSDSYR.2
HQFLLTGDTQGR.2
IIASVPLAASPAR.2
ILGEELGFASLHDLQLLGK.3
LDTLAQEVALLK.2
LGADMEDVR.2
LGPLVEQGR.2
LLGNVLVC[+57]VLAHHFGK.3
LVFFAEDVGSNK.2
LVGGPMDASVEEEGVR.2
SGELEQEEER.2
TYFPHFDLSHGSAQVK.2
VEHSDLSFSK.2
VFSNGADLSGVTEEAPLK.2
VIPAC[+57]LPSPNYVVADR.2
VLSLAQEQVGGSPEK.2
VPQVSTPTLVEVSR.2
VQAAVGTSAAPVPSDNH.2
VVEQMC[+57]ITQYER.2
WLLNEFPNFIPTDGR.2
WLLNEFPVFITMDK.2
YIVSGTPTFVPYLIK.2
YWGVASFLQK.2

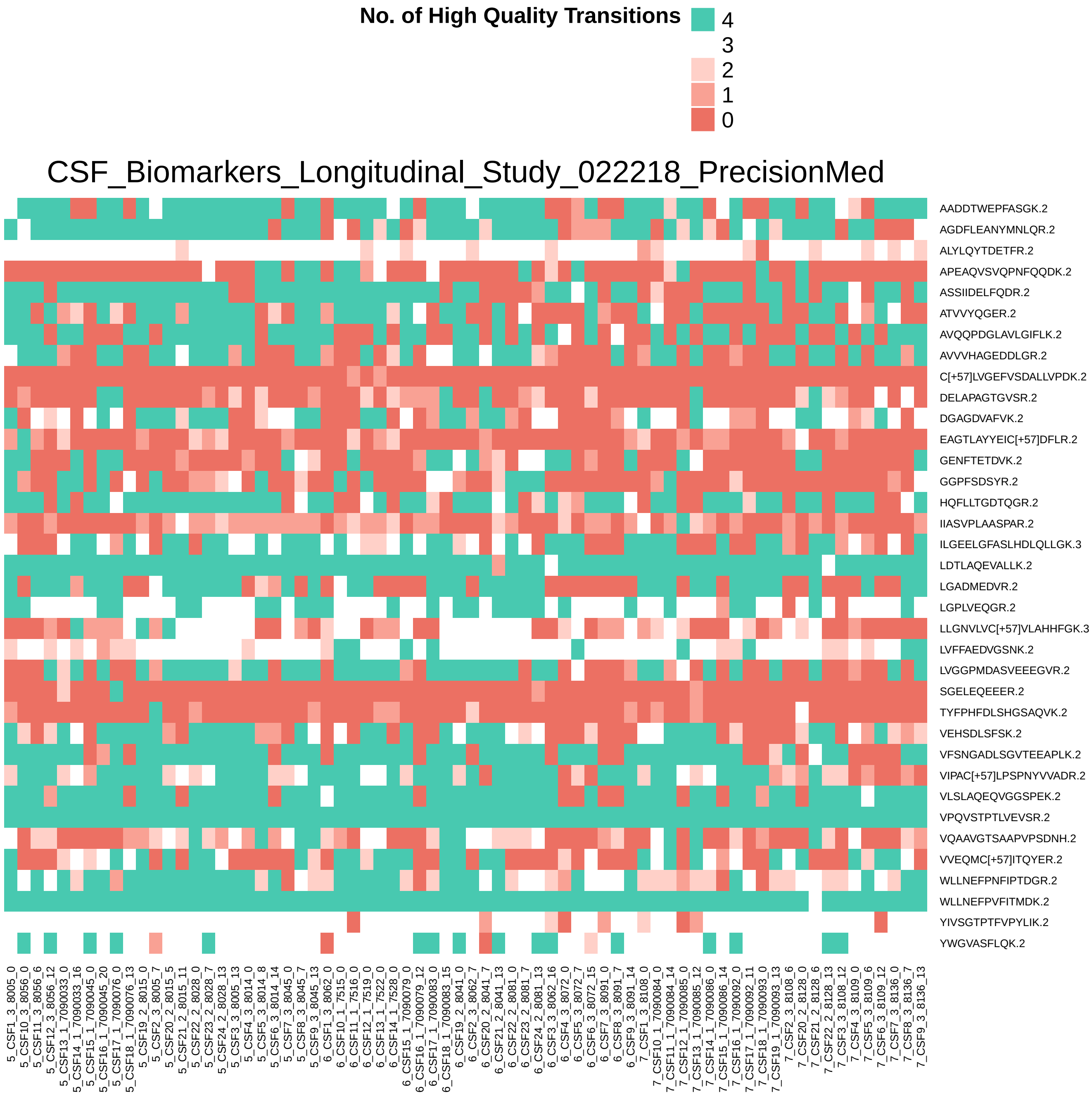
No. of High Quality Transitions
4
3
2
1
0
CSF_Biomarkers_Longitudinal_Study_022218_PrecisionMed
AADDTWEPFASGK.2
AGDFLEANYMNLQR.2
ALYLQYTDETFR.2
APEAQVSVQPNFQQDK.2
ASSIIDELFQDR.2
ATVVYQGER.2
AVQQPDGLAVLGIFLK.2
AVVVHAGEDDLGR.2
C[+57]LVGEFVSDALLVPDK.2
DELAPAGTGVSR.2
DGAGDVAFVK.2
EAGTLAYYEIC[+57]DFLR.2
GENFTETDVK.2
GGPFSDSYR.2
HQFLLTGDTQGR.2
IIASVPLAASPAR.2
ILGEELGFASLHDLQLLGK.3
LDTLAQEVALLK.2
LGADMEDVR.2
LGPLVEQGR.2
LLGNVLVC[+57]VLAHHFGK.3
LVFFAEDVGSNK.2
LVGGPMDASVEEEGVR.2
SGELEQEEER.2
TYFPHFDLSHGSAQVK.2
VEHSDLSFSK.2
VFSNGADLSGVTEEAPLK.2
VIPAC[+57]LPSPNYVVADR.2
VLSLAQEQVGGSPEK.2
VPQVSTPTLVEVSR.2
VQAAVGTSAAPVPSDNH.2
VVEQMC[+57]ITQYER.2
WLLNEFPNFIPTDGR.2
WLLNEFPVFITMDK.2
YIVSGTPTFVPYLIK.2
YWGVASFLQK.2
5_CSF1_3_8005_0
5_CSF10_3_8056_0
5_CSF11_3_8056_6
5_CSF12_3_8056_12
5_CSF13_1_7090033_0
5_CSF14_1_7090033_16
5_CSF15_1_7090045_0
5_CSF16_1_7090045_20
5_CSF17_1_7090076_0
5_CSF18_1_7090076_13
5_CSF19_2_8015_0
5_CSF2_3_8005_7
5_CSF20_2_8015_5
5_CSF21_2_8015_11
5_CSF22_2_8028_0
5_CSF23_2_8028_7
5_CSF24_2_8028_13
5_CSF3_3_8005_13
5_CSF4_3_8014_0
5_CSF5_3_8014_8
5_CSF6_3_8014_14
5_CSF7_3_8045_0
5_CSF8_3_8045_7
5_CSF9_3_8045_13
6_CSF1_3_8062_0
6_CSF10_1_7515_0
6_CSF11_1_7516_0
6_CSF12_1_7519_0
6_CSF13_1_7522_0
6_CSF14_1_7528_0
6_CSF15_1_7090079_0
6_CSF16_1_7090079_12
6_CSF17_1_7090083_0
6_CSF18_1_7090083_15
6_CSF19_2_8041_0
6_CSF2_3_8062_7
6_CSF20_2_8041_7
6_CSF21_2_8041_13
6_CSF22_2_8081_0
6_CSF23_2_8081_7
6_CSF24_2_8081_13
6_CSF3_3_8062_16
6_CSF4_3_8072_0
6_CSF5_3_8072_7
6_CSF6_3_8072_15
6_CSF7_3_8091_0
6_CSF8_3_8091_7
6_CSF9_3_8091_14
7_CSF1_3_8108_0
7_CSF10_1_7090084_0
7_CSF11_1_7090084_14
7_CSF12_1_7090085_0
7_CSF13_1_7090085_12
7_CSF14_1_7090086_0
7_CSF15_1_7090086_14
7_CSF16_1_7090092_0
7_CSF17_1_7090092_11
7_CSF18_1_7090093_0
7_CSF19_1_7090093_13
7_CSF2_3_8108_6
7_CSF20_2_8128_0
7_CSF21_2_8128_6
7_CSF22_2_8128_13
7_CSF3_3_8108_12
7_CSF4_3_8109_0
7_CSF5_3_8109_6
7_CSF6_3_8109_12
7_CSF7_3_8136_0
7_CSF8_3_8136_7
7_CSF9_3_8136_13

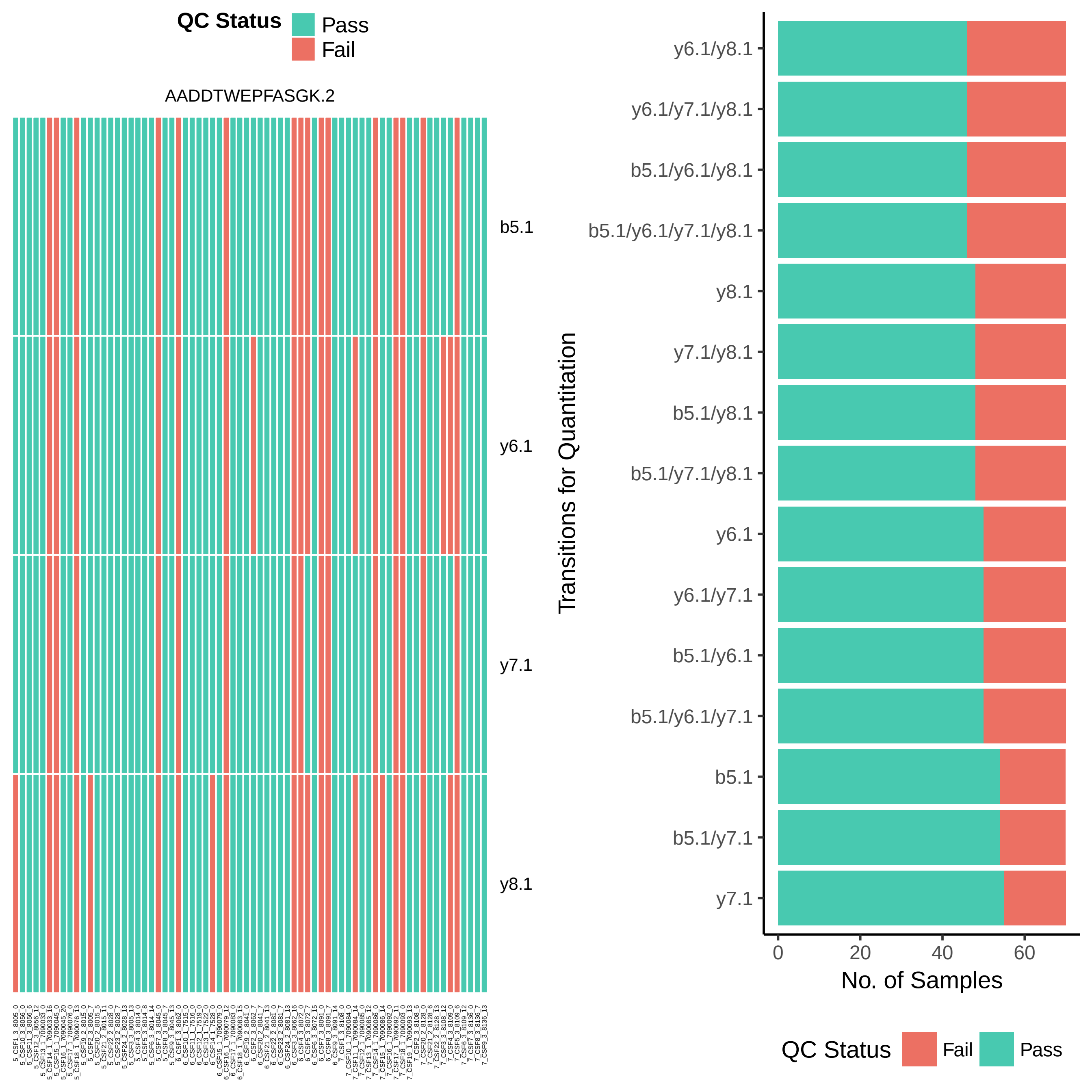
QC Status
Pass
Fail
AADDTWEPFASGK.2
b5.1
y6.1
y7.1
y8.1
y6.1/y8.1
y6.1/y7.1/y8.1
b5.1/y6.1/y8.1
b5.1/y6.1/y7.1/y8.1
y8.1
y7.1/y8.1
b5.1/y8.1
b5.1/y7.1/y8.1
y6.1
y6.1/y7.1
b5.1/y6.1
b5.1/y6.1/y7.1
b5.1
b5.1/y7.1
y7.1
Transitions for Quantitation
0
20
40
60
No. of Samples
QC Status
Fail
Pass

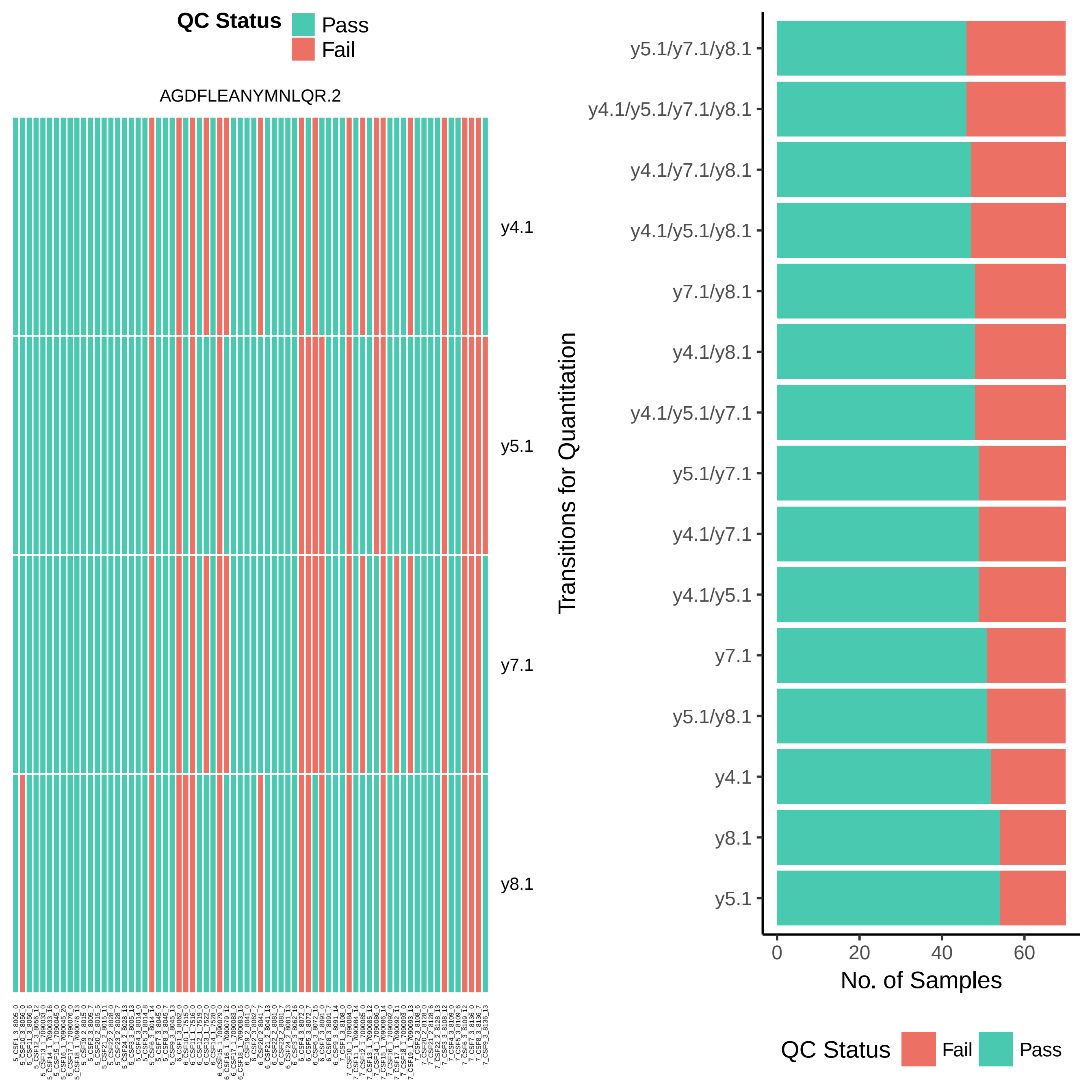
QC Status
Pass
Fail
AGDFLEANYMNLQR.2
y4.1
y5.1
y7.1
y8.1
Transitions for Quantitation
y5.1/y7.1/y8.1
y4.1/y5.1/y7.1/y8.1
y4.1/y7.1/y8.1
y4.1/y5.1/y8.1
y7.1/y8.1
y4.1/y8.1
y4.1/y5.1/y7.1
y5.1/y7.1
y4.1/y7.1
y4.1/y5.1
y7.1
y5.1/y8.1
y4.1
y8.1
y5.1
0
20
40
60
No. of Samples
QC Status
Fail
Pass

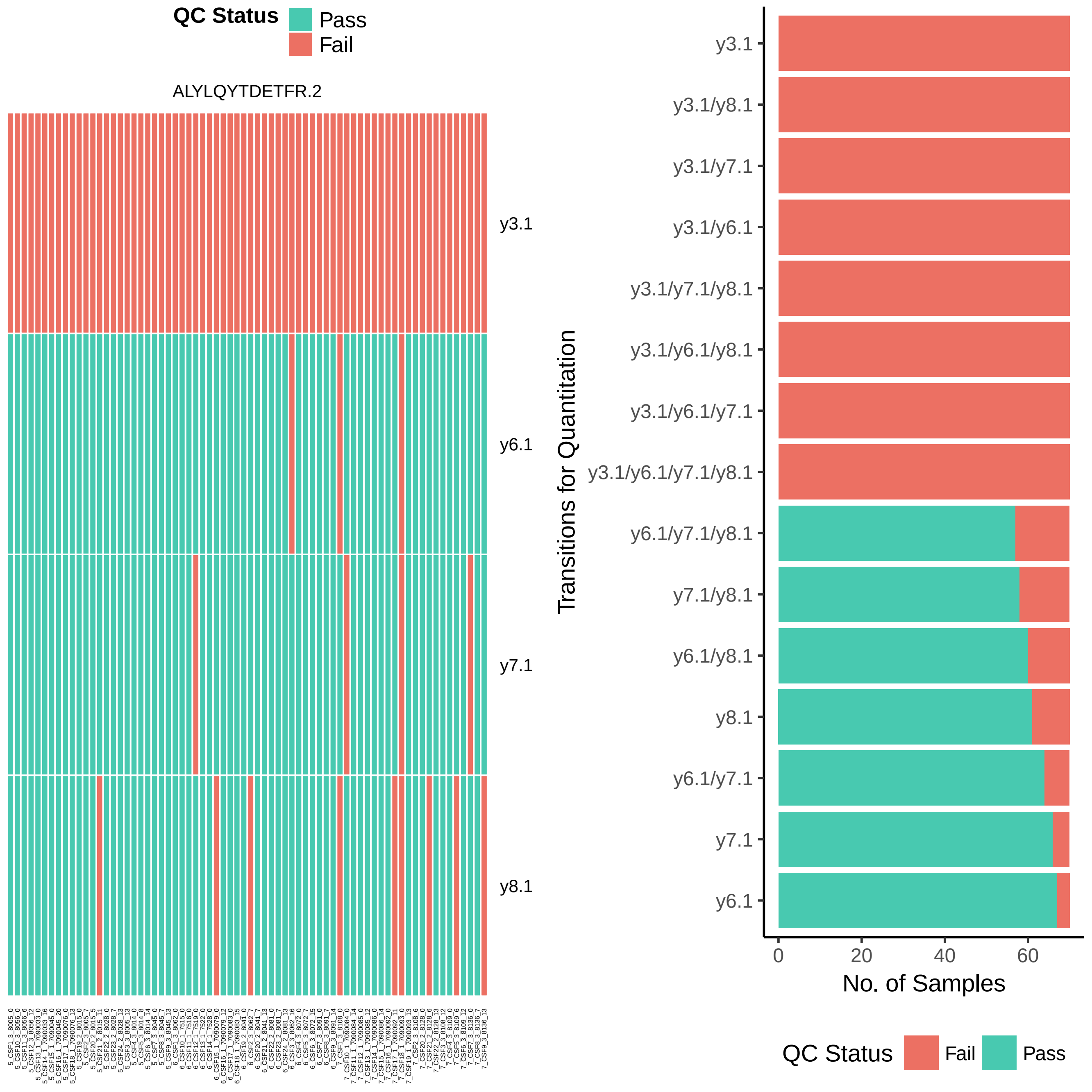
QC Status
Pass
Fail
ALYLQYTDETFR.2
y3.1
y6.1
y7.1
y8.1
Transitions for Quantitation
y3.1
y3.1/y8.1
y3.1/y7.1
y3.1/y6.1
y3.1/y7.1/y8.1
y3.1/y6.1/y8.1
y3.1/y6.1/y7.1
y3.1/y6.1/y7.1/y8.1
y6.1/y7.1/y8.1
y7.1/y8.1
y6.1/y8.1
y8.1
y6.1/y7.1
y7.1
y6.1
0
20
40
60
No. of Samples
QC Status
Fail
Pass

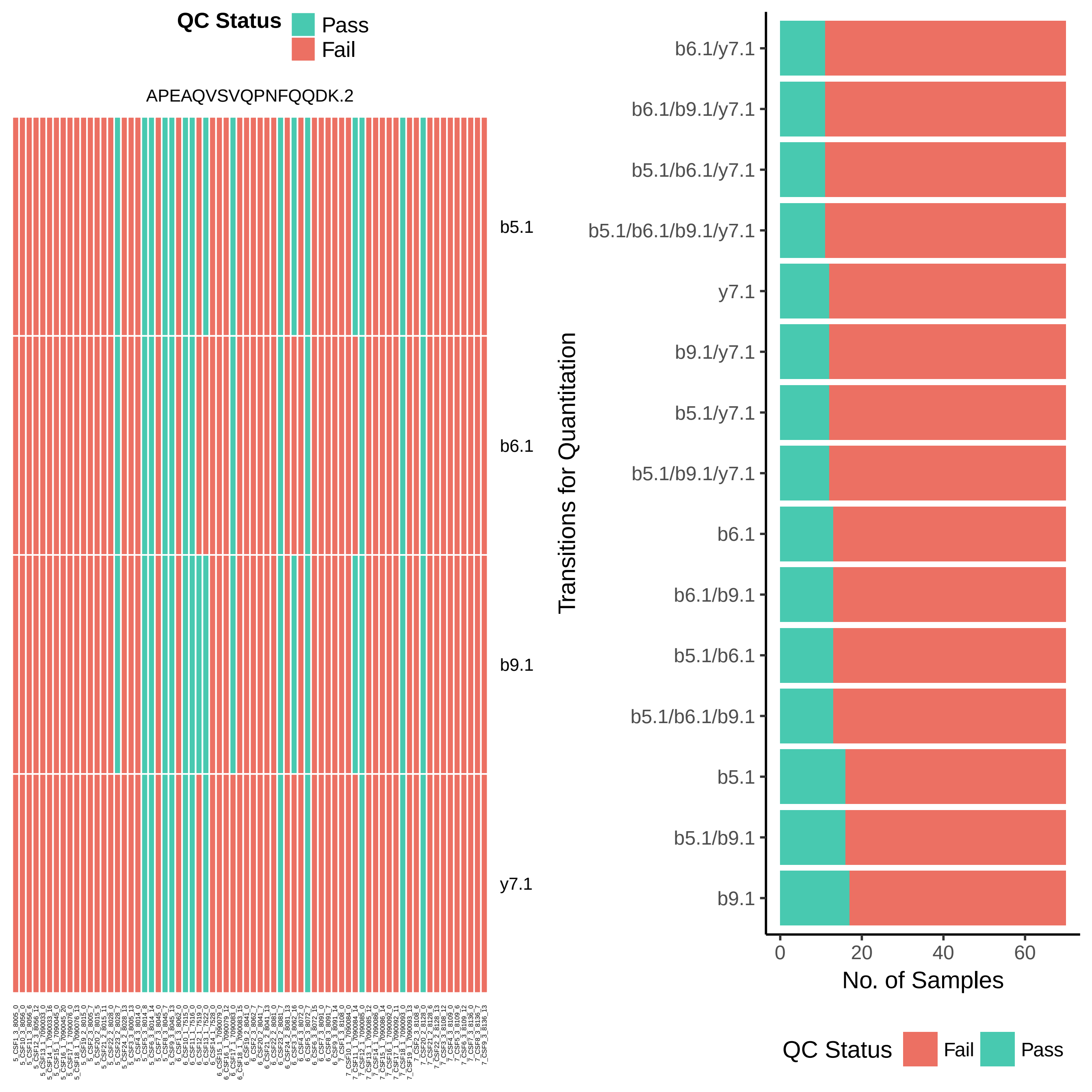
QC Status
Pass
Fail
APEAQVSVQPNFQQDK.2
b5.1
b6.1
b9.1
y7.1
b6.1/y7.1
b6.1/b9.1/y7.1
b5.1/b6.1/y7.1
b5.1/b6.1/b9.1/y7.1
y7.1
b9.1/y7.1
b5.1/y7.1
b5.1/b9.1/y7.1
b6.1
b6.1/b9.1
b5.1/b6.1
b5.1/b6.1/b9.1
b5.1
b5.1/b9.1
b9.1
Transitions for Quantitation
0
20
40
60
No. of Samples
QC Status
Fail
Pass

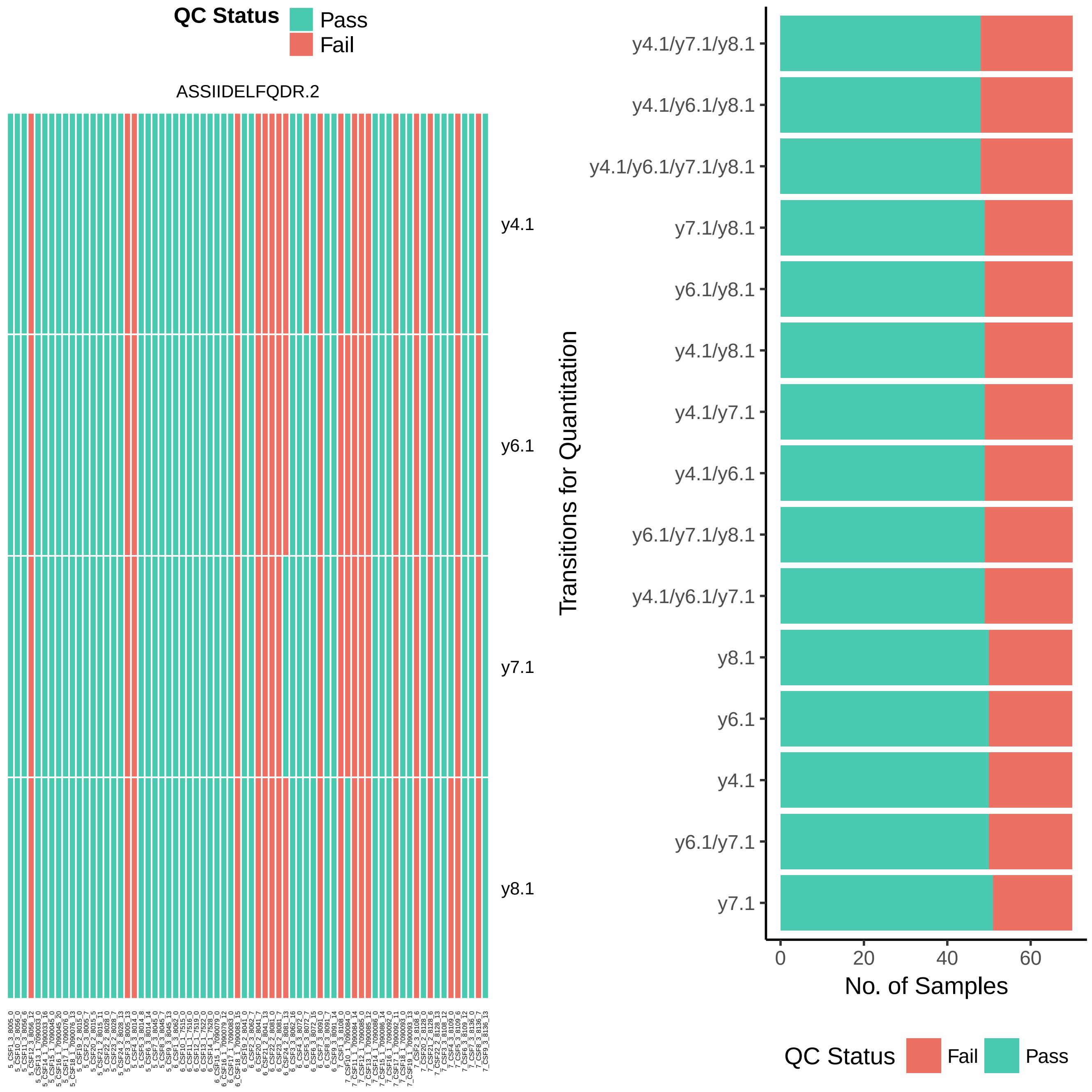

QC Status
Pass
Fail
ASSIIDELFQDR.2
y4.1
y6.1
y7.1
y8.1
Transitions for Quantitation
y4.1/y7.1/y8.1
y4.1/y6.1/y8.1
y4.1/y6.1/y7.1/y8.1
y7.1/y8.1
y6.1/y8.1
y4.1/y8.1
y4.1/y7.1
y4.1/y6.1
y6.1/y7.1/y8.1
y4.1/y6.1/y7.1
y8.1
y6.1
y4.1
y6.1/y7.1
y7.1
0
20
40
60
No. of Samples
QC Status
Fail
Pass

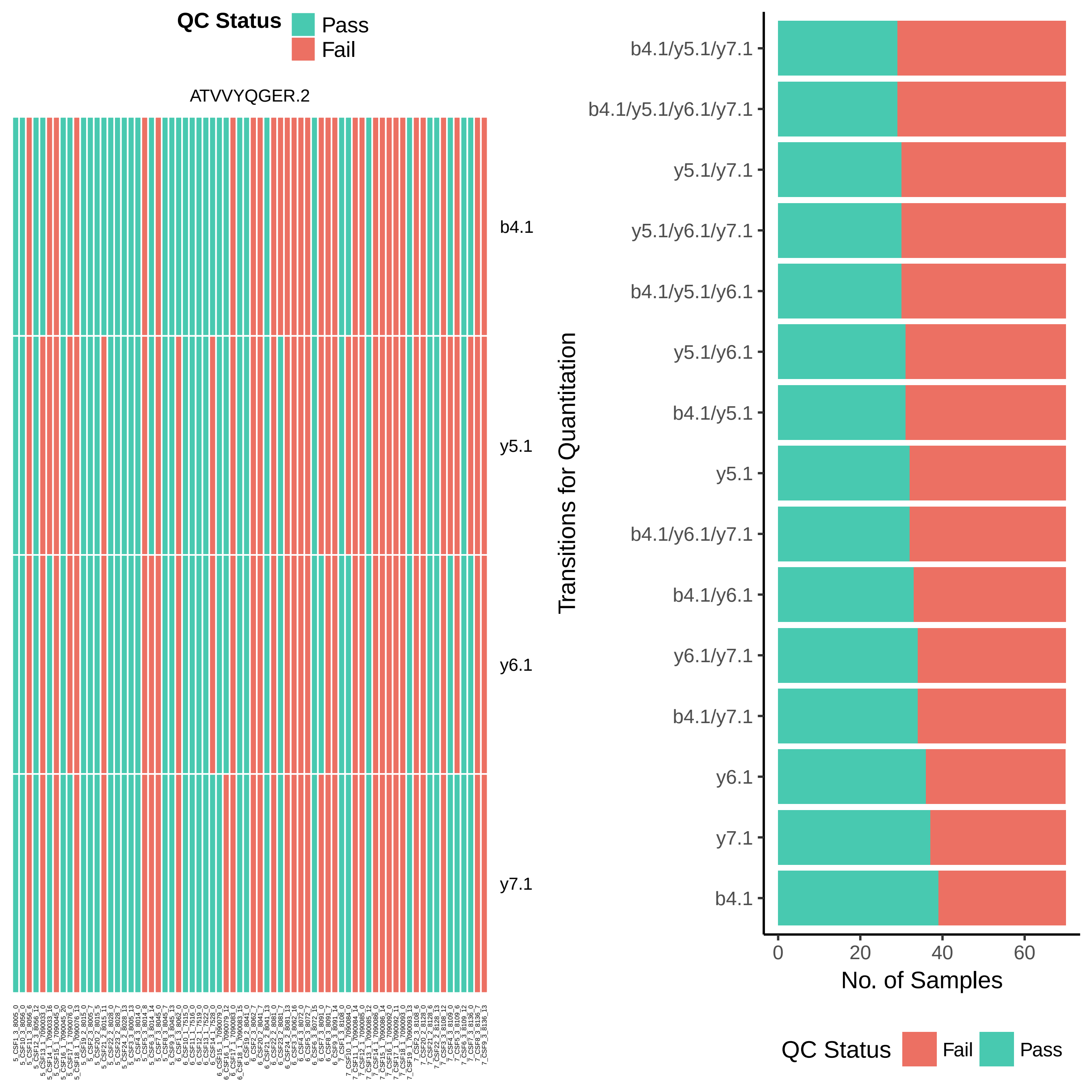
QC Status
Pass
Fail
ATVVYQGER.2
b4.1
y5.1
y6.1
y7.1
Transitions for Quantitation
b4.1/y5.1/y7.1
b4.1/y5.1/y6.1/y7.1
y5.1/y7.1
y5.1/y6.1/y7.1
b4.1/y5.1/y6.1
y5.1/y6.1
b4.1/y5.1
y5.1
b4.1/y6.1/y7.1
b4.1/y6.1
y6.1/y7.1
b4.1/y7.1
y6.1
y7.1
b4.1
0
20
40
60
No. of Samples
QC Status
Fail
Pass

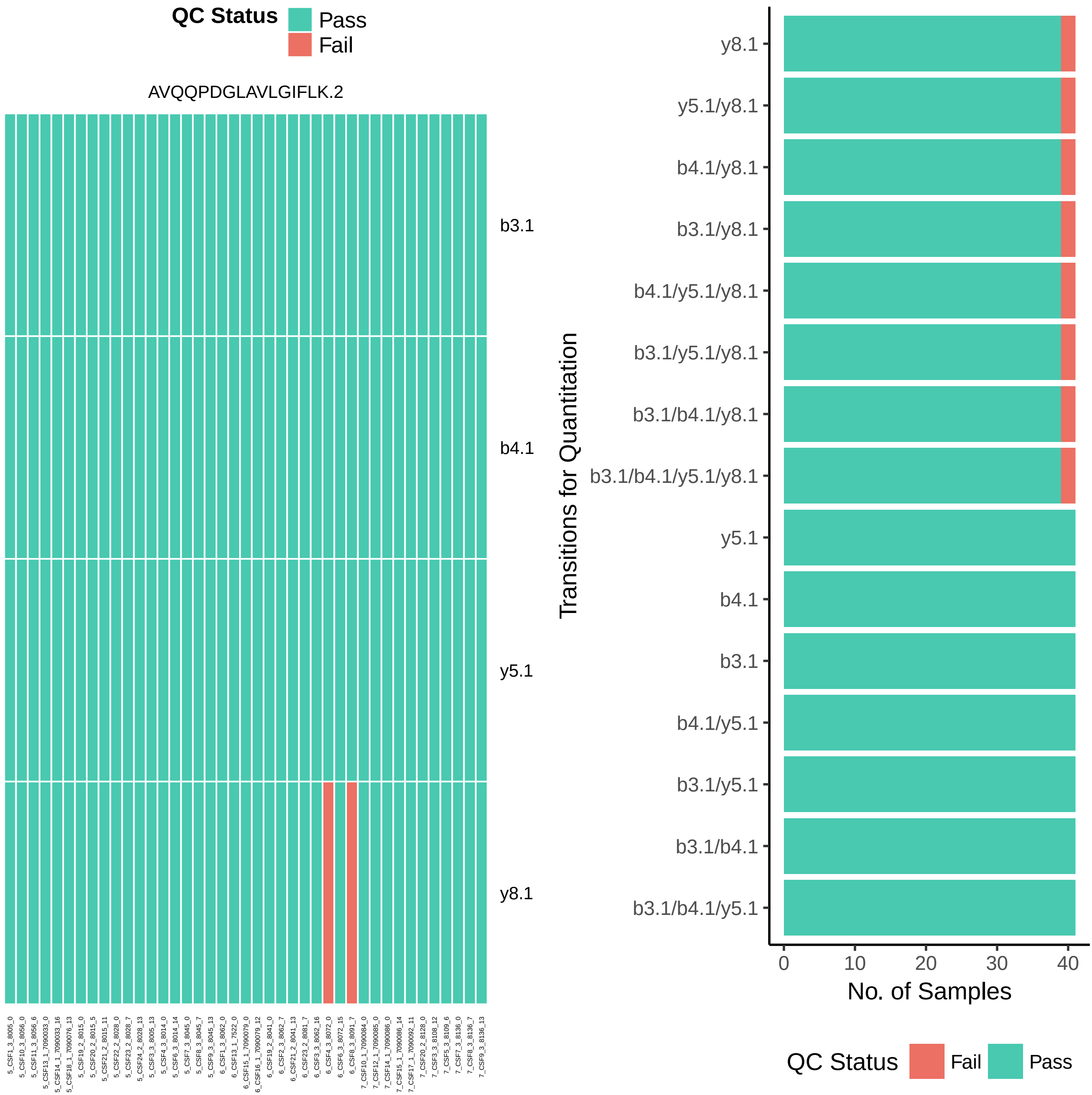

QC Status
Pass
Fail
AVQQPDGLAVLGIFLK.2
b3.1
b4.1
y5.1
y8.1
5_CSF1_3_8005_0
5_CSF10_3_8056_0
5_CSF11_3_8056_6
5_CSF13_1_7090033_0
5_CSF14_1_7090033_16
5_CSF18_1_7090076_13
5_CSF19_2_8015_0
5_CSF20_2_8015_5
5_CSF21_2_8015_11
5_CSF22_2_8028_0
5_CSF23_2_8028_7
5_CSF24_2_8028_13
5_CSF3_3_8005_13
5_CSF4_3_8014_0
5_CSF6_3_8014_14
5_CSF7_3_8045_0
5_CSF8_3_8045_7
5_CSF9_3_8045_13
6_CSF1_3_8062_0
6_CSF13_1_7522_0
6_CSF15_1_7090079_0
6_CSF16_1_7090079_12
6_CSF19_2_8041_0
6_CSF2_3_8062_7
6_CSF21_2_8041_13
6_CSF23_2_8081_7
6_CSF3_3_8062_16
6_CSF4_3_8072_0
6_CSF6_3_8072_15
6_CSF8_3_8091_7
7_CSF10_1_7090084_0
7_CSF12_1_7090085_0
7_CSF14_1_7090086_0
7_CSF15_1_7090086_14
7_CSF17_1_7090092_11
7_CSF20_2_8128_0
7_CSF3_3_8108_12
7_CSF5_3_8109_6
7_CSF7_3_8136_0
7_CSF8_3_8136_7
7_CSF9_3_8136_13
Transitions for Quantitation
y8.1
y5.1/y8.1
b4.1/y8.1
b3.1/y8.1
b4.1/y5.1/y8.1
b3.1/y5.1/y8.1
b3.1/b4.1/y8.1
b3.1/b4.1/y5.1/y8.1
y5.1
b4.1
b3.1
b4.1/y5.1
b3.1/y5.1
b3.1/b4.1
b3.1/b4.1/y5.1
0
10
20
30
40
No. of Samples
QC Status
Fail
Pass

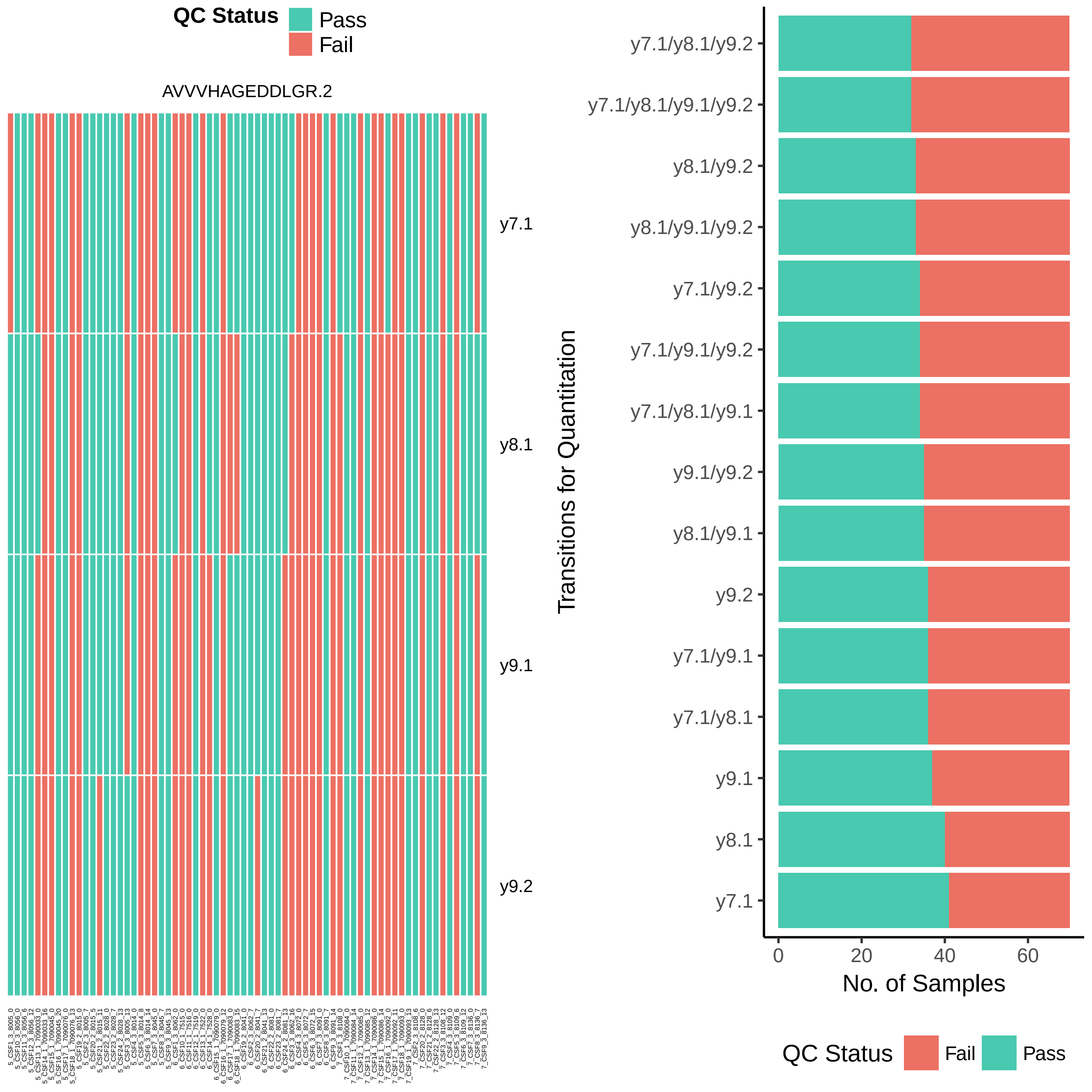

QC Status
Pass
Fail
AVVVHAGEDDLGR.2
y7.1
y8.1
y9.1
y9.2
Transitions for Quantitation
y7.1/y8.1/y9.2
y7.1/y8.1/y9.1/y9.2
y8.1/y9.2
y8.1/y9.1/y9.2
y7.1/y9.2
y7.1/y9.1/y9.2
y7.1/y8.1/y9.1
y9.1/y9.2
y8.1/y9.1
y9.2
y7.1/y9.1
y7.1/y8.1
y9.1
y8.1
y7.1
0
20
40
60
No. of Samples
QC Status
Fail
Pass

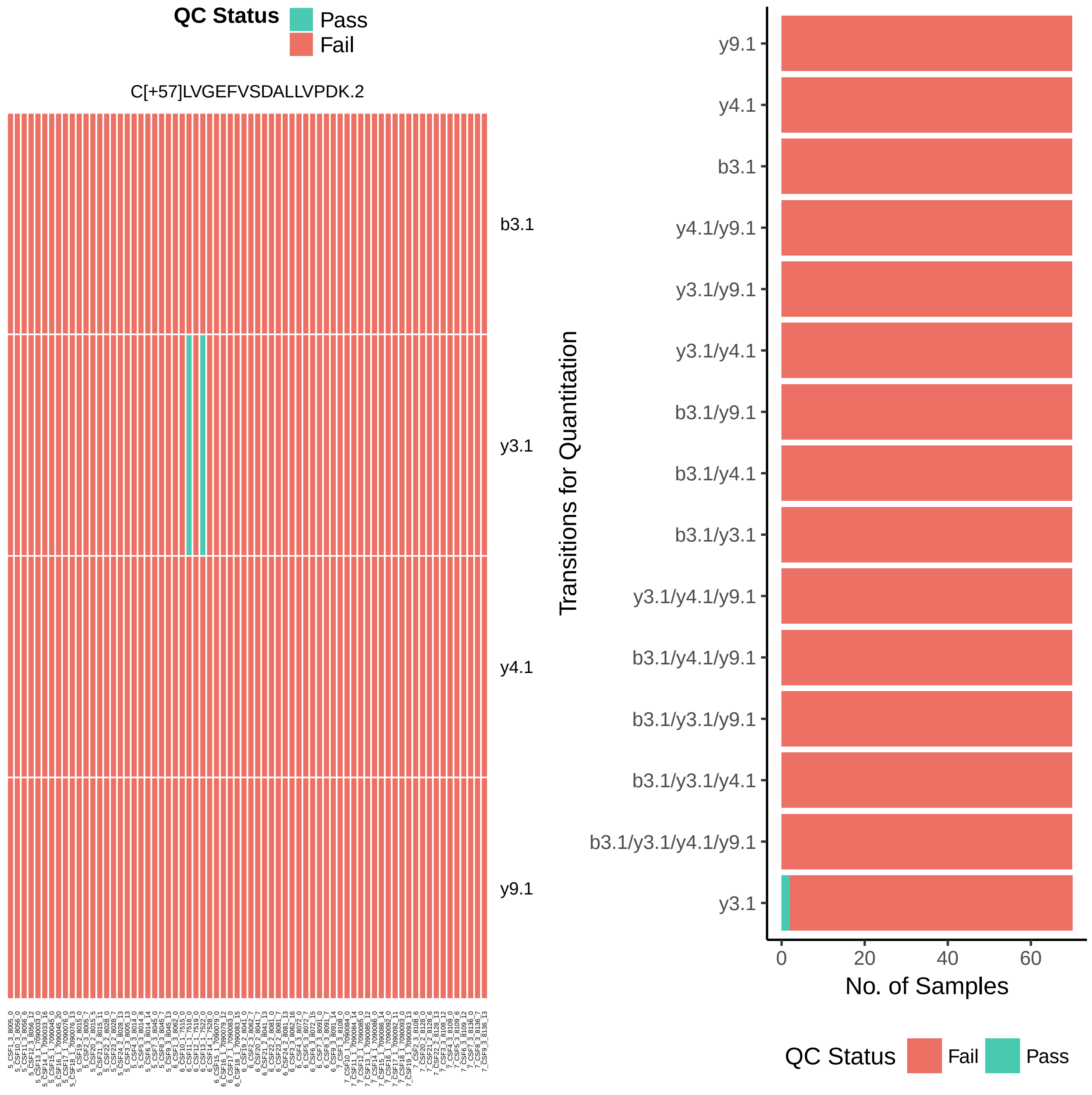
QC Status
Pass
Fail
C[+57]LVGEFVSDALLVPDK.2
b3.1
y3.1
y4.1
y9.1
Transitions for Quantitation
y9.1
y4.1
b3.1
y4.1/y9.1
y3.1/y9.1
y3.1/y4.1
b3.1/y9.1
b3.1/y4.1
b3.1/y3.1
y3.1/y4.1/y9.1
b3.1/y4.1/y9.1
b3.1/y3.1/y9.1
b3.1/y3.1/y4.1
b3.1/y3.1/y4.1/y9.1
y3.1
0
20
40
60
No. of Samples
QC Status
Fail
Pass

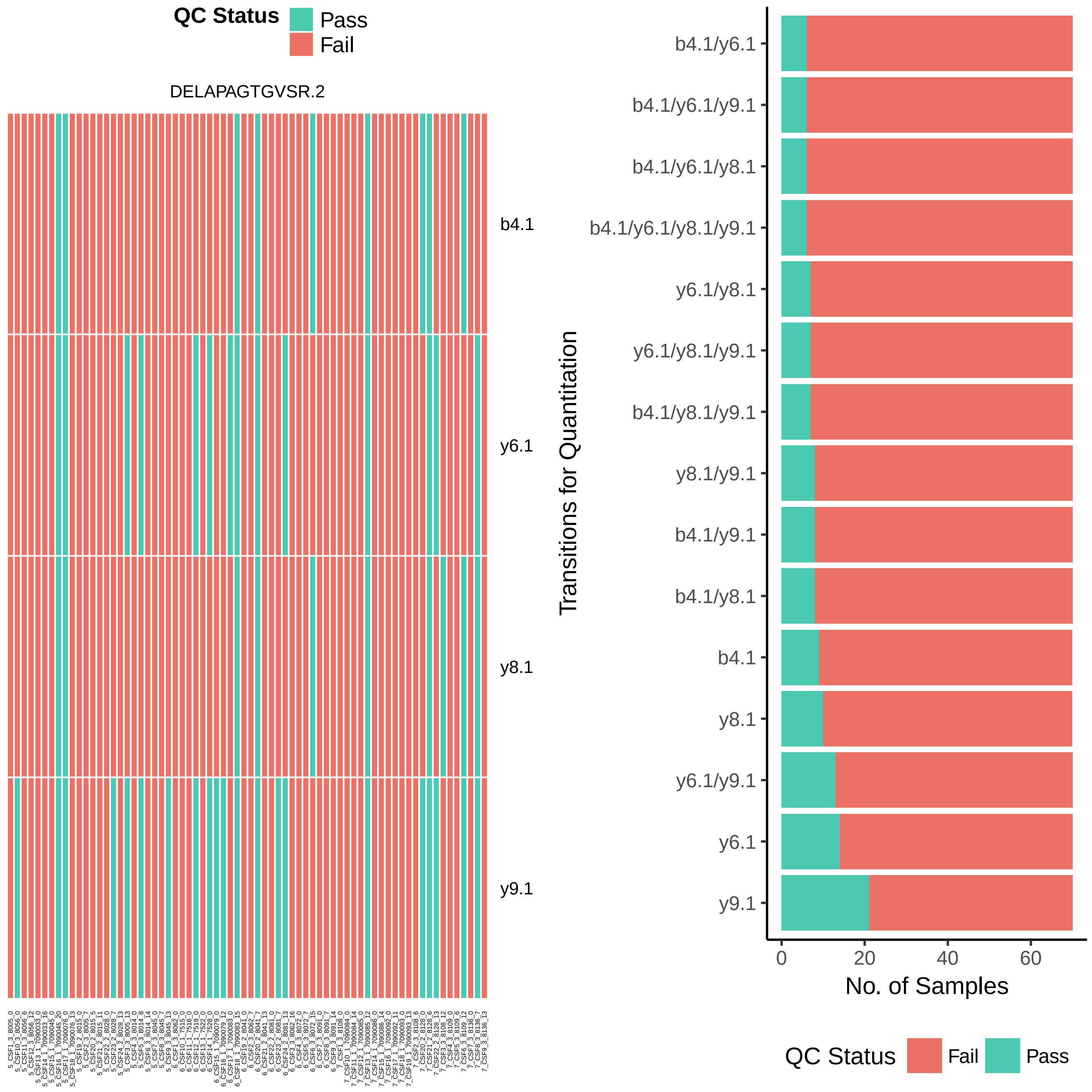
QC Status
Pass
Fail
DELAPAGTGVSR.2
b4.1
y6.1
y8.1
y9.1
b4.1/y6.1
b4.1/y6.1/y9.1
b4.1/y6.1/y8.1
b4.1/y6.1/y8.1/y9.1
y6.1/y8.1
y6.1/y8.1/y9.1
b4.1/y8.1/y9.1
y8.1/y9.1
b4.1/y9.1
b4.1/y8.1
b4.1
y8.1
y6.1/y9.1
y6.1
y9.1
Transitions for Quantitation
0
20
40
60
No. of Samples
QC Status
Fail
Pass

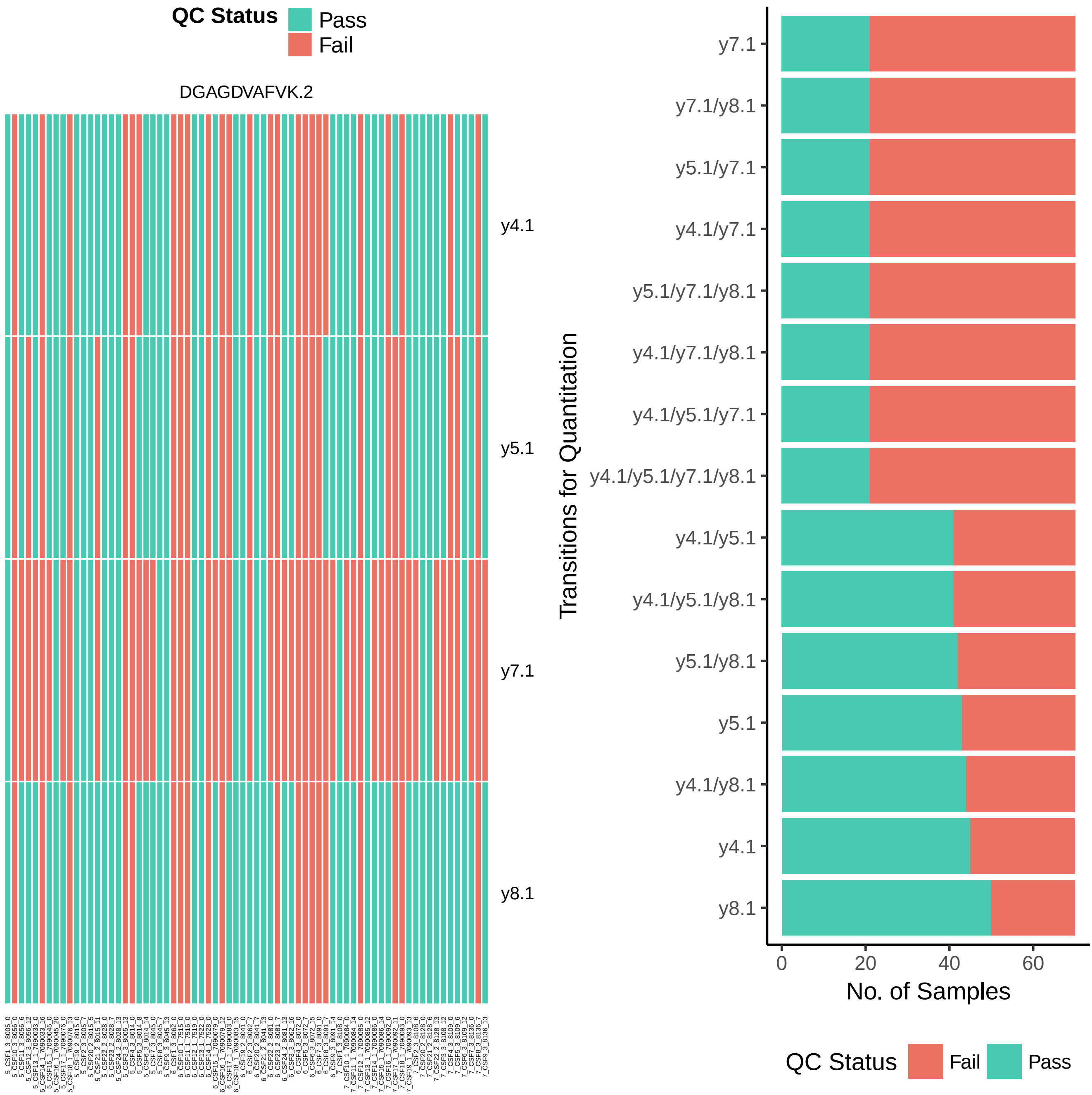
QC Status
Pass
Fail
DGAGDVAFVK.2
y4.1
y5.1
y7.1
y8.1
Transitions for Quantitation
y7.1
y7.1/y8.1
y5.1/y7.1
y4.1/y7.1
y5.1/y7.1/y8.1
y4.1/y7.1/y8.1
y4.1/y5.1/y7.1
y4.1/y5.1/y7.1/y8.1
y4.1/y5.1
y4.1/y5.1/y8.1
y5.1/y8.1
y5.1
y4.1/y8.1
y4.1
y8.1
0
20
40
60
No. of Samples
QC Status
Fail
Pass

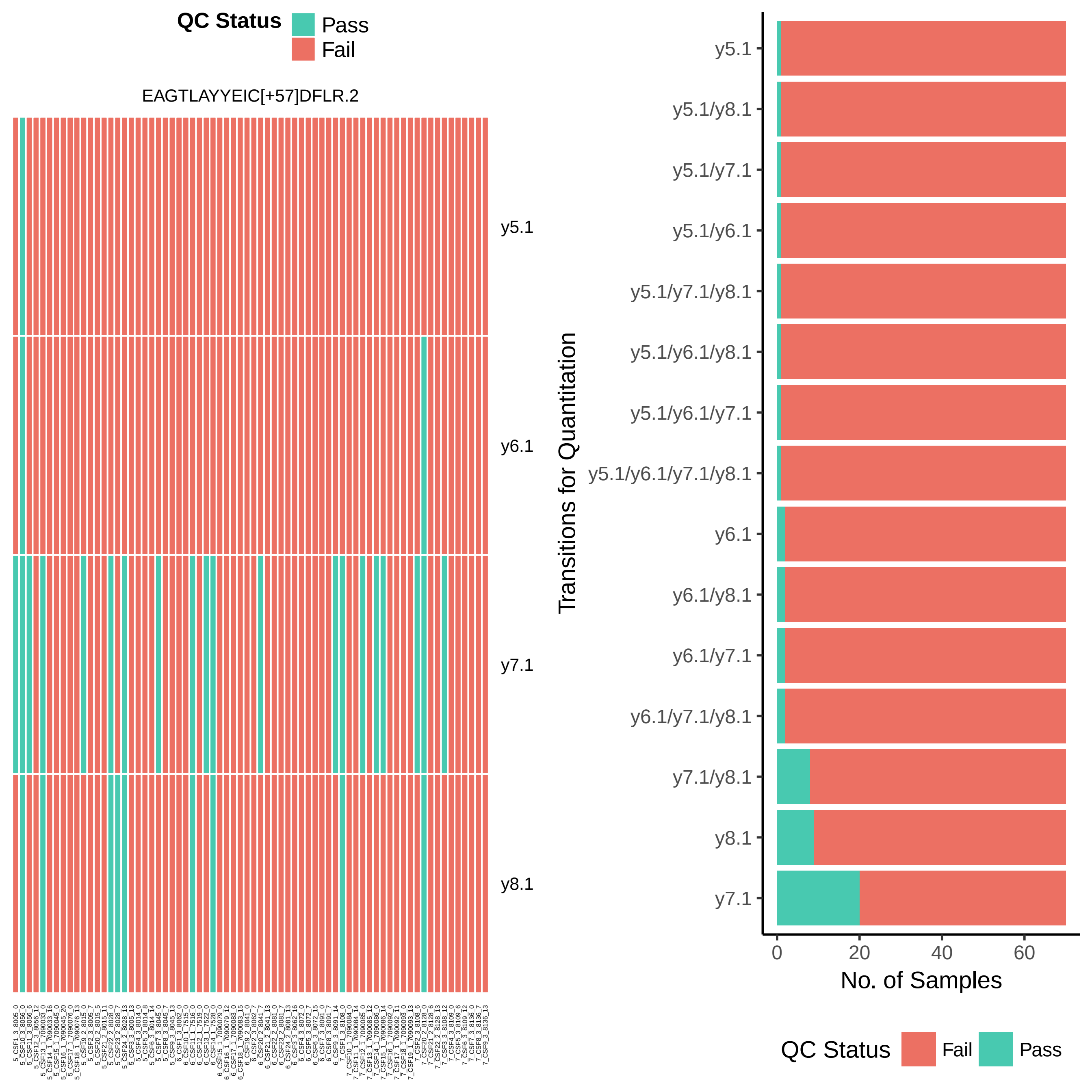
QC Status
Pass
Fail
EAGTLAYYEIC[+57]DFLR.2
y5.1
y6.1
y7.1
y8.1
Transitions for Quantitation
y5.1
y5.1/y8.1
y5.1/y7.1
y5.1/y6.1
y5.1/y7.1/y8.1
y5.1/y6.1/y8.1
y5.1/y6.1/y7.1
y5.1/y6.1/y7.1/y8.1
y6.1
y6.1/y8.1
y6.1/y7.1
y6.1/y7.1/y8.1
y7.1/y8.1
y8.1
y7.1
0
20
40
60
No. of Samples
QC Status
Fail
Pass

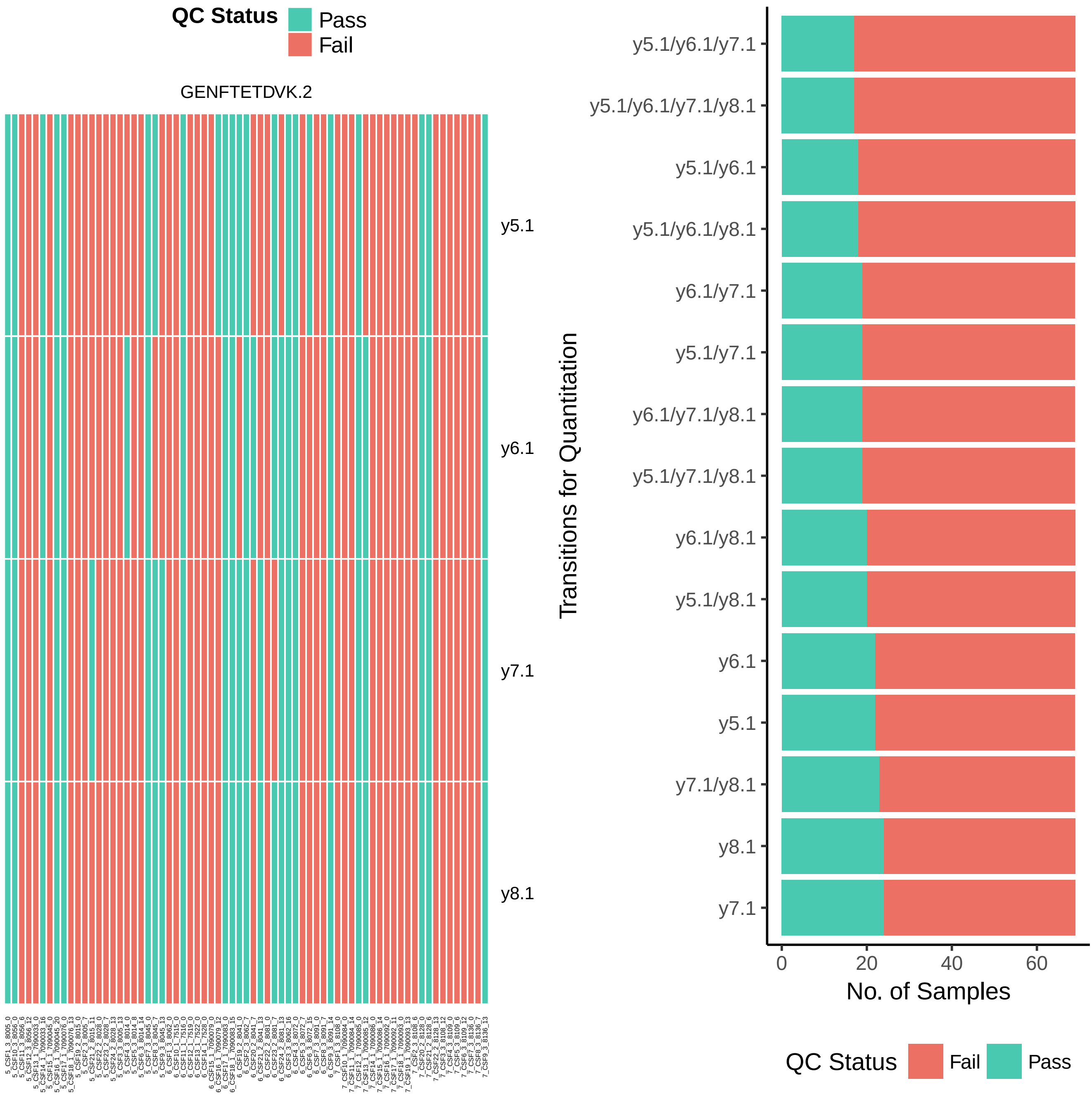
QC Status
Pass
Fail
GENFTETDVK.2
y5.1
y6.1
y7.1
y8.1
Transitions for Quantitation
y5.1/y6.1/y7.1
y5.1/y6.1/y7.1/y8.1
y5.1/y6.1
y5.1/y6.1/y8.1
y6.1/y7.1
y5.1/y7.1
y6.1/y7.1/y8.1
y5.1/y7.1/y8.1
y6.1/y8.1
y5.1/y8.1
y6.1
y5.1
y7.1/y8.1
y8.1
y7.1
0
20
40
60
No. of Samples
QC Status
Fail
Pass

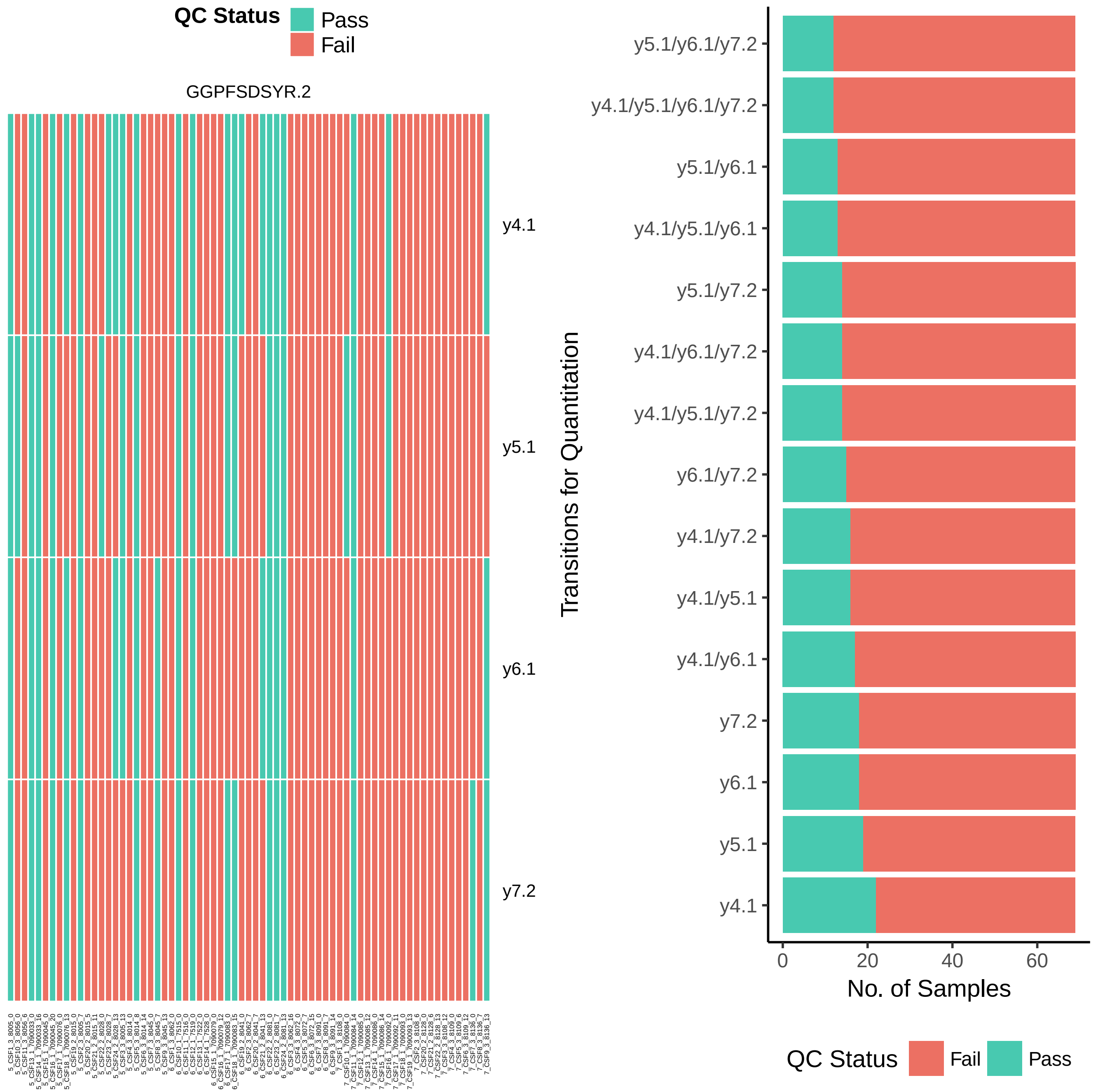
QC Status
Pass
Fail
GGPFSDSYR.2
y4.1
y5.1
y6.1
y7.2
y5.1/y6.1/y7.2
y4.1/y5.1/y6.1/y7.2
y5.1/y6.1
y4.1/y5.1/y6.1
y5.1/y7.2
y4.1/y6.1/y7.2
y4.1/y5.1/y7.2
y6.1/y7.2
y4.1/y7.2
y4.1/y5.1
y4.1/y6.1
y7.2
y6.1
y5.1
y4.1
Transitions for Quantitation
0
20
40
60
No. of Samples
QC Status
Fail
Pass

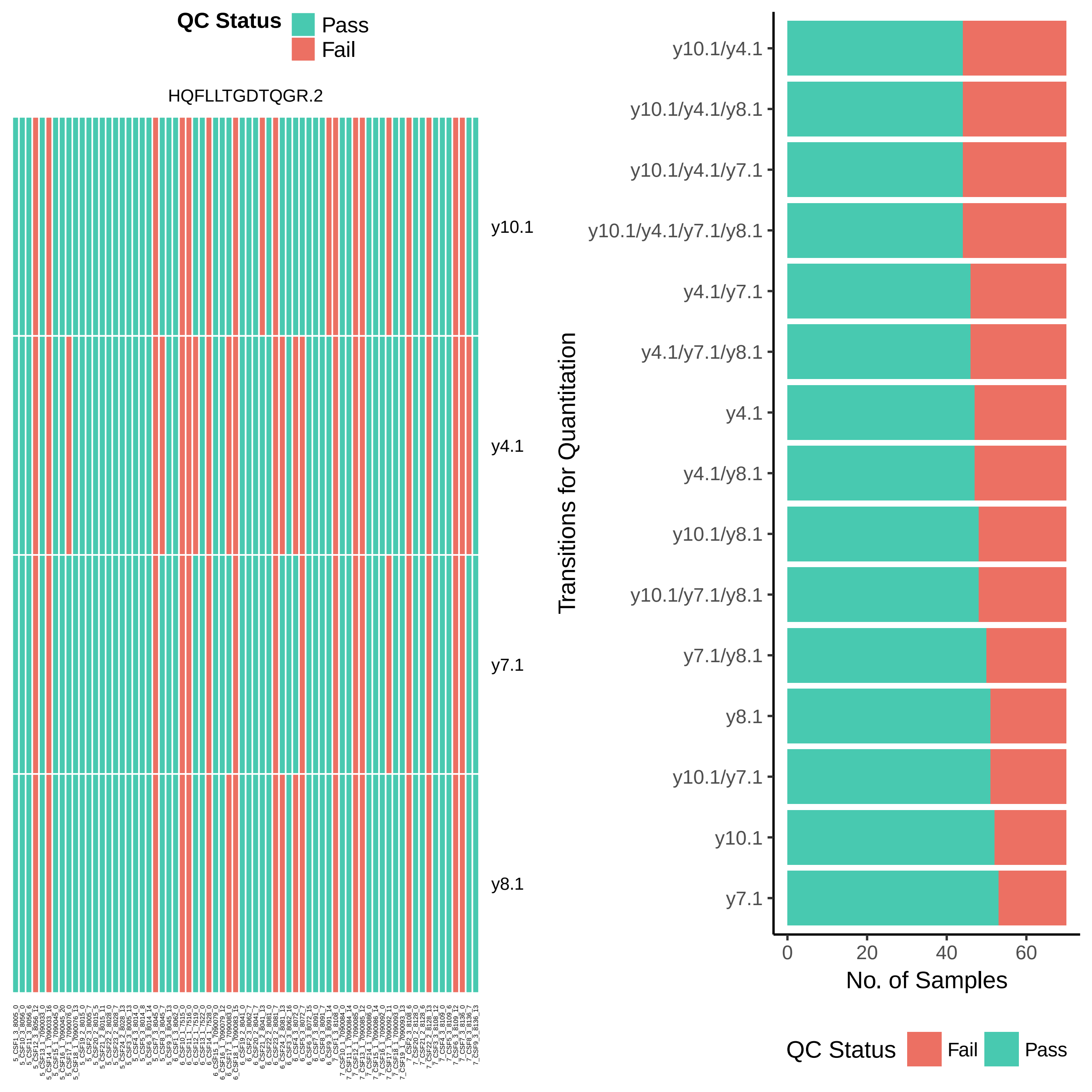

QC Status
Pass
Fail
HQFLLTGDTQGR.2
y10.1
y4.1
y7.1
y8.1
Transitions for Quantitation
y10.1/y4.1
y10.1/y4.1/y8.1
y10.1/y4.1/y7.1
y10.1/y4.1/y7.1/y8.1
y4.1/y7.1
y4.1/y7.1/y8.1
y4.1
y4.1/y8.1
y10.1/y8.1
y10.1/y7.1/y8.1
y7.1/y8.1
y8.1
y10.1/y7.1
y10.1
y7.1
0
20
40
60
No. of Samples
QC Status
Fail
Pass

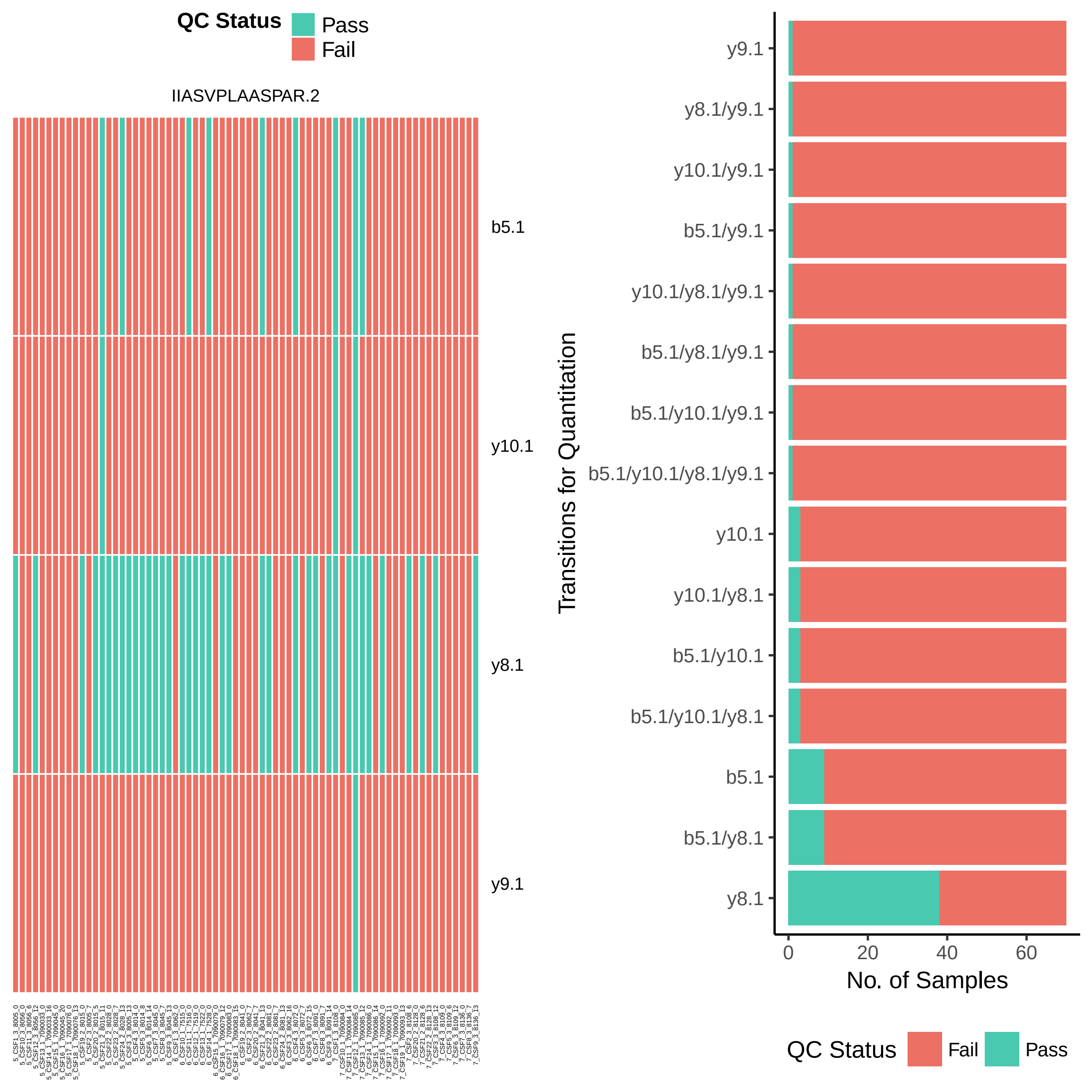
QC Status
Pass
Fail
IIASVPLAASPAR.2
b5.1
y10.1
y8.1
y9.1
Transitions for Quantitation
y9.1
y8.1/y9.1
y10.1/y9.1
b5.1/y9.1
y10.1/y8.1/y9.1
b5.1/y8.1/y9.1
b5.1/y10.1/y9.1
b5.1/y10.1/y8.1/y9.1
y10.1
y10.1/y8.1
b5.1/y10.1
b5.1/y10.1/y8.1
b5.1
b5.1/y8.1
y8.1
0
20
40
60
No. of Samples
QC Status
Fail
Pass

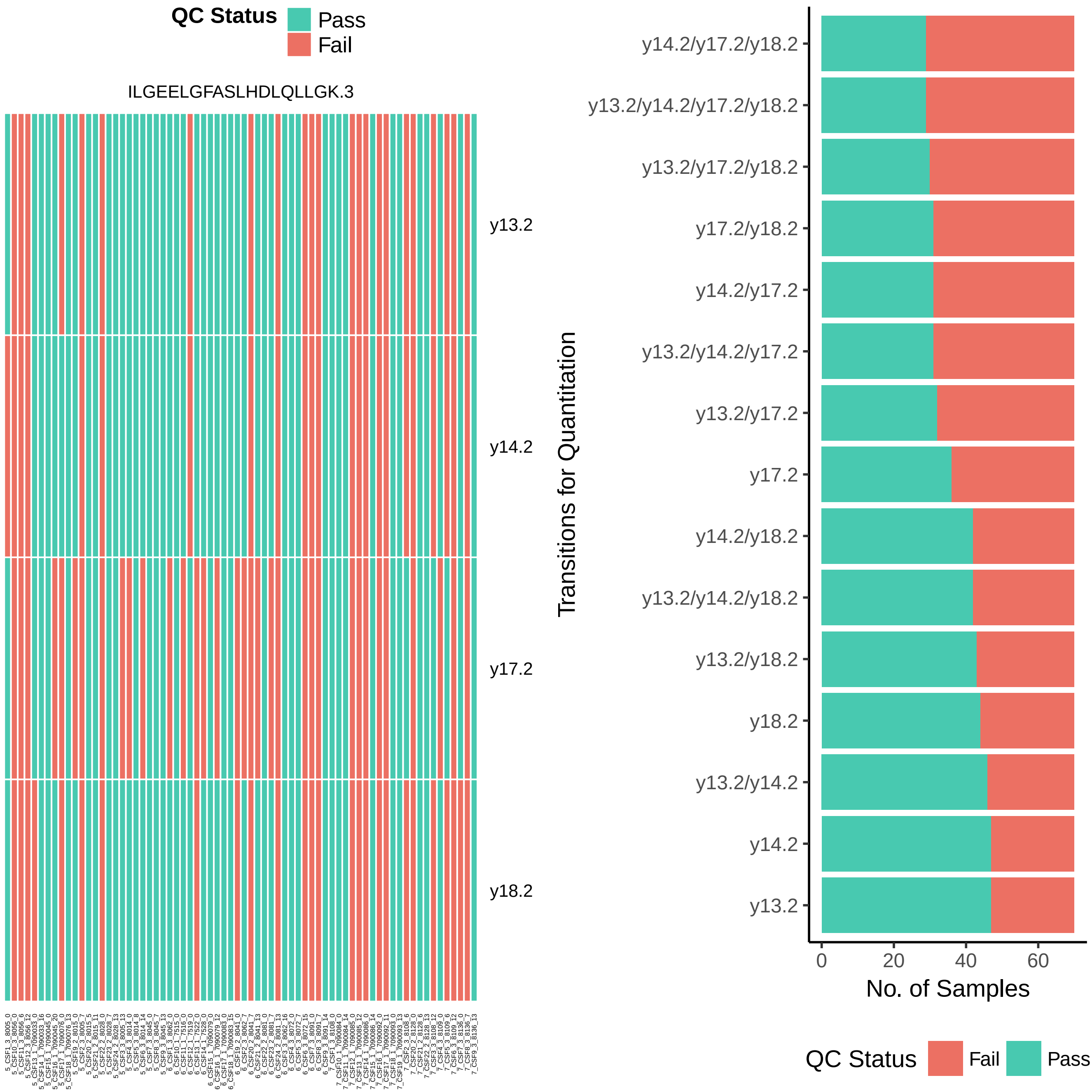
QC Status
Pass
Fail
ILGEELGFASLHDLQLLGK.3
y13.2
y14.2
y17.2
y18.2
y14.2/y17.2/y18.2
y13.2/y14.2/y17.2/y18.2
y13.2/y17.2/y18.2
y17.2/y18.2
y14.2/y17.2
y13.2/y14.2/y17.2
y13.2/y17.2
y17.2
y14.2/y18.2
y13.2/y14.2/y18.2
y13.2/y18.2
y18.2
y13.2/y14.2
y14.2
y13.2
Transitions for Quantitation
0
20
40
60
No. of Samples
QC Status
Fail
Pass

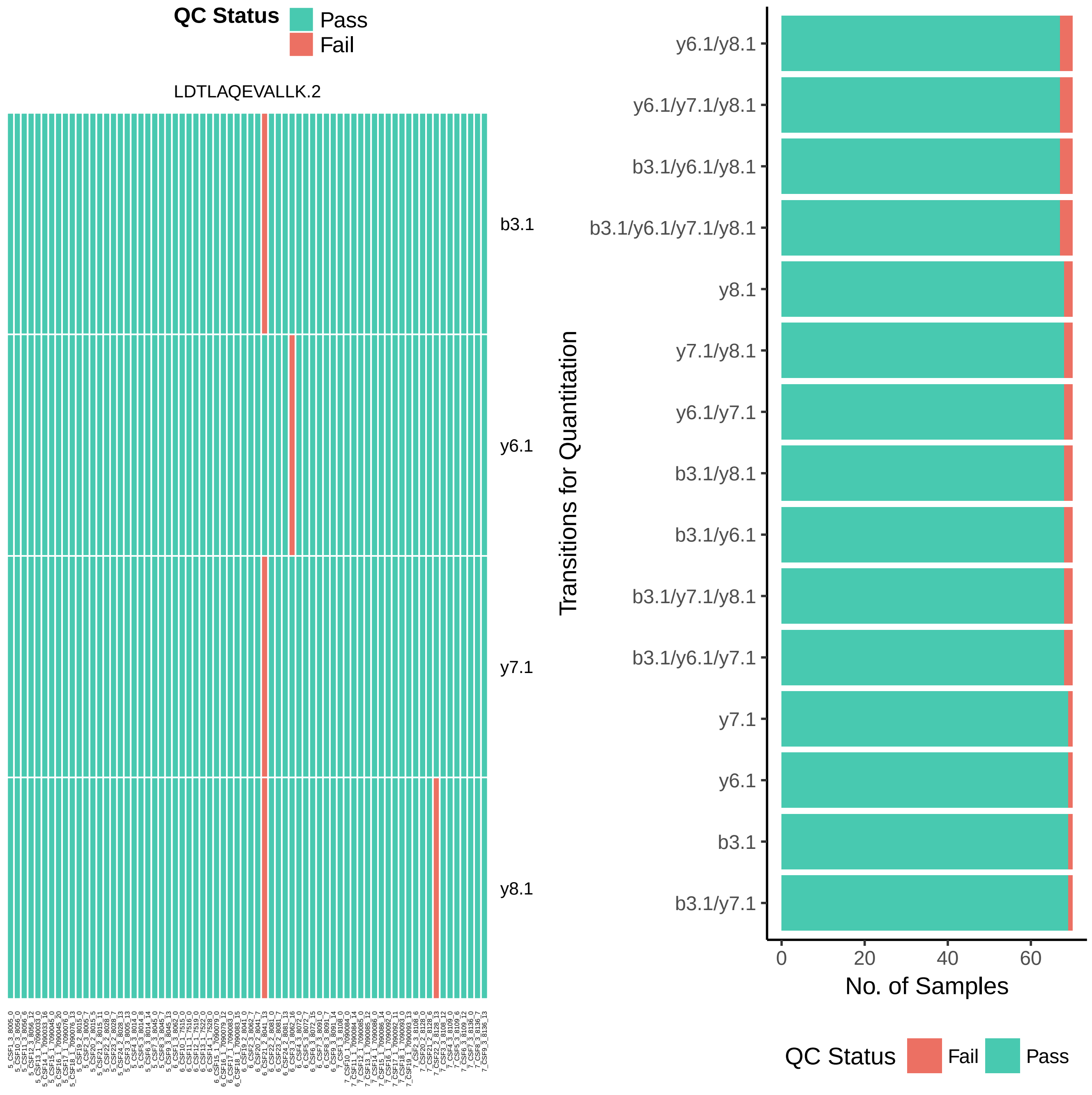
QC Status
Pass
Fail
LDTLAQEVALLK.2
b3.1
y6.1
y7.1
y8.1
y6.1/y8.1
y6.1/y7.1/y8.1
b3.1/y6.1/y8.1
b3.1/y6.1/y7.1/y8.1
y8.1
y7.1/y8.1
y6.1/y7.1
b3.1/y8.1
b3.1/y6.1
b3.1/y7.1/y8.1
b3.1/y6.1/y7.1
y7.1
y6.1
b3.1
b3.1/y7.1
Transitions for Quantitation
0
20
40
60
No. of Samples
QC Status
Fail
Pass

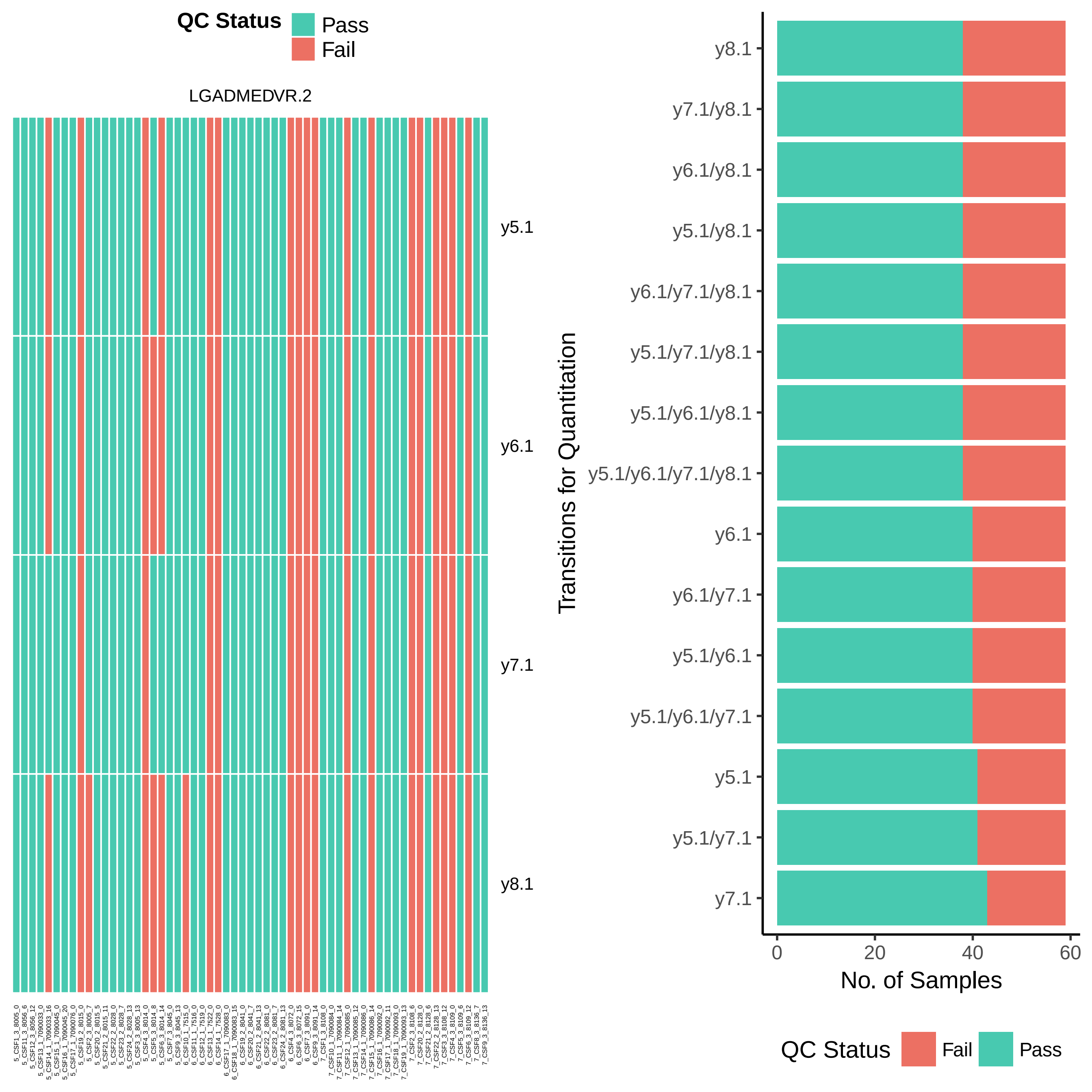
QC Status
Pass
Fail
LGADMEDVR.2
y5.1
y6.1
y7.1
y8.1
Transitions for Quantitation
y8.1
y7.1/y8.1
y6.1/y8.1
y5.1/y8.1
y6.1/y7.1/y8.1
y5.1/y7.1/y8.1
y5.1/y6.1/y8.1
y5.1/y6.1/y7.1/y8.1
y6.1
y6.1/y7.1
y5.1/y6.1
y5.1/y6.1/y7.1
y5.1
y5.1/y7.1
y7.1
0
20
40
60
No. of Samples
QC Status
Fail
Pass

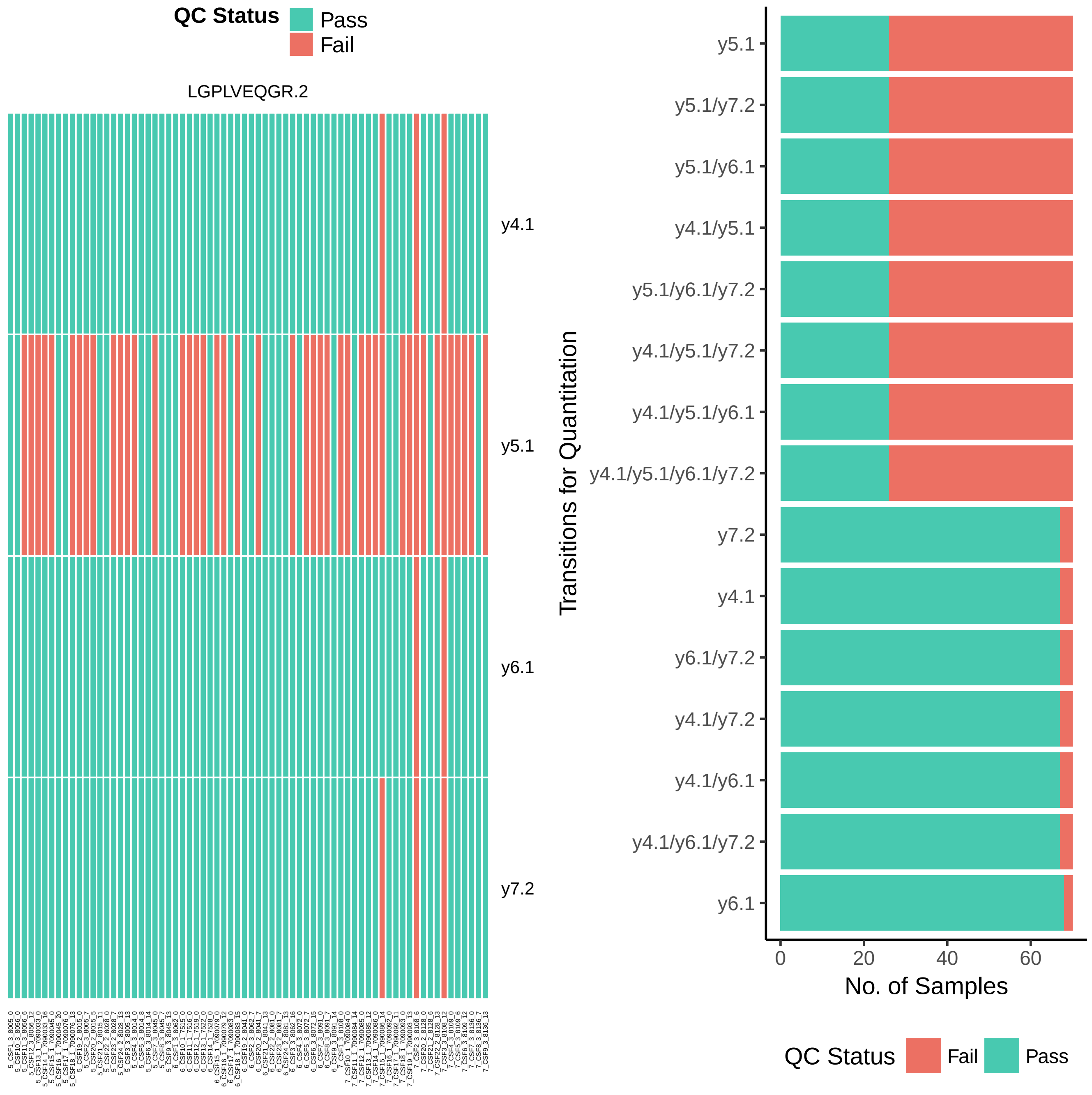
QC Status
Pass
Fail
LGPLVEQGR.2
y4.1
y5.1
y6.1
y7.2
Transitions for Quantitation
y5.1
y5.1/y7.2
y5.1/y6.1
y4.1/y5.1
y5.1/y6.1/y7.2
y4.1/y5.1/y7.2
y4.1/y5.1/y6.1
y4.1/y5.1/y6.1/y7.2
y7.2
y4.1
y6.1/y7.2
y4.1/y7.2
y4.1/y6.1
y4.1/y6.1/y7.2
y6.1
0
20
40
60
No. of Samples
QC Status
Fail
Pass

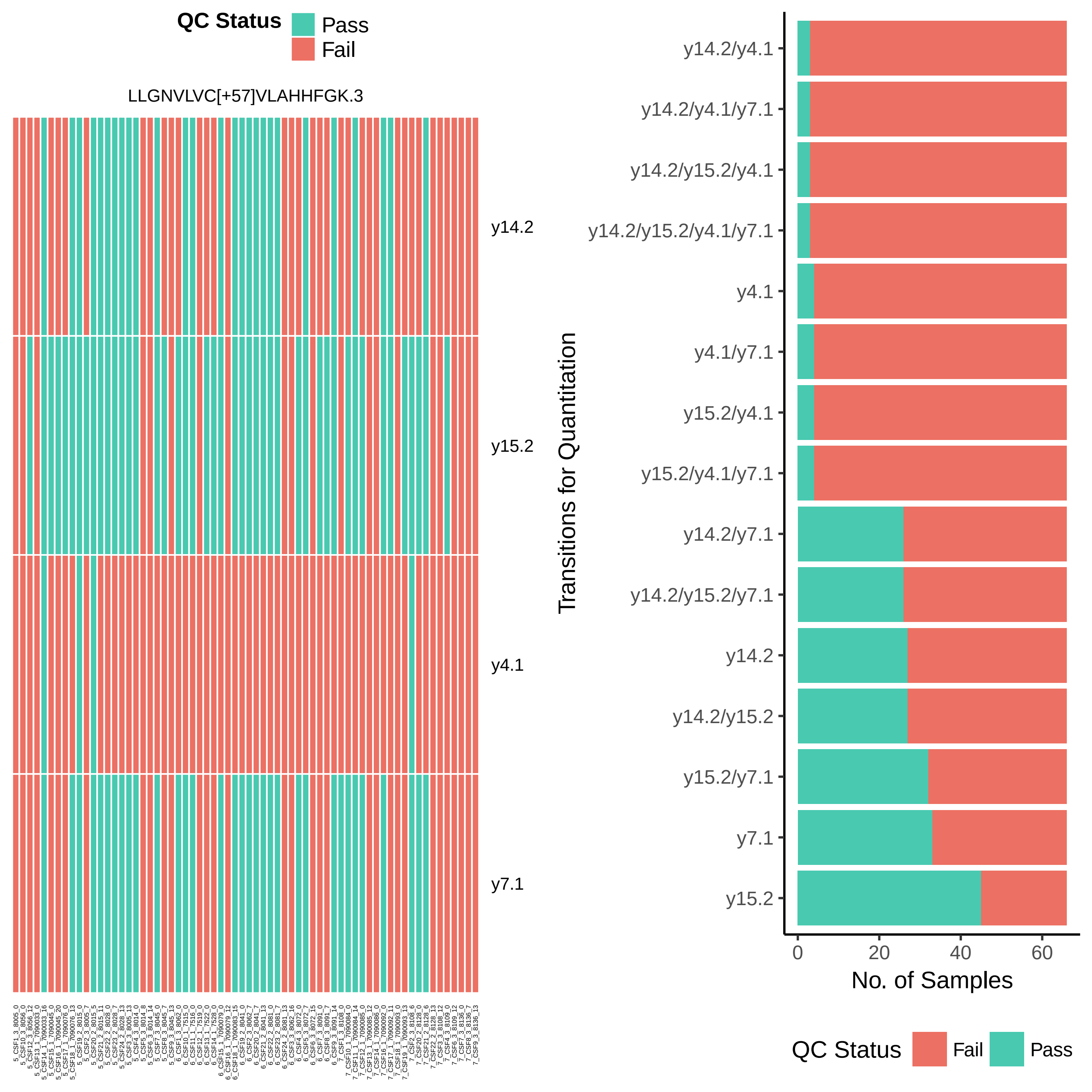
QC Status
Pass
Fail
LLGNVLVC[+57]VLAHHFGK.3
y14.2
y15.2
y4.1
y7.1
y14.2/y4.1
y14.2/y4.1/y7.1
y14.2/y15.2/y4.1
y14.2/y15.2/y4.1/y7.1
y4.1
y4.1/y7.1
y15.2/y4.1
y15.2/y4.1/y7.1
y14.2/y7.1
y14.2/y15.2/y7.1
y14.2
y14.2/y15.2
y15.2/y7.1
y7.1
y15.2
Transitions for Quantitation
0
20
40
60
No. of Samples
QC Status
Fail
Pass

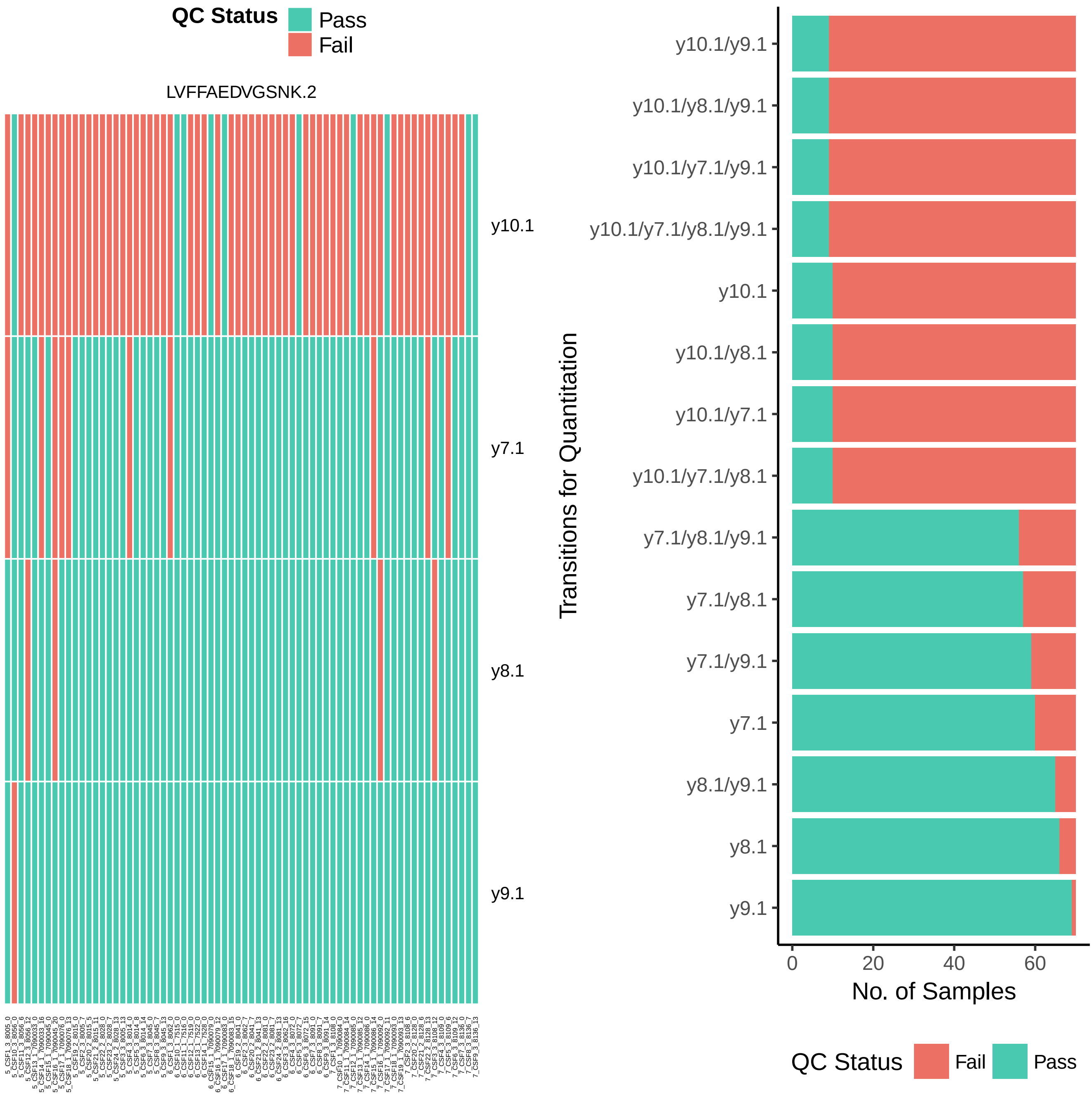
QC Status
Pass
Fail
LVFFAEDVGSNK.2
y10.1
y7.1
y8.1
y9.1
y10.1/y9.1
y10.1/y8.1/y9.1
y10.1/y7.1/y9.1
y10.1/y7.1/y8.1/y9.1
y10.1
y10.1/y8.1
y10.1/y7.1
y10.1/y7.1/y8.1
y7.1/y8.1/y9.1
y7.1/y8.1
y7.1/y9.1
y7.1
y8.1/y9.1
y8.1
y9.1
Transitions for Quantitation
0
20
40
60
No. of Samples
QC Status
Fail
Pass

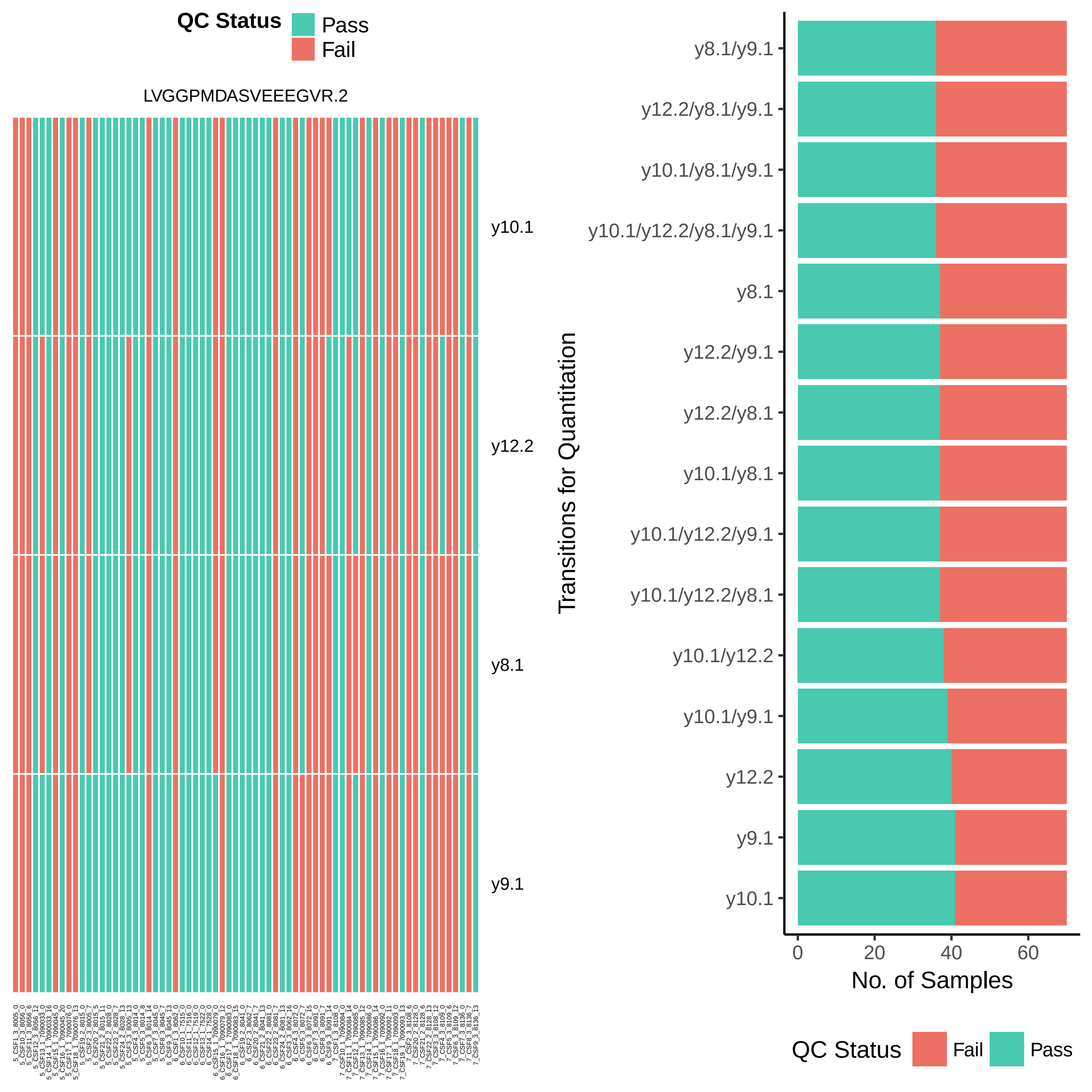

QC Status
Pass
Fail
LVGGPMDASVEEEGVR.2
y10.1
y12.2
y8.1
y9.1
Transitions for Quantitation
y8.1/y9.1
y12.2/y8.1/y9.1
y10.1/y8.1/y9.1
y10.1/y12.2/y8.1/y9.1
y8.1
y12.2/y9.1
y12.2/y8.1
y10.1/y8.1
y10.1/y12.2/y9.1
y10.1/y12.2/y8.1
y10.1/y12.2
y10.1/y9.1
y12.2
y9.1
y10.1
0
20
40
60
No. of Samples
QC Status
Fail
Pass

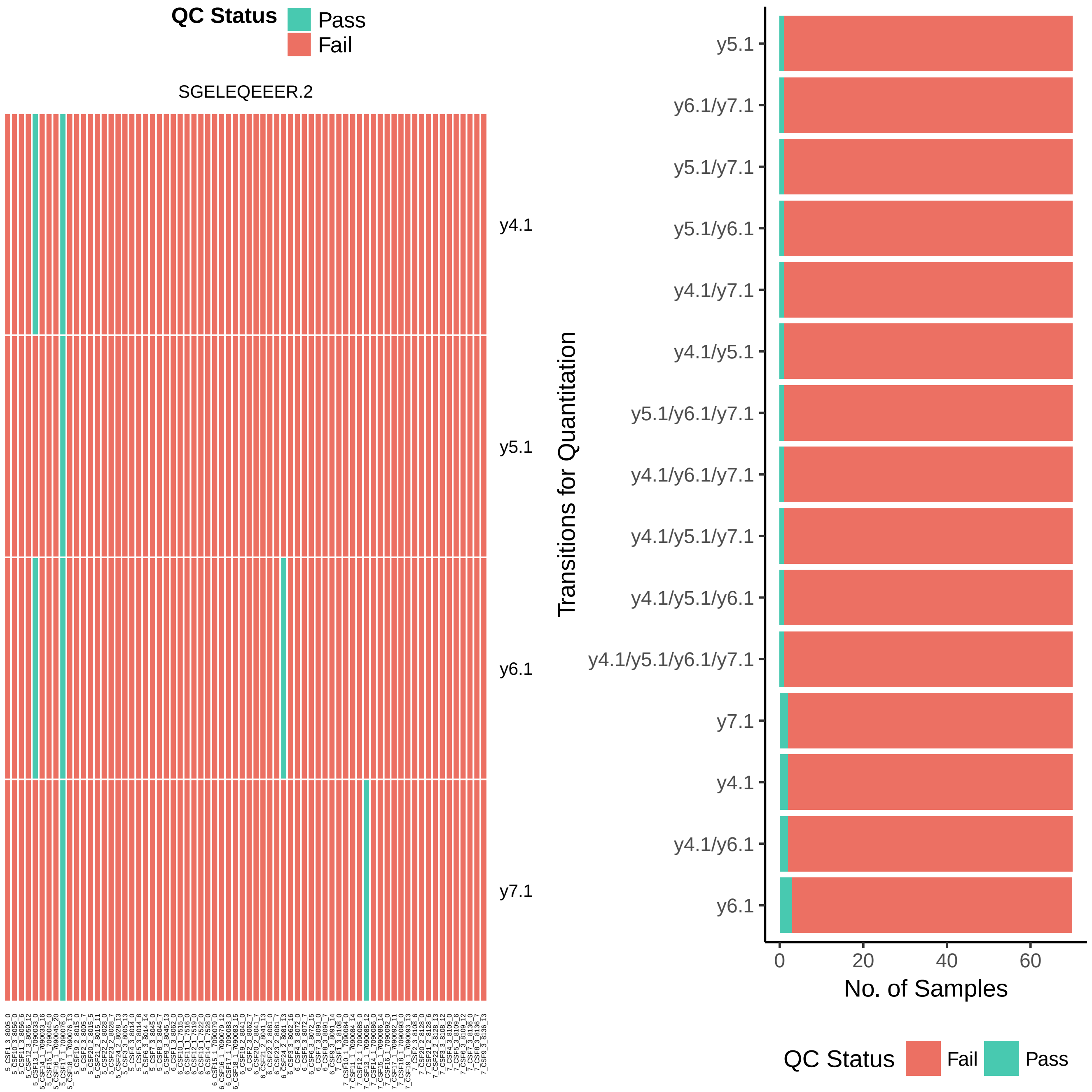
QC Status
Pass
Fail
SGELEQEEER.2
y4.1
y5.1
y6.1
y7.1
Transitions for Quantitation
y5.1
y6.1/y7.1
y5.1/y7.1
y5.1/y6.1
y4.1/y7.1
y4.1/y5.1
y5.1/y6.1/y7.1
y4.1/y6.1/y7.1
y4.1/y5.1/y7.1
y4.1/y5.1/y6.1
y4.1/y5.1/y6.1/y7.1
y7.1
y4.1
y4.1/y6.1
y6.1
0
20
40
60
No. of Samples
QC Status
Fail
Pass

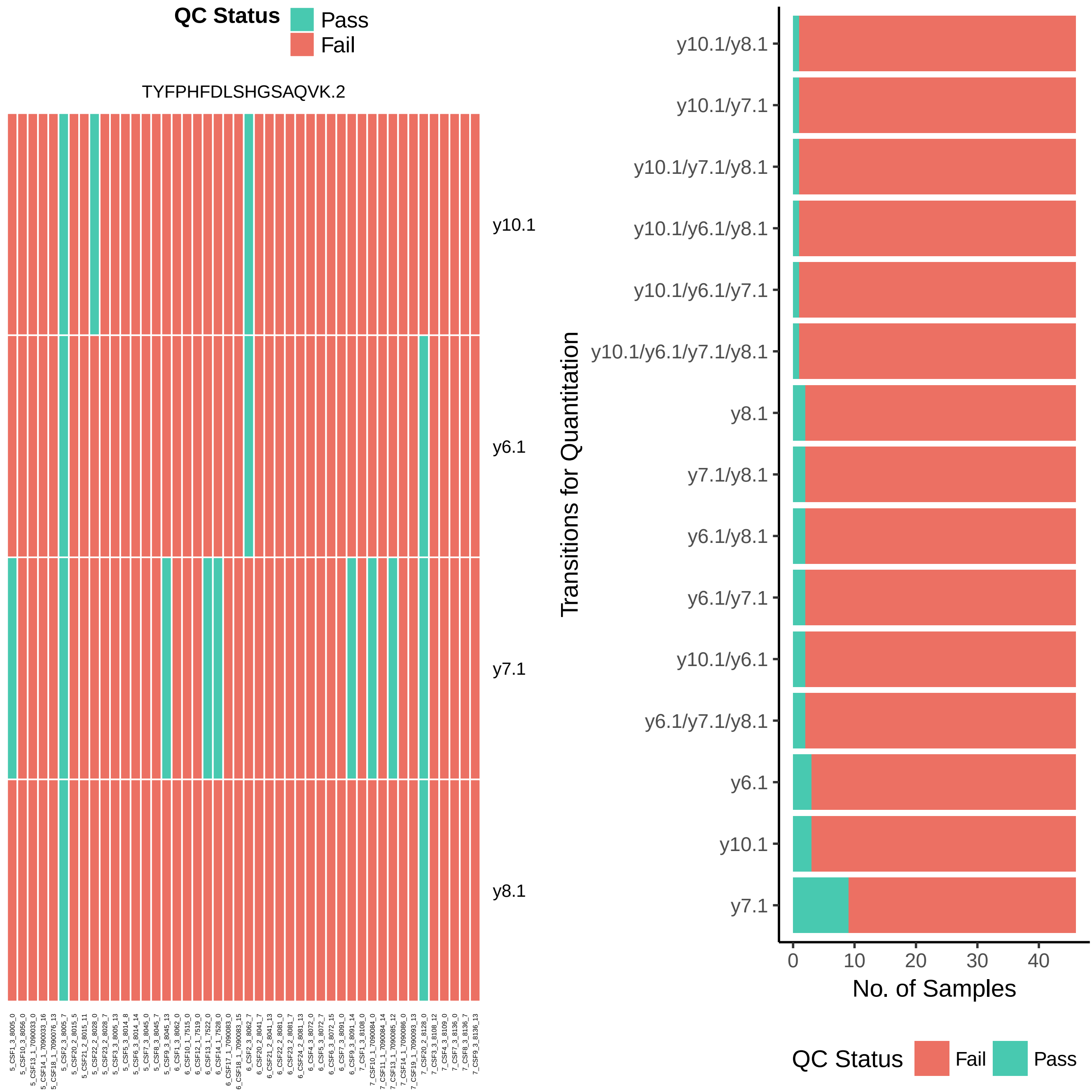

QC Status
Pass
Fail
TYFPHFDLSHGSAQVK.2
y10.1
y6.1
y7.1
y8.1
5_CSF1_3_8005_0
5_CSF10_3_8056_0
5_CSF13_1_7090033_0
5_CSF14_1_7090033_16
5_CSF18_1_7090076_13
5_CSF2_3_8005_7
5_CSF20_2_8015_5
5_CSF21_2_8015_11
5_CSF22_2_8028_0
5_CSF23_2_8028_7
5_CSF3_3_8005_13
5_CSF5_3_8014_8
5_CSF6_3_8014_14
5_CSF7_3_8045_0
5_CSF8_3_8045_7
5_CSF9_3_8045_13
6_CSF1_3_8062_0
6_CSF10_1_7515_0
6_CSF12_1_7519_0
6_CSF13_1_7522_0
6_CSF14_1_7528_0
6_CSF17_1_7090083_0
6_CSF18_1_7090083_15
6_CSF2_3_8062_7
6_CSF20_2_8041_7
6_CSF21_2_8041_13
6_CSF22_2_8081_0
6_CSF23_2_8081_7
6_CSF24_2_8081_13
6_CSF4_3_8072_0
6_CSF5_3_8072_7
6_CSF6_3_8072_15
6_CSF7_3_8091_0
6_CSF9_3_8091_14
7_CSF1_3_8108_0
7_CSF10_1_7090084_0
7_CSF11_1_7090084_14
7_CSF13_1_7090085_12
7_CSF14_1_7090086_0
7_CSF19_1_7090093_13
7_CSF20_2_8128_0
7_CSF3_3_8108_12
7_CSF4_3_8109_0
7_CSF7_3_8136_0
7_CSF8_3_8136_7
7_CSF9_3_8136_13
Transitions for Quantitation
y10.1/y8.1
y10.1/y7.1
y10.1/y7.1/y8.1
y10.1/y6.1/y8.1
y10.1/y6.1/y7.1
y10.1/y6.1/y7.1/y8.1
y8.1
y7.1/y8.1
y6.1/y8.1
y6.1/y7.1
y10.1/y6.1
y6.1/y7.1/y8.1
y6.1
y10.1
y7.1
0
10
20
30
40
No. of Samples
QC Status
Fail
Pass

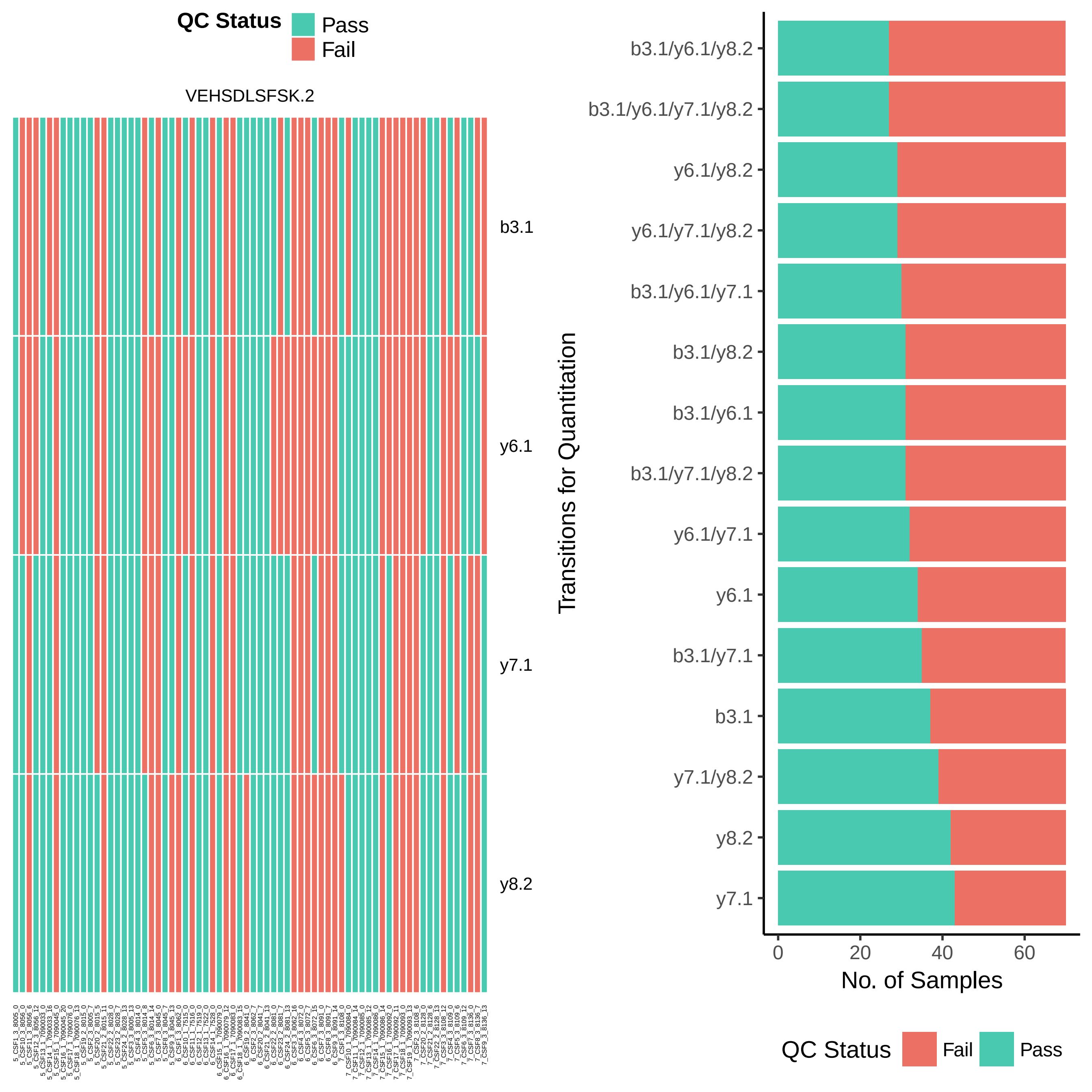

QC Status
Pass
Fail
VEHSDLSFSK.2
b3.1
y6.1
y7.1
y8.2
Transitions for Quantitation
b3.1/y6.1/y8.2
b3.1/y6.1/y7.1/y8.2
y6.1/y8.2
y6.1/y7.1/y8.2
b3.1/y6.1/y7.1
b3.1/y8.2
b3.1/y6.1
b3.1/y7.1/y8.2
y6.1/y7.1
y6.1
b3.1/y7.1
b3.1
y7.1/y8.2
y8.2
y7.1
0
20
40
60
No. of Samples
QC Status
Fail
Pass

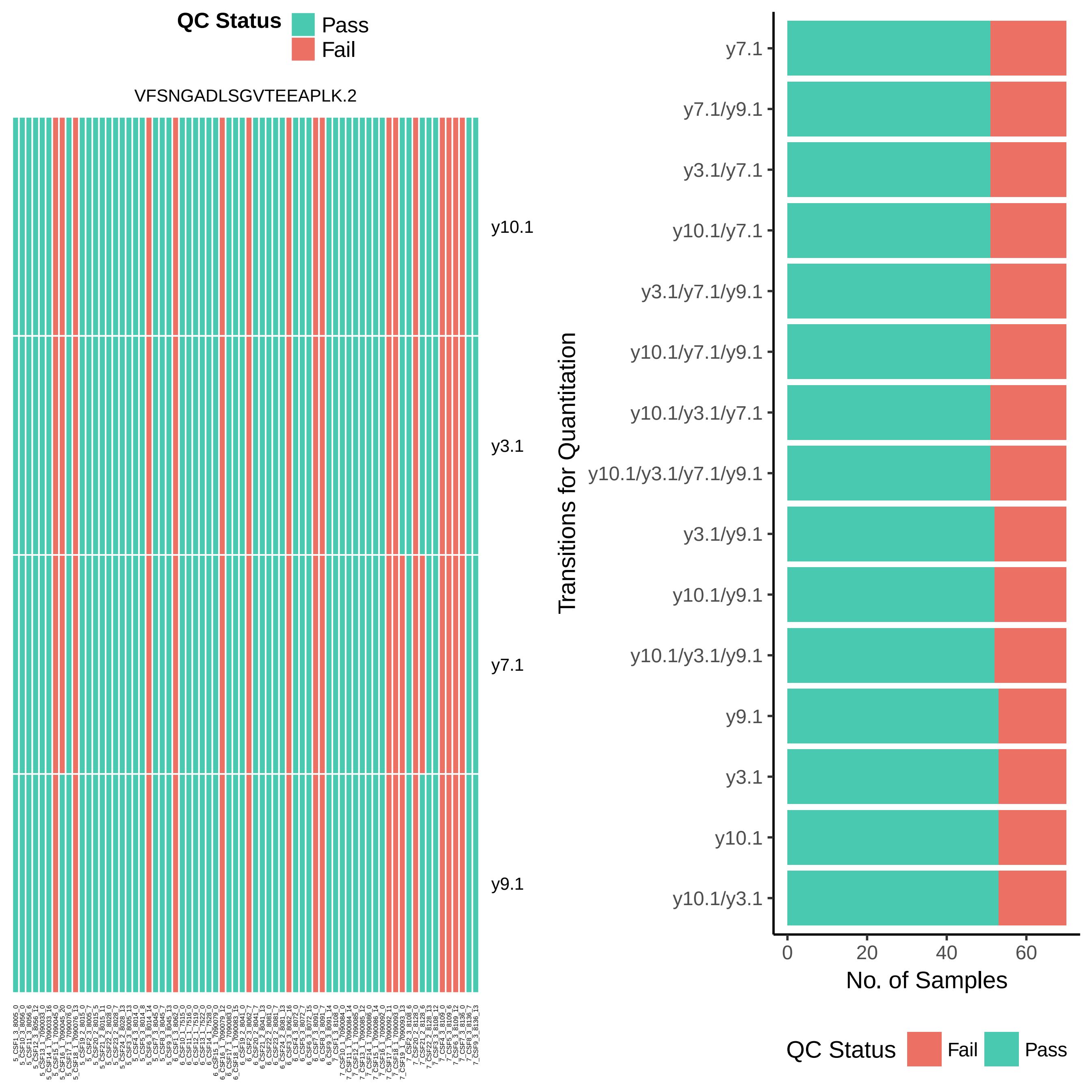
QC Status
Pass
Fail
VFSNGADLSGVTEEAPLK.2
y10.1
y3.1
y7.1
y9.1
Transitions for Quantitation
y7.1
y7.1/y9.1
y3.1/y7.1
y10.1/y7.1
y3.1/y7.1/y9.1
y10.1/y7.1/y9.1
y10.1/y3.1/y7.1
y10.1/y3.1/y7.1/y9.1
y3.1/y9.1
y10.1/y9.1
y10.1/y3.1/y9.1
y9.1
y3.1
y10.1
y10.1/y3.1
0
20
40
60
No. of Samples
QC Status
Fail
Pass

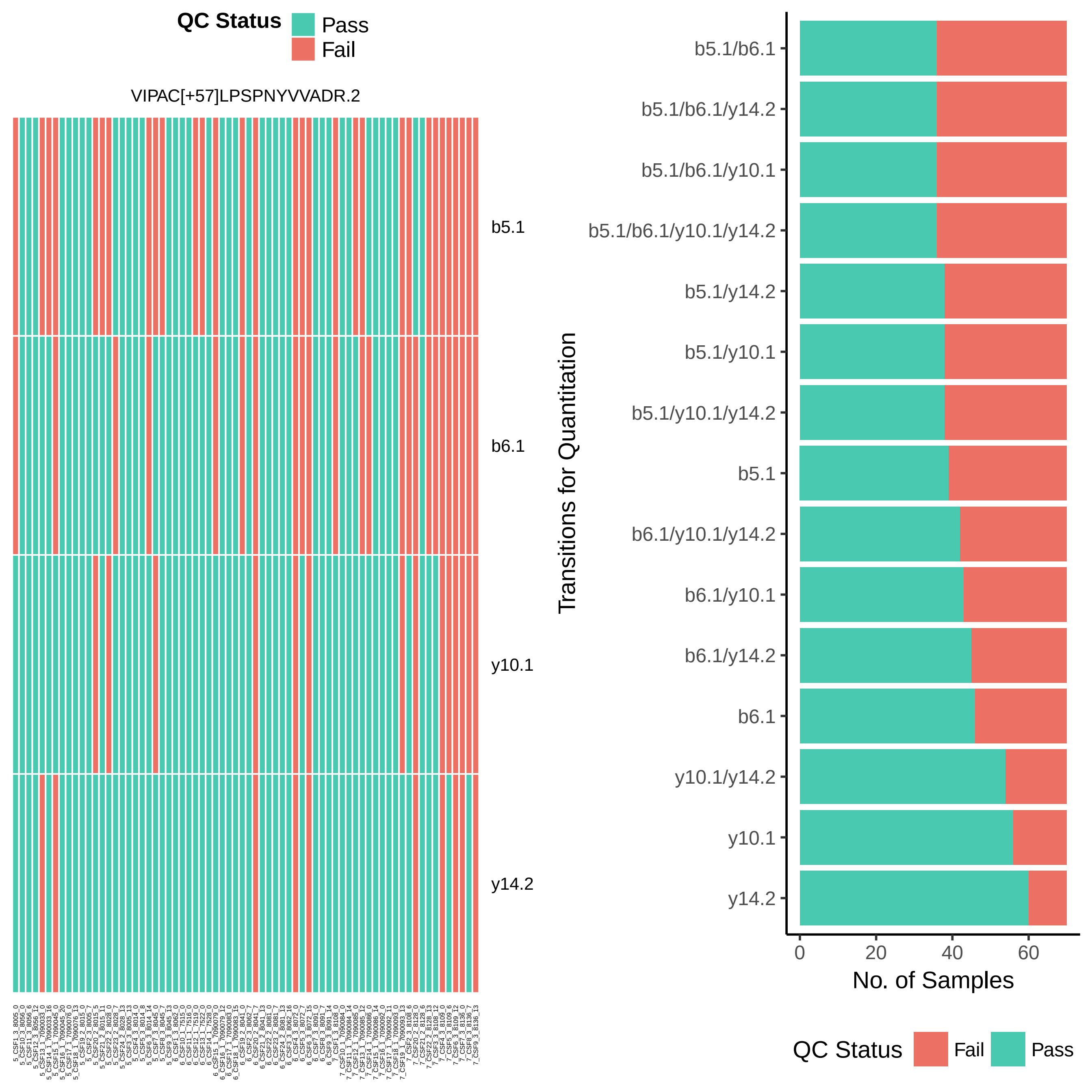
QC Status
Pass
Fail
VIPAC[+57]LPSPNYVVADR.2
b5.1
b6.1
y10.1
y14.2
b5.1/b6.1
b5.1/b6.1/y14.2
b5.1/b6.1/y10.1
b5.1/b6.1/y10.1/y14.2
b5.1/y14.2
b5.1/y10.1
b5.1/y10.1/y14.2
b5.1
b6.1/y10.1/y14.2
b6.1/y10.1
b6.1/y14.2
b6.1
y10.1/y14.2
y10.1
y14.2
Transitions for Quantitation
0
20
40
60
No. of Samples
QC Status
Fail
Pass

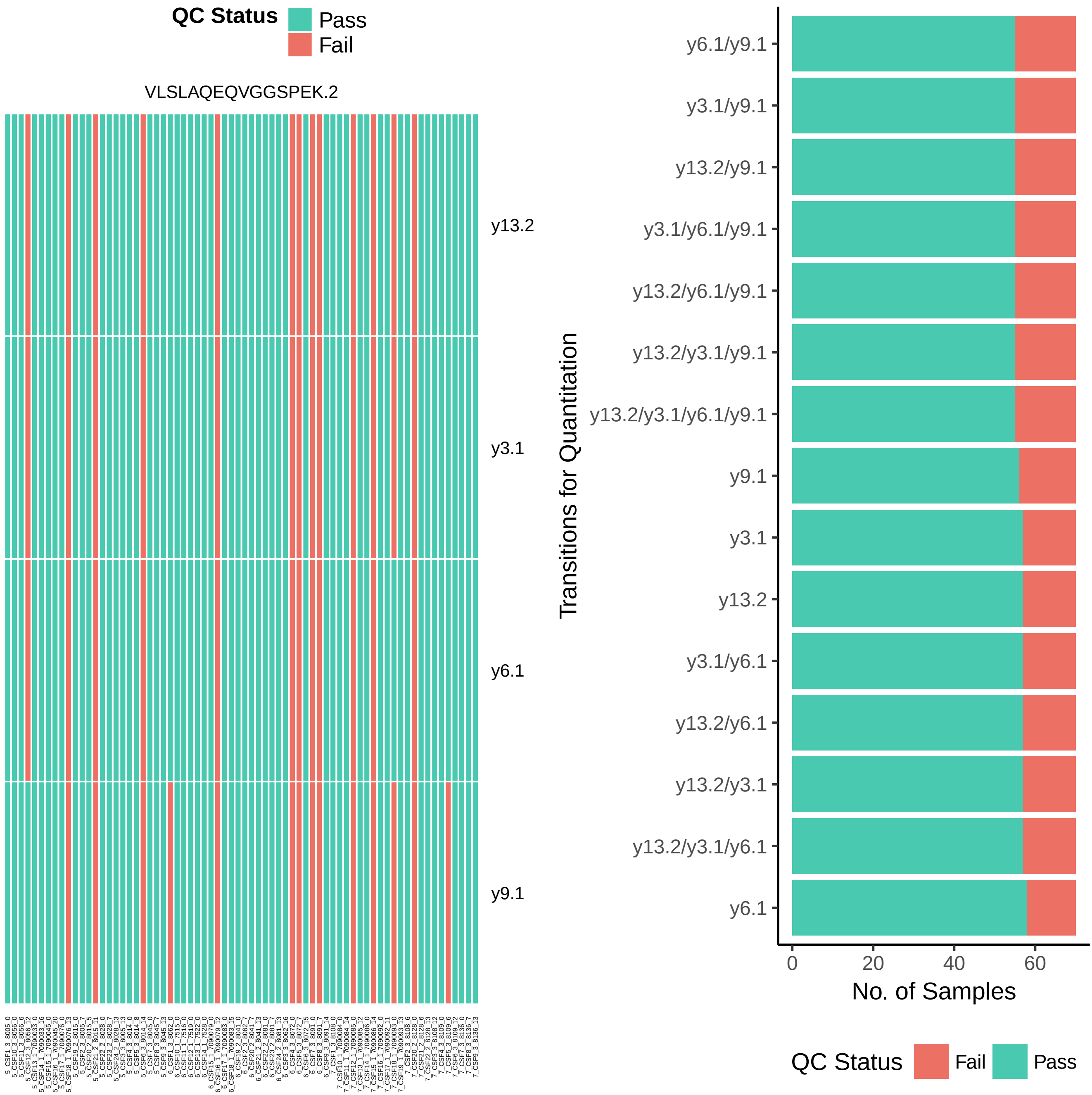
QC Status
Pass
Fail
VLSLAQEQVGGSPEK.2
y13.2
y3.1
y6.1
y9.1
Transitions for Quantitation
y6.1/y9.1
y3.1/y9.1
y13.2/y9.1
y3.1/y6.1/y9.1
y13.2/y6.1/y9.1
y13.2/y3.1/y9.1
y13.2/y3.1/y6.1/y9.1
y9.1
y3.1
y13.2
y3.1/y6.1
y13.2/y6.1
y13.2/y3.1
y13.2/y3.1/y6.1
y6.1
0
20
40
60
No. of Samples
QC Status
Fail
Pass

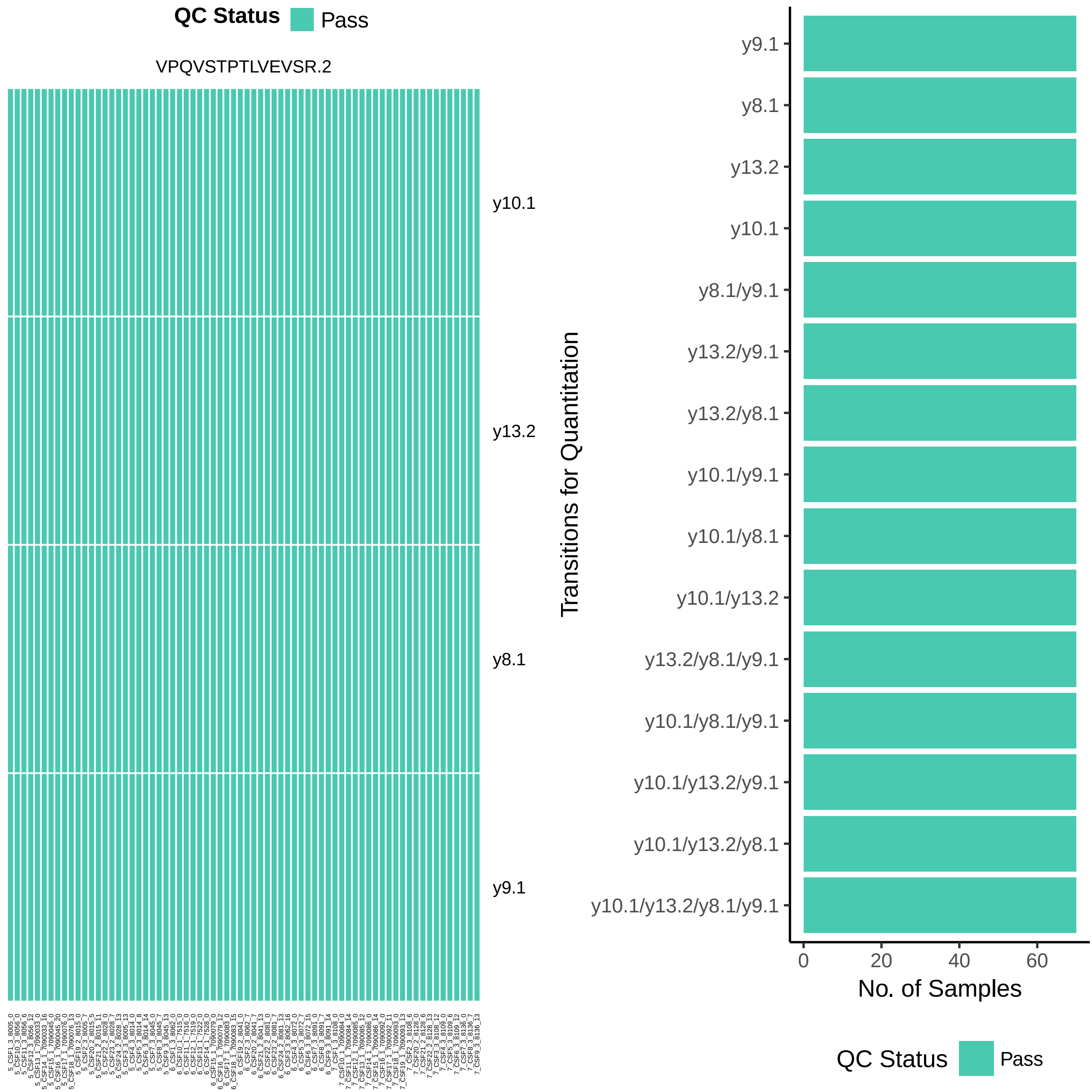

QC Status
Pass
VPQVSTPTLVEVSR.2
y10.1
y13.2
y8.1
y9.1
Transitions for Quantitation
y9.1
y8.1
y13.2
y10.1
y8.1/y9.1
y13.2/y9.1
y13.2/y8.1
y10.1/y9.1
y10.1/y8.1
y10.1/y13.2
y13.2/y8.1/y9.1
y10.1/y8.1/y9.1
y10.1/y13.2/y9.1
y10.1/y13.2/y8.1
y10.1/y13.2/y8.1/y9.1
0
20
40
60
No. of Samples
QC Status
Pass

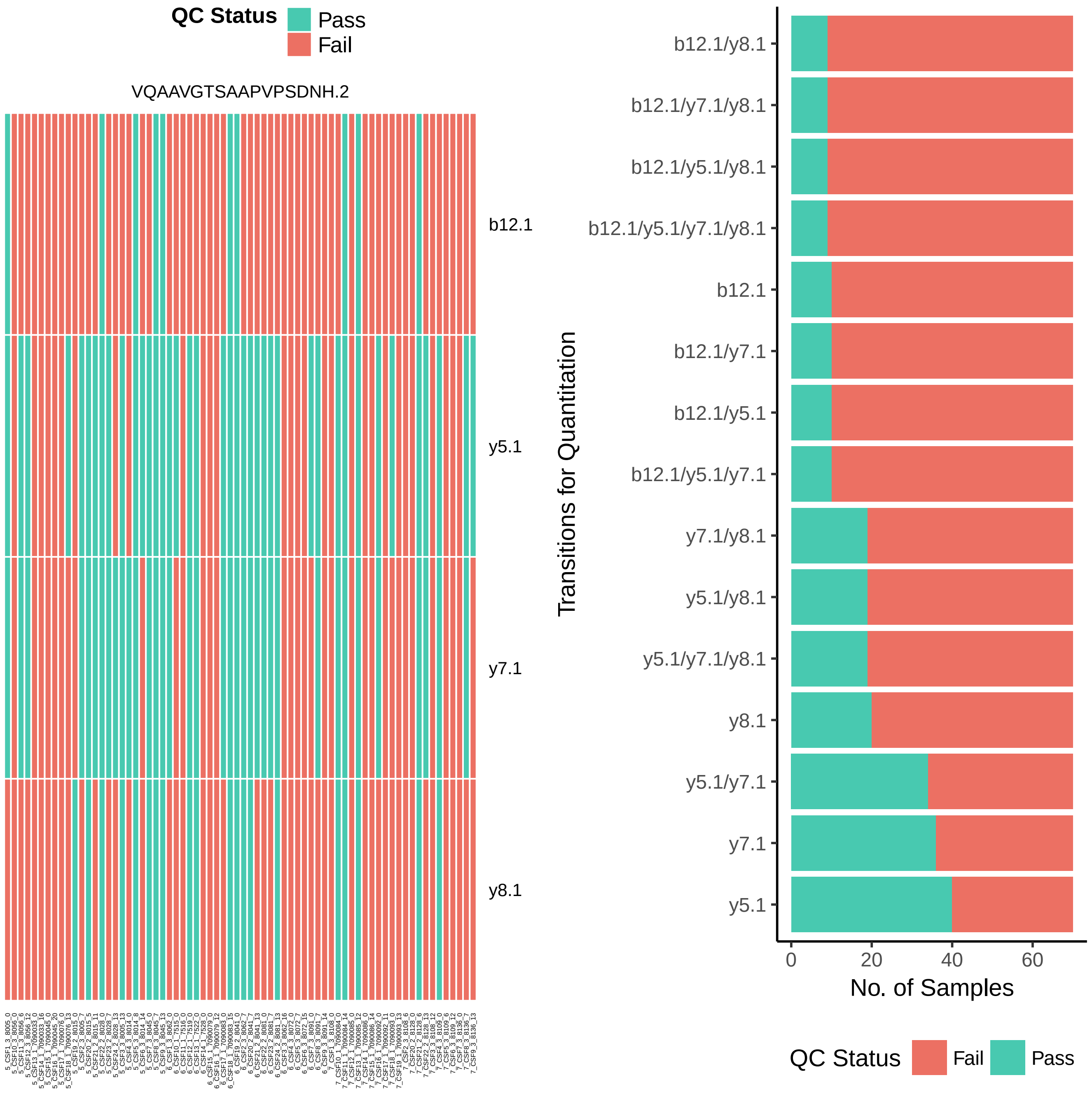
QC Status
Pass
Fail
VQAAVGTSAAPVPSDNH.2
b12.1
y5.1
y7.1
y8.1
Transitions for Quantitation
b12.1/y8.1
b12.1/y7.1/y8.1
b12.1/y5.1/y8.1
b12.1/y5.1/y7.1/y8.1
b12.1
b12.1/y7.1
b12.1/y5.1
b12.1/y5.1/y7.1
y7.1/y8.1
y5.1/y8.1
y5.1/y7.1/y8.1
y8.1
y5.1/y7.1
y7.1
y5.1
0
20
40
60
No. of Samples
QC Status
Fail
Pass

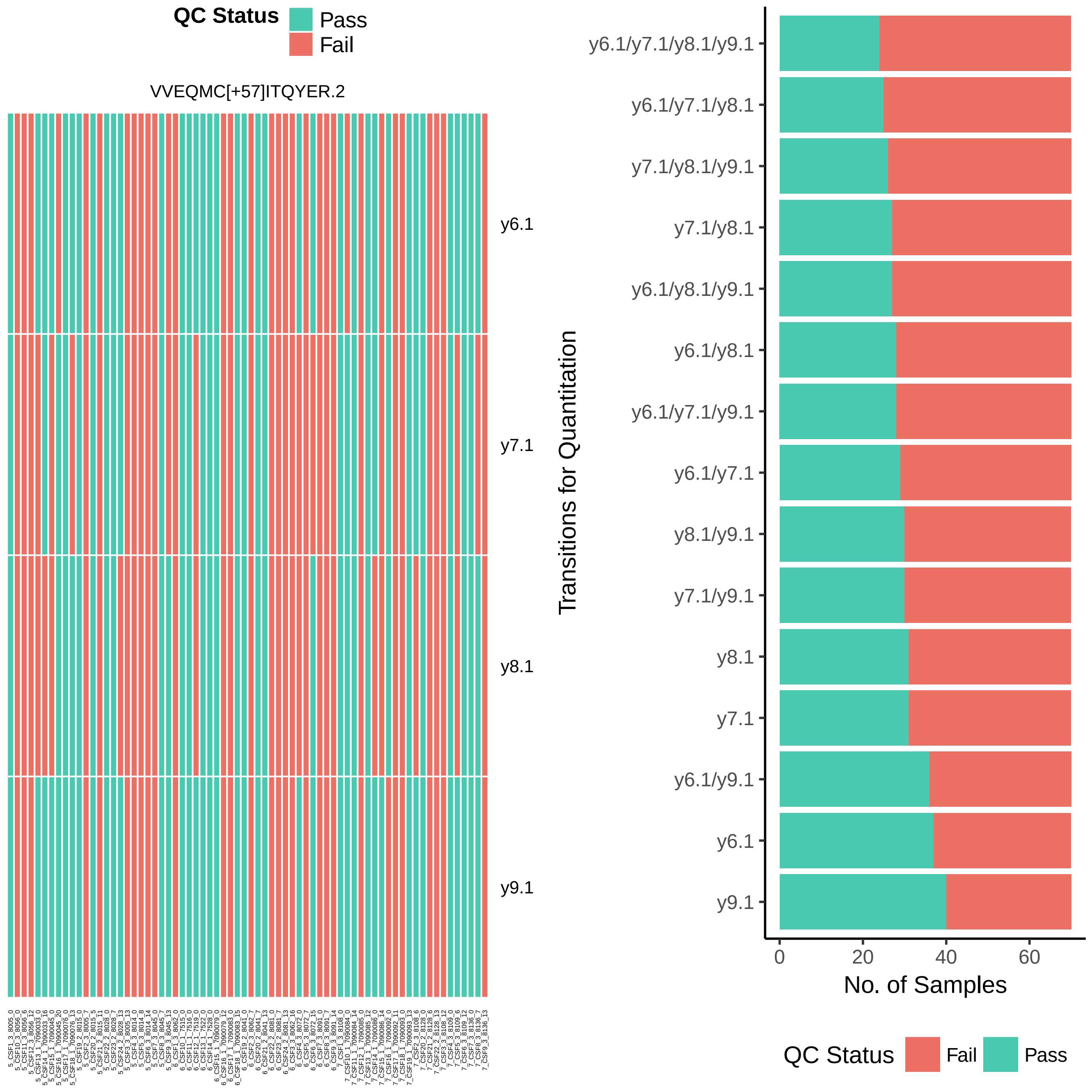

QC Status
Pass
Fail
VVEQMC[+57]ITQYER.2
y6.1
y7.1
y8.1
y9.1
Transitions for Quantitation
y6.1/y7.1/y8.1/y9.1
y6.1/y7.1/y8.1
y7.1/y8.1/y9.1
y7.1/y8.1
y6.1/y8.1/y9.1
y6.1/y8.1
y6.1/y7.1/y9.1
y6.1/y7.1
y8.1/y9.1
y7.1/y9.1
y8.1
y7.1
y6.1/y9.1
y6.1
y9.1
0
20
40
60
No. of Samples
QC Status
Fail
Pass

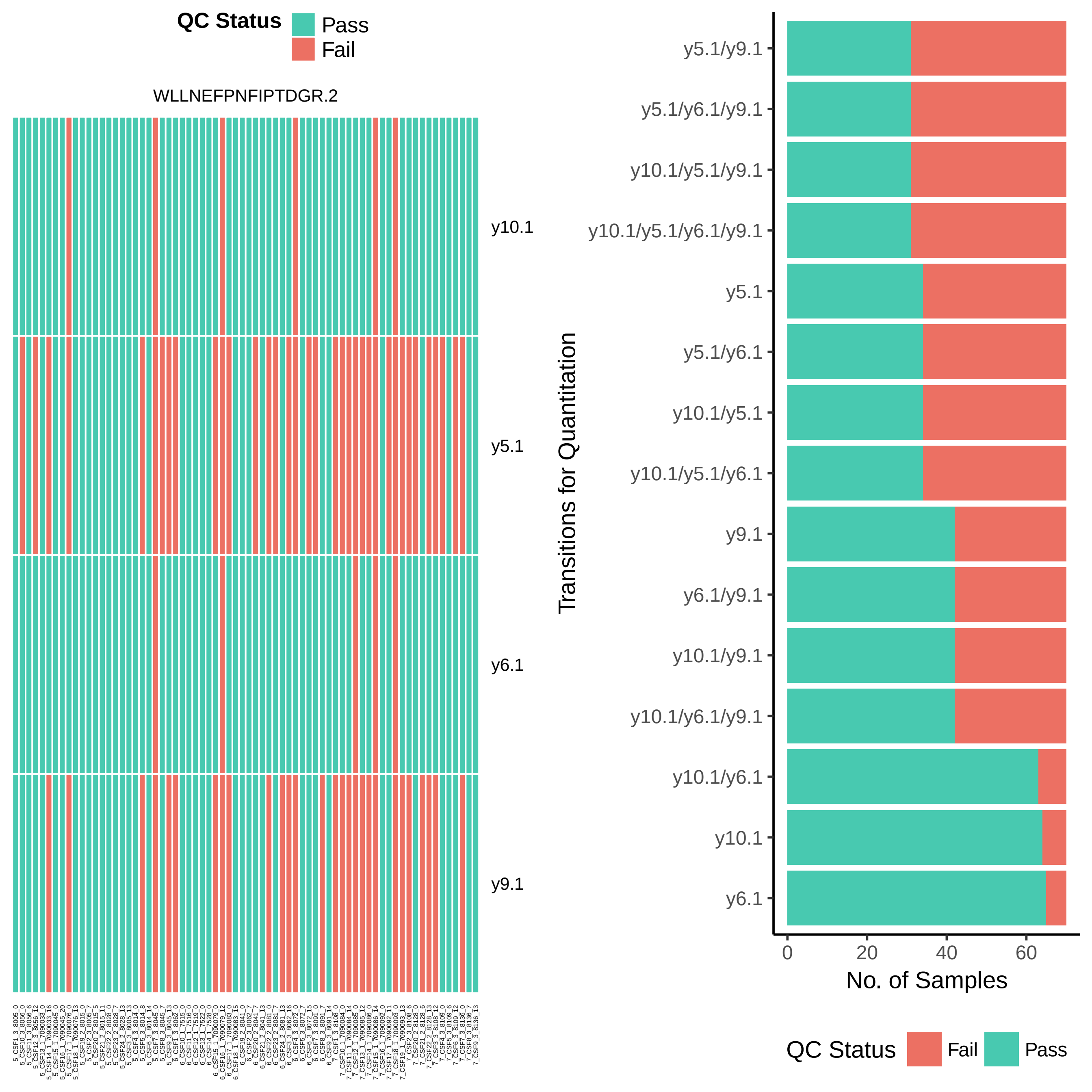
QC Status
Pass
Fail
WLLNEFPNFIPTDGR.2
y10.1
y5.1
y6.1
y9.1
y5.1/y9.1
y5.1/y6.1/y9.1
y10.1/y5.1/y9.1
y10.1/y5.1/y6.1/y9.1
y5.1
y5.1/y6.1
y10.1/y5.1
y10.1/y5.1/y6.1
y9.1
y6.1/y9.1
y10.1/y9.1
y10.1/y6.1/y9.1
y10.1/y6.1
y10.1
y6.1
Transitions for Quantitation
0
20
40
60
No. of Samples
QC Status
Fail
Pass

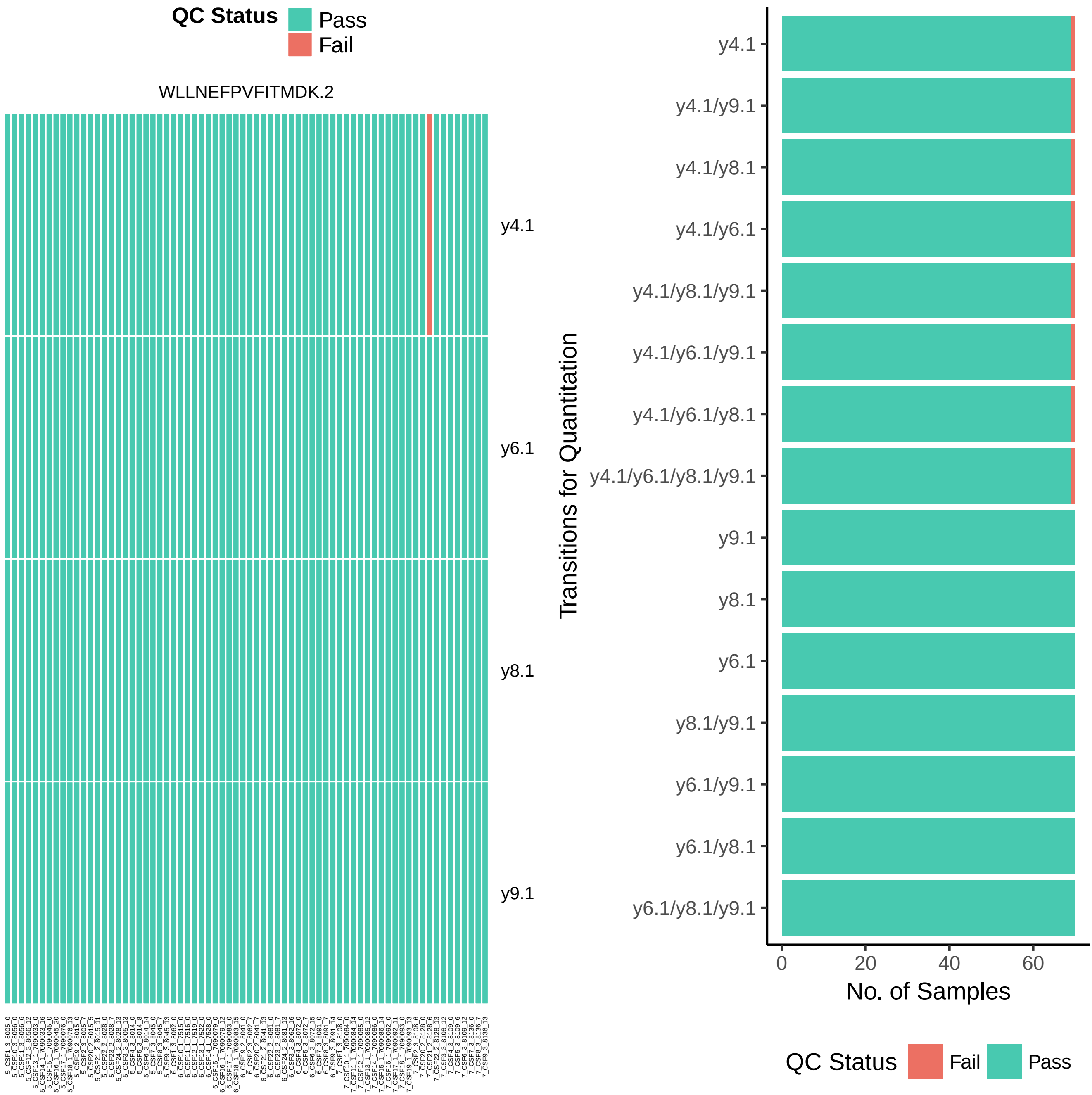
QC Status
Pass
Fail
WLLNEFPVFITMDK.2
y4.1
y6.1
y8.1
y9.1
Transitions for Quantitation
y4.1
y4.1/y9.1
y4.1/y8.1
y4.1/y6.1
y4.1/y8.1/y9.1
y4.1/y6.1/y9.1
y4.1/y6.1/y8.1
y4.1/y6.1/y8.1/y9.1
y9.1
y8.1
y6.1
y8.1/y9.1
y6.1/y9.1
y6.1/y8.1
y6.1/y8.1/y9.1
0
20
40
60
No. of Samples
QC Status
Fail
Pass

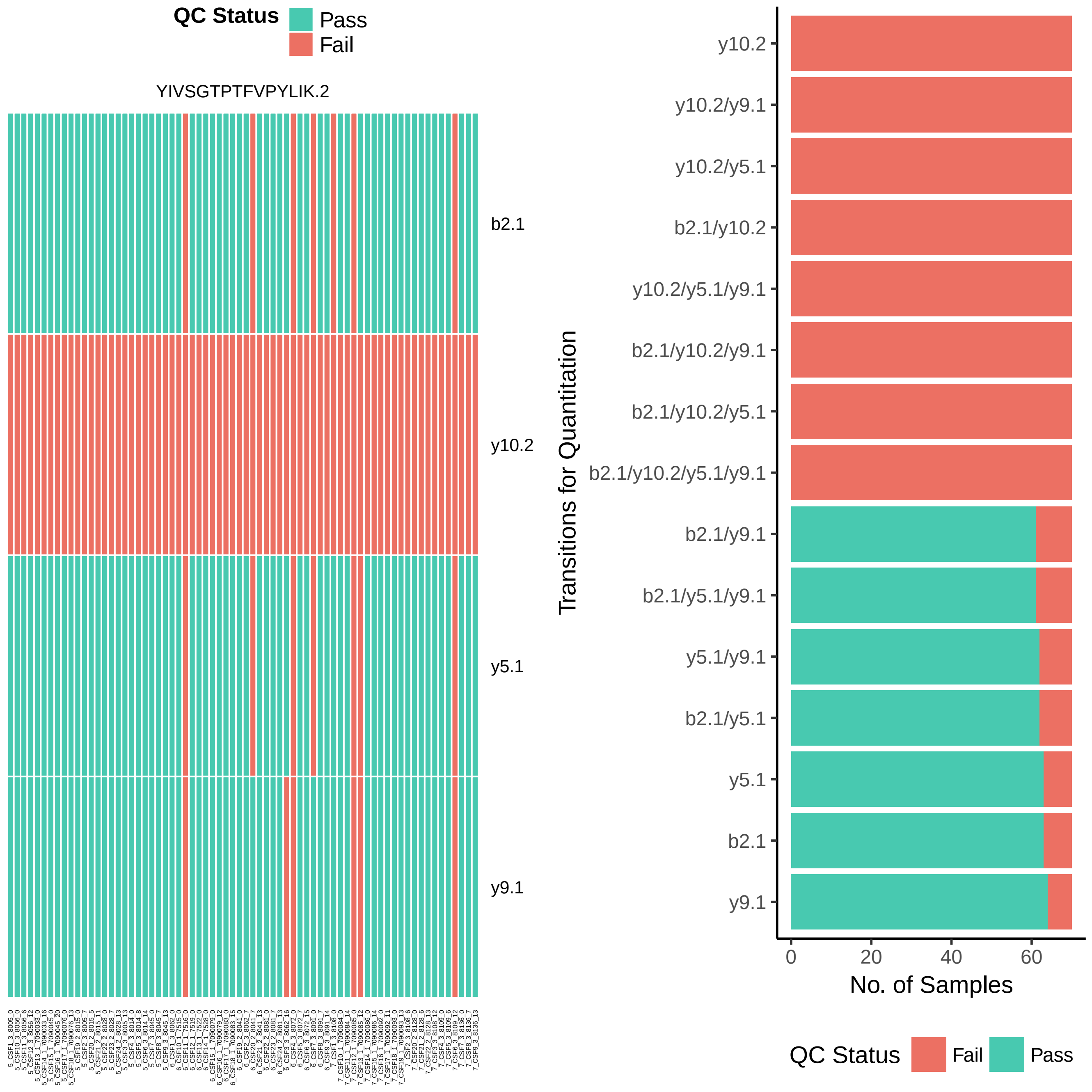
QC Status
Pass
Fail
YIVSGTPTFVPYLIK.2
b2.1
y10.2
y5.1
y9.1
Transitions for Quantitation
y10.2
y10.2/y9.1
y10.2/y5.1
b2.1/y10.2
y10.2/y5.1/y9.1
b2.1/y10.2/y9.1
b2.1/y10.2/y5.1
b2.1/y10.2/y5.1/y9.1
b2.1/y9.1
b2.1/y5.1/y9.1
y5.1/y9.1
b2.1/y5.1
y5.1
b2.1
y9.1
0
20
40
60
No. of Samples
QC Status
Fail
Pass

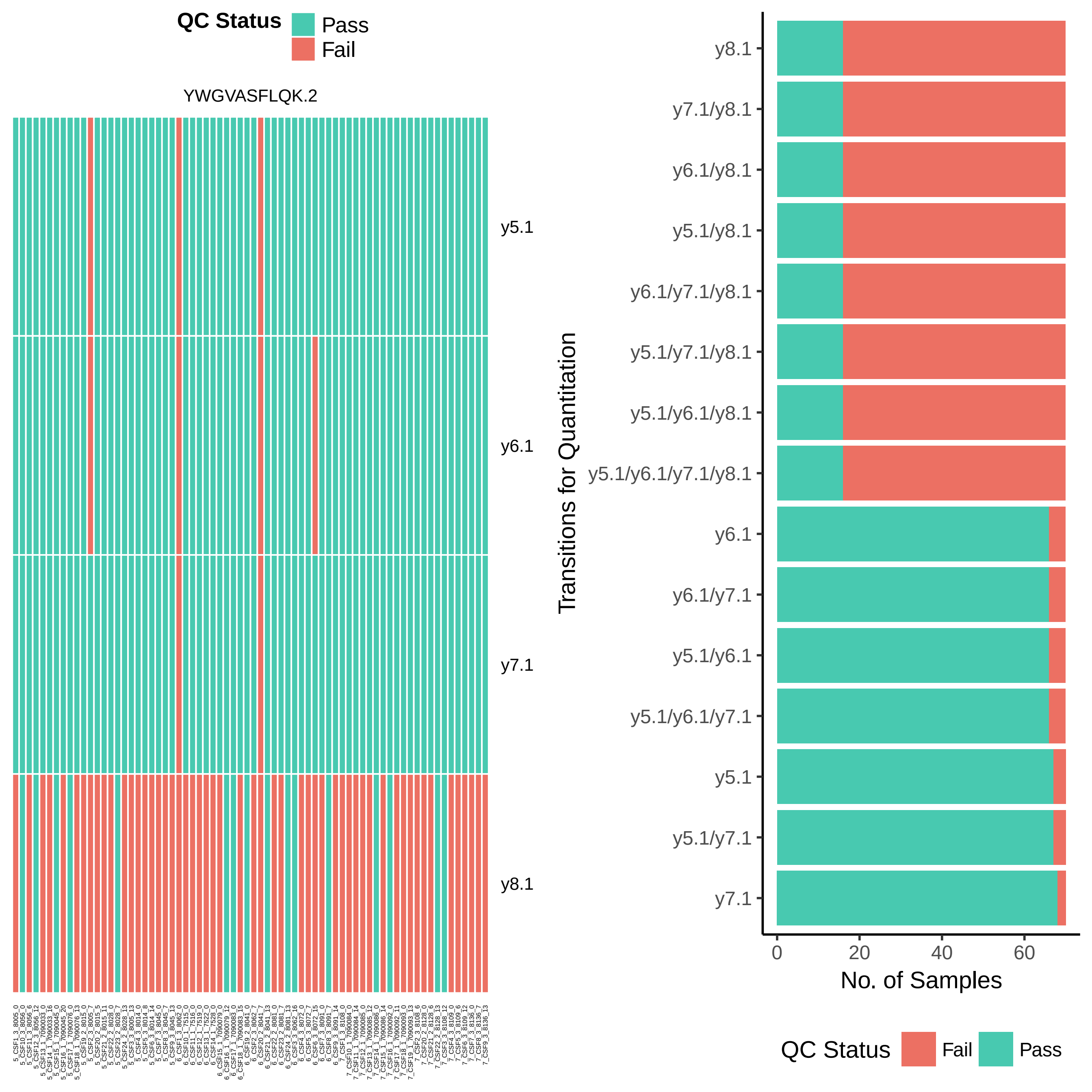

QC Status
Pass
Fail
YWGVASFLQK.2
y5.1
y6.1
y7.1
y8.1
Transitions for Quantitation
y8.1
y7.1/y8.1
y6.1/y8.1
y5.1/y8.1
y6.1/y7.1/y8.1
y5.1/y7.1/y8.1
y5.1/y6.1/y8.1
y5.1/y6.1/y7.1/y8.1
y6.1
y6.1/y7.1
y5.1/y6.1
y5.1/y6.1/y7.1
y5.1
y5.1/y7.1
y7.1
0
20
40
60
No. of Samples
QC Status
Fail
Pass
